# Boosting Low‐Valent Aluminum(I) Reactivity with a Potassium Reagent

**DOI:** 10.1002/anie.202006693

**Published:** 2020-06-29

**Authors:** Samuel Grams, Jonathan Eyselein, Jens Langer, Christian Färber, Sjoerd Harder

**Affiliations:** ^1^ Chair of Inorganic and Organometallic Chemistry Universität Erlangen-Nürnberg Egerlandstrasse 1 91058 Erlangen Germany

**Keywords:** aluminum, C−H activation, density-functional calculations, low-valent complexes, potassium

## Abstract

The reagent RK [R=CH(SiMe_3_)_2_ or N(SiMe_3_)_2_] was expected to react with the low‐valent (^DIPP^BDI)Al (^DIPP^BDI=HC[C(Me)N(DIPP)]_2_, DIPP=2,6‐*i*Pr‐phenyl) to give [(^DIPP^BDI)AlR]^−^K^+^. However, deprotonation of the Me group in the ligand backbone was observed and [H_2_C=C(N‐DIPP)−C(H)=C(Me)−N−DIPP]Al^−^K^+^ (**1**) crystallized as a bright‐yellow product (73 %). Like most anionic Al^I^ complexes, **1** forms a dimer in which formally negatively charged Al centers are bridged by K^+^ ions, showing strong K^+^⋅⋅⋅DIPP interactions. The rather short Al–K bonds [3.499(1)–3.588(1) Å] indicate tight bonding of the dimer. According to DOSY NMR analysis, **1** is dimeric in C_6_H_6_ and monomeric in THF, but slowly reacts with both solvents. In reaction with C_6_H_6_, two C−H bond activations are observed and a product with a *para*‐phenylene moiety was exclusively isolated. DFT calculations confirm that the Al center in **1** is more reactive than that in (^DIPP^BDI)Al. Calculations show that both Al^I^ and K^+^ work in concert and determines the reactivity of **1**.

## Graphical abstract

**Vitamin K for Al**: Attempts to enhance the reactivity of a β‐diketiminate Al^I^ complex by addition of a potassium reagent led to ligand deprotonation, yielding a different highly reactive anionic Al^I^ complex. Addition of benzene gave two C−H bond activations exclusively in *para*‐positions.

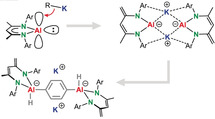

The first known low‐valent Al^I^ complex, (Cp*Al)_4_ (**I**),^1^ is in equilibrium with monomeric Cp*Al. This complex features a high‐lying HOMO and a low‐lying LUMO, a combination that leads to high reactivity and transition‐metal‐like properties.^2^, ^3^ Bulkier Cp ligands can direct the equilibrium exclusively to the more reactive monomer.^4^, ^5^ A kinetically stabilized monomeric Al^I^ complex, (^DIPP^BDI)Al (**II**), was isolated by the group of Roesky.^6^ Despite its protective bulky ^DIPP^BDI ligand, (^DIPP^BDI)Al is like an N‐heterocyclic carbene,^7^ a good σ‐donor and π‐acceptor exhibiting an unusually high reactivity that has been exploited in X−H and C−F bond activation.^8^

Recent years have seen the development of even more‐reactive anionic Al^I^ complexes. Given the theoretical prediction that the anionic N‐heterocyclic aluminenes [C(H)N(R)]_2_Al^−^ are isolobal to Arduengo carbenes and should be stable,^9^ the groups of Aldridge and Goicoechea presented the first anionic Al^I^ complex, which crystallized as a dimer with bridging K^+^ ions (**III** 
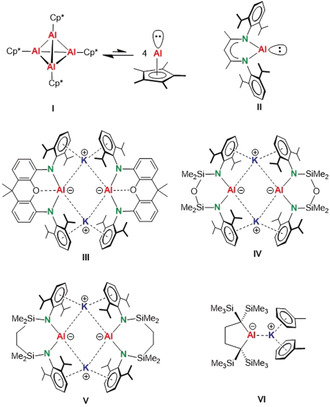
).^10^ They demonstrated its unique reactivity as exemplified by the first oxidative addition of a benzene C−H bond to a single main‐group metal center and, even more spectacular, C−C bond cleavage in benzene.^11^ Subsequent work of the groups of Coles and Hill led to similar dimeric *bis*‐amido Al^I^ complexes, **IV** and **V**, respectively.^12^, ^13^ Most recently, Yamashita and co‐workers isolated a highly reactive monomeric anionic *bis*‐alkyl Al^I^ complex (**VI**), while the group of Kinjo reported a monomeric Al^I^ anion analogue to the CAAC‐carbene ligand.^14^

Some of these anionic Al^I^ complexes are considerably more reactive than (^DIPP^BDI)Al (**II**), which does not react with benzene. Jain et al. calculated that 1,4‐addition of (^DIPP^BDI)Al to benzene, giving the Al^III^ complex (^DIPP^BDI)Al(C_6_H_6_), is only slightly endergonic [Δ*G*(298 K)=+4.8 kcal mol^−1^]^15^ but there is a high transition state.^16^ Our group reported two methods to activate (^DIPP^BDI)Al for reaction with benzene. In combination with a strong “naked” Lewis acid like [(^DIPP^BDI)Ca^+^][B(C_6_F_5_)_4_^−^],^16^ (^DIPP^BDI)Al reacted as a nucleophile to give 1,4‐addition to a preformed π‐benzene/Ca complex (Scheme 1 a).^17^ Or, in cooperation with catalytic quantities of a Lewis base‐free calcium hydride complex^18^ oxidative addition to the benzene C−H bond was observed (Scheme 1 b).^19^ DFT calculations point to a mechanism in which donation of the hydride to the empty *p*‐orbital of Al leads to increased nucleophilicity of the *sp* ^2^ lone pair of Al. The proposed intermediate in this reaction shows parallels to earlier reported anionic Al^I^ complexes (**III**—**VI**). Along similar lines, we now propose a general concept for activation of (^DIPP^BDI)Al by simple addition of a polar *s*‐block metal reagent, R−M, which may generate an anionic Al complex of considerably higher reactivity (Scheme 1 c). This principle is in line with the well‐known reactivity increase of low‐valent late main‐group complexes by neutral donors.^20^, ^21^ While synergy between *s*‐ and *p*‐block metal reagents have led to spectacular deprotonating reagents,^22^ mixing multimetallic cocktails to boost redox activity of low‐valent main‐group metal complexes is rather unexplored.

**Scheme 1:**
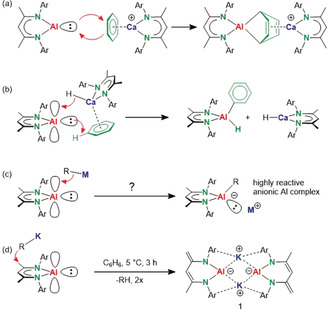
a) Benzene activation by an Al/Ca combination. b) Ca hydride catalyzed C−H bond activation. c) Proposed activation of (^DIPP^BDI)Al by addition of a polar R−M reagent. d) Formation of anionic Al complex **1** by deprotonation.

As all hitherto reported anionic Al complexes contain K^+^ counter cations, we chose to test this concept by addition of a K reagent that is soluble in aromatic solvents. (^DIPP^BDI)Al is unstable in THF.8a Addition of KCH(SiMe_3_)_2_ to an orange solution of (^DIPP^BDI)Al in benzene at 5 °C led, after a few hours, to precipitation of a bright‐yellow powder (**1**, yield: 73 %). NMR data and the crystal structure are conclusive for loss of a proton in the Me group of the ^DIPP^BDI backbone (Scheme 1 d), which implies that the potassium reagent reacted as a base instead as a nucleophile. Repeating the experiment with the significantly weaker base KN(SiMe_3_)_2_ at room temperature also gave **1**. This facile ligand backbone deprotonation is surprising: (^DIPP^BDI)AlMe_2_ does not react with KN(SiMe_3_)_2_ (even not after five days at 80 °C; see Figure S1 in the Supporting Information).

Similar to the anionic Al complexes **III**–**V**, **1** forms a dimer in which formally negatively charged Al centers are bridged by K^+^ ions that show strong K^+^⋅⋅⋅DIPP interactions (Figure 1 a). The ligand in **1** should be regarded as a dianionic *bis*‐amide, [H_2_C=C(NAr)‐C(H)=C(Me)‐NAr]^2−^, and, although disordered, a suitable disorder model shows the typical C−C/C=C bond alternation in the ligand backbone. Compared to **III**–**V**, **1** features the shortest Al–N and Al–K distances, and a considerably smaller N‐Al‐N′ bite angle (Table 1). The shortest Al–K contact of 3.499(1) Å is only slightly longer than the Al–K bond of 3.4549(5) in monomeric **VI** and consequently also the Al⋅⋅⋅Al′ distance of 5.2357(5) Å is significantly shorter than in the other dimers. Therefore, **1** should be considered a tightly bound dimer.

**Figure 1:**
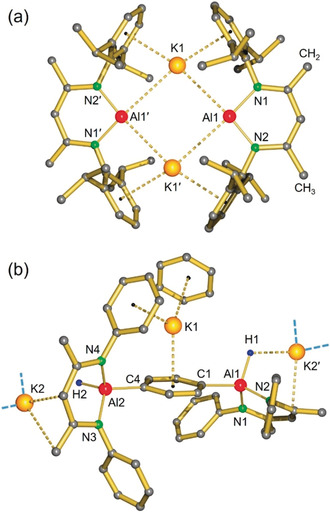
Crystal structures of:^27^ a) complex **1**; see Table 1 for bond distances/angles (the backbone CH_2_ and CH_3_ substituents are disordered and only one of two positions is shown), and b) complex **2**; selected bond lengths in Å: Al–N 1.864(4)–1.878(4), Al–C 1.963(5)–2.007(5), Al–H 1.65(5)–1.66(5), K1⋅⋅⋅C(*para*‐phenylene) 3.085(5)–3.099(5), K2⋅⋅⋅C(BDI) 2.841(9)–2.882(9), K2⋅⋅⋅H 2.47(5), (the *para*‐phenylene unit and K2 are disordered and only distances to the fragment with highest occupation are given).

**Table 1:** Selected bond lengths [Å] and angles [°] for **1** compared to the anionic Al complexes **III**–**VI**.

Complex	**1**	**III**	**IV**	**V**	**VI**
Al‐N	1.865(1)–1.868(1)	1.963(2)–1.956(2)	1.883(2)–1.896(2)	1.887(2)–1.892(2)	2.085(1)^[a]^
Al⋅⋅⋅K	3.499(1)–3.588(1)	3.844(1)–4.070(1)	3.592(1)–3.705(1)	3.584(1)–3.625(1)	3.4549(5)
Al⋅⋅⋅Al′	5.2357(5)	6.627(1)	5.673(1)	5.721(1)	–
K⋅⋅⋅Ar	3.193(1)–3.416(1)	3.226(3)–3.474(3)	3.161(2)–3.563(2)	2.945(1)–3.026(1)	–
N‐Al‐N′	96.05(4)	128.1(1)	105.05(8)	108.8(1)	90.40(5)^[b]^

[a] Al−C bond distance. [b] C‐Al‐C′ bite angle.

The complex **1** dissolves poorly in C_6_D_6_. ^1^H NMR analysis in this solvent reveals two septets and four doublets for the *i*Pr groups, signifying that it retains its highly symmetric dimeric solid‐state structure. This is confirmed by DOSY which gave a MW of 855 Daltons (MW dimer: 966 g/mol). The complex **1** dissolves very well in [D_8_]THF but at 20 °C slowly decomposes. DOSY measurements indicate a MW of 635 Daltons, which fits a monomeric structure solvated by two THF ligands (MW: 627 g/mol).

The reaction of **1** with benzene at 20 °C is very slow, however, heating a bright‐yellow suspension of **1** in C_6_H_6_ to 35 °C gave, after 7 days, a batch of colorless crystals of the product **2**. The crystal structure of **2** shows double C−H bond activation of benzene by oxidative addition exclusively in *para*‐positions (Figure 1 b). The coordination polymer shows two (^DIPP^BDI)AlH units connected by a *para*‐phenylene bridge. While one of the K^+^ ions resides over the phenylene bridge and is embedded in the π systems of DIPP and a co‐crystallized benzene ligand, the second K^+^ ion bridges these fragments with K^+^⋅⋅⋅ligand and K^+^⋅⋅⋅H‐Al contacts. The complex **2** is insoluble in benzene but dissolves in [D_8_]THF. The ^1^H NMR spectrum in this solvent consists of two sets of signals. This can be explained by chirality at the Al centers, chirality which originates from asymmetry in the ligand backbone. Given the large spatial separation of the ligands, the two diastereomers are hardly different in energy and coexist in a 1:1 ratio. At higher temperature fast epimerization of chirality at Al is observed (*T*_coal_=60 °C, Δ*G* ^ǂ^=18.1 kcal mol^−1^). The inversion of chirality at Al likely proceeds by a reversible hydride transfer from Al to K (the short K⋅⋅⋅hydride distance in **2** supports this proposal). The ^1^H NMR signal for the Al‐H units at *δ*=4.2 ppm is extremely broad. Heating **2** in the presence of excess C_6_D_6_ did not result in exchange of the C_6_H_4_^2−^ unit for C_6_D_4_^2−^, which means that its formation is irreversible.

NMR data are in agreement with the exclusive formation of the *para*‐phenylene product. This formation is confirmed by reaction with I_2_ which gave, after work‐up, only *para*‐diiodobenzene (see the Supporting Information). Considering that benzene is present in large excess, the preference for double versus single C−H bond activation is intriguing. Formation of **2** may be explained by a template effect that shows parallels to Mulvey's mixed‐metal base chemistry.22a, 22b Synergy in these heterobimetallic systems enable challenging multiple deprotonations, selectively giving highly charged species. As in Mulvey's “inverse crown ethers”, the metals in the Al/K complexes **III**–**V** and **1** are also arranged in a ring. Similar to the multiply charged anions in inverse crown ether complexes, the C_6_H_4_^2−^ ion in **2** is also stabilized by several metal contacts. The importance of K^+^ for the reactivity of **2** was investigated by incorporation of this cation in [2.2.2]cryptand. While addition of this cryptand to **III** changed the course of the reaction from C−H to C−C bond cleavage,^11^ addition to **1** led to single C−H bond activation. This reactivity was confirmed by quenching the reaction mixture with I_2_ which gave, after work‐up only, iodobenzene instead of *para*‐diiodobenzene (see the Supporting Information).

DFT calculations (ωB97XD/6–311+G**//ωB97XD/6–31+G**) gave further insight into the formation and electronic structure of **1**. Although we never isolated our target complex [(^DIPP^BDI)AlN(SiMe_3_)_2_]^−^K^+^, calculations show that its formation is slightly exergonic (Scheme 2 a). In agreement with experiment, the barrier for subsequent intramolecular backbone deprotonation is low. Since BDI backbone deprotonation is generally not easy, prior formation of intermediate [(^DIPP^BDI)AlN(SiMe_3_)_2_]^−^K^+^ may be the key to this process. Consistent with this reactivity is the observation that addition of neutral Lewis bases to (^DIPP^BDI)Al also led to proton loss of the backbone Me group.20a, 20b, 20c, 20d, 21 The anion in **1** is isolobal to neutral silylene and germylene complexes with the same deprotonated ^DIPP^BDI ligand.^23^

**Scheme 2:**
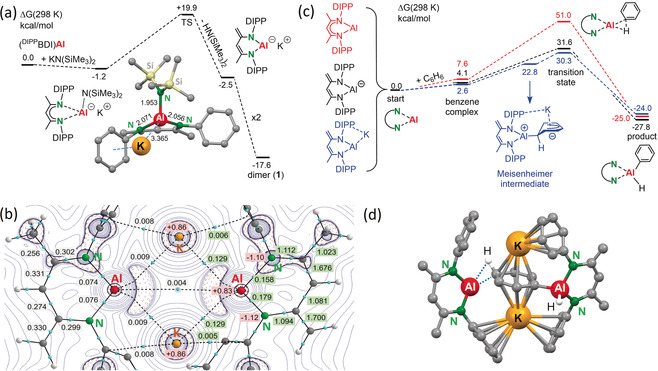
DFT calculations (ωB97XD/6–311+G**//ωB97XD/6–31+G**, PCM=benzene). a) Energy profile for reaction of (^DIPP^BDI)Al with KN(SiMe_3_)_2_. b) Electron distribution by AIM given the electron densities *ρ*(*r*) in the bcp's, NPA charges (red boxes), and Wiberg bond indexes (green boxes). c) Comparison of the energy profiles for benzene C−H bond activation by (^DIPP^BDI)Al (red), the anion in **1** (black) or its K‐complex (blue). d) The transition state for the second C−H bond activation in benzene by a dimeric model system of **1** (DIPP replaced by Ph).

The calculated geometry of **1** compares well to that of the crystal structure (see Figure S30). Atoms‐in‐molecules (AIM) analysis shows the high electron densities at the Al centers which combined with a short Al⋅⋅⋅Al distance gives rise to a bond‐critical‐point (bcp), but the electron density of 0.004 *e* is rather low (Scheme 2 b). The bcp electron densities and Wiberg bond indices in the ligand backbone are consistent with C−C/C=C bond alternation. Low electron densities at the Al⋅⋅⋅K bcp's are in agreement with highly ionic contacts. The HOMO energy of the full dimeric complex **1** lies at −4.290 eV (B3LYP/6–31+G*, Figure S31) and fits previously calculated values at this level: −4.035 eV (**III**), −4.205 eV (**IV**), and −3.630 eV (**VI**).14a Thus, the nucleophilic character of **1** should be similar to that of **IV** but lower than that of either **III** or **VI**. Compared to (^DIPP^BDI)Al (HOMO at B3LYP/6–31+G*: −4.458 eV), the reactivity of **1** should be clearly higher. This reactivity is illustrated by comparison of the calculated energy profiles for benzene C−H activation by (^DIPP^BDI)Al and the anion in **1** (Scheme 2 c). Formation of a loosely bound benzene complex is for both slightly endergonic (+7.6/+4.1 kcal mol^−1^) while product formation is similarly exergonic (−27.8/−25.0 kcal mol^−1^). The main difference between the energy profiles lies in the activation barriers for C−H activation. Although these Δ*G* values are quite high because of overestimation of entropic factors,^24^ the barrier for the anionic Al species (+27.5 kcal mol^−1^) is much lower than for (^DIPP^BDI)Al (+43.4 kcal mol^−1^). The activation free energy for benzene C−H bond cleavage by the anion in **1** compares well to that calculated for the anion in **III** (+28.7 kcal mol^−1^ 11 or +31.8 kcal mol^−1^ 25). Incorporation of countercation K^+^ led to a slightly lower activation free energy of +26.4 kcal mol^−1^. Interestingly, in the presence of K^+^ an intermediate reminiscent of a Meisenheimer anion was found. The species originates from nucleophilic attack of Al^I^ at benzene resulting in a strongly buckled aromatic ring. NPA charges show that Al (+2.10) is formally +III while the ring (−1.53) carries a high negative charge which is stabilized by K^+^ (+0.96). As has been noted earlier,^11^, ^12^ the presence or absence of K^+^ can clearly change the course of anionic Al reactivity and should be considered in modelling its reactions. Most recently, a similar Al/K cooperativity was observed in the reaction of an aluminoxane potassium salt with CO_2_.^26^

DFT calculations may also shed light on the *para*‐selective double C−H bond activation. Calculations on a simple model system like [H_3_Al‐C_6_H_4_‐AlH_3_]^2−^ confirm a thermodynamic preference. Relative Δ*G* (kcal mol^−1^): 0.0 (*para*), +3.5 (*meta*), +14.0 (*ortho*). However, single alumination would thermodynamically still be preferred: [H_3_Al‐C_6_H_4_‐AlH_3_]^2−^ + C_6_H_6_ → 2 [H_3_Al‐C_6_H_5_]^−^ is exergonic by Δ*G*=−28.0 kcal mol^−1^. Also the transition state for *para*‐alumination of [H_3_Al‐C_6_H_5_]^−^ is more than 10 kcal mol^−1^ higher than that for mono‐alumination of C_6_H_6_ (see Figures S38 and S42). Given the important role of K^+^ in the mechanism, preference for double alumination may be explained by a template effect. This effect is supported by calculations on a full dimeric model system in which the DIPP groups in **1** were replaced by Ph. The activation barriers for the first alumination (Δ*G*=+31.8 kcal mol^−1^) and the challenging second alumination (Δ*G*=+34.3 kcal mol^−1^) are high but of similar magnitude (see Figure S44). Reminiscent of “inverse crown ethers”, the second transition state displays multiple metal–Ph contacts (Scheme 2 d). The Ph ring is sandwiched between K^+^ ions that help stabilize its transformation into C_6_H_4_^2−^, again underscoring the importance of K^+^ cations for the reactivity of **1**.

In summary, the reactivity of Roesky's well‐known Al^I^ complex (^DIPP^BDI)Al (**II**), which is kinetically stabilized by a chelating β‐diketiminate ligand, is easily boosted by the addition of a simple potassium base. Although we anticipated formation of the aluminate complex [(^DIPP^BDI)RAl^−^]K^+^, deprotonation of a backbone Me group led to a dimeric anionic Al^I^ complex (**1**). This complex, which dissolved in benzene is also dimeric, carries considerable electron density on Al, and is highly reactive. It activates two C−H bonds in benzene by oxidative addition giving exclusively the *para*‐aluminated product which in reaction with iodine forms *para*‐diiodobenzene. Encapsulation of the K^+^ ions in **1** by a cryptand changed its reactivity and at most single C−H bond activation in benzene was observed. Calculations support the idea that the reactivity of **1** is determined by close cooperation of Al^I^ and K^+^ centers. We will continue to study synergistic effects between covalent low‐valent *p*‐block metal species and ionic *s*‐block metal reagents.

## Conflict of interest

The authors declare no conflict of interest.

## Supporting information

As a service to our authors and readers, this journal provides supporting information supplied by the authors. Such materials are peer reviewed and may be re‐organized for online delivery, but are not copy‐edited or typeset. Technical support issues arising from supporting information (other than missing files) should be addressed to the authors.

Supplementary
