# Supplementary material for: Boosting Low‐Valent Aluminum(I) Reactivity with a Potassium Reagent
Source: Angew Chem Int Ed Engl. 2020 Jun 29;59(37):15982–6. doi: 10.1002/anie.202006693 (PMC7540686; doi:10.1002/anie.202006693)
Supplement: Supplementary file 1 — Supplementary [file ANIE-59-15982-s001.pdf]

Angewandte Chemie
Eine Zeitschrift der Gesellschaft Deutscher Chemiker

Supporting Information

Boosting Low-Valent Aluminum(I) Reactivity with a Potassium Reagent

*Samuel Grams, Jonathan Eyselein, Jens Langer, Christian Färber, and Sjoerd Harder**

anie_202006693_sm_miscellaneous_information.pdf

Supporting Information

Table of Contents

1. Synthetic procedures

1.1. General remarks……S2

1.2. Complex syntheses and reactivity studies……S3

2. NMR spectroscopy

2.1. NMR spectra of complexes 1 & 2……S7

2.2. Temperature dependent ^{1}H NMR of complex 2……S17

2.3. Diffusion measurements (DOSY) of complex 1……S20

3. Computational Details

3.1. General methods……S23

3.2 Summary of results……S23

3.3. XYZ-files……S39

4. Single crystal X-ray diffraction

4.1. Structure determination of complex 1……S91

4.2. Structure determination of complex 2……S91

5. References……S94

1. Synthetic procedures

1.1. General remarks

All experiments were carried out under an inert nitrogen atmosphere in a glove box (MBraun, Labmaster SP) or by using standard *Schlenk* techniques. Benzene, THF, diethyl ether, *n*-pentane, hexanes and toluene were degassed with nitrogen, dried over activated aluminum oxide (Innovative Technology, Pure Solv 400-4-MD, Solvent Purification System) and stored under inert atmosphere over molecular sieves (3Å). *m*-Xylene was degassed with nitrogen, refluxed on CaH_2 for 24 h, distilled under nitrogen atmosphere stored over molecular sieves (3Å). Deuterated benzene (C_6D_6; 99.6+% D), toluene-d_8 (99.6+% D), THF-d_8 (99.5+% D), $CDCl_3$ (99.8+% D) were purchased from Deutero GmbH or Sigma-Aldrich and stored over molecular sieves (3Å). $KN(SiMe_3)_2$ (95 %) was purchased from Sigma-Aldrich and used as is. The [2.2.2]cryptand 4,7,13,16,21,24-hexaoxa-1,10-diazabicyclo[8.8.8]hexacosane (98 %) was purchased from TCI and used as is. ^{DIPP}BDI-H,[S1] (^{DIPP}BDI)Al[S2] and $KCH(SiMe_3)_2$[S3] were synthesized according to a literature procedure.

Multinuclear NMR spectra were recorded on a Bruker Avance III HD 400 MHz or 600 MHz spectrometer at ambient temperature (variable temperature spectra were recorded at the given temperature). Chemical shifts δ are reported in *parts per million* (ppm) relative to Me_4Si as external standard and referenced internally to the carbon nuclei ($^{13}C\{^1H\}$) or residual protio solvent resonances (^1H) of the deuterated solvents. Assignments of resonance signals in the ^1H and $^{13}C\{^1H\}$ NMR spectra were made based on two dimensional NMR correlation (HSQC, HMBC, COSY) and ATP experiments. Coupling constants *J* are given in Hertz (Hz). Signal multiplicities are abbreviated as follows: s (singlet), d (doublet), sept (septet), m (multiplet) and br (broad). Elemental analyses were obtained on an Euro Vector EA3000 Elemental Analyzer.

All crystal structures have been measured on a SuperNova (Agilent) diffractometer with dual Cu and Mo microfocus sources and an Atlas S2 detector. GC/MS measurements were performed on a Thermo Scientific™ Trace™ 1310 gas chromatography system (carrier gas Helium) with detection by a Thermo Scientific™ ISQ™ LT Single Quadrupole mass spectrometer. A Phenomenex® ZebronTMZB-5 column of the dimensions 0.25mm x 30m with a film thickness of 0.25µm was used. The samples (1µl) were injected with an Instant Connect-SSL Module in the split mode (Injector Temperature: 280 °C). Temperature programs were started at 40 °C followed by heating ramps, optimized for the separation problem, until 280°. Baseline separation of each analyte was achieved by choosing different temperature programs. The molecular identity was confirmed by comparison with entries in the NIST/EPA/NIH mass spectral library (version 2.2, built June 10 2014).

1.2. Complex syntheses and reactivity studies

Synthesis of complex 1. (^{DIPP}BDI)Al (**II**) (211 mg, 475 µmol) and $KCH(SiMe_3)_2$ (95 mg, 479 µmol) were weighed into a Schlenk flask. After cooling to 5 °C, 3 ml of benzene were added and the suspension was stirred till the orange color of (^{DIPP}BDI)Al had vanished (approximately 3-4 h). Since the anionic aluminium product reacts with Teflon, magnetic stirring was performed with a glass-coated stirring bar. All solids were collected on a frit, washed with 0.5 ml of benzene and dried thoroughly in high vacuum to give **1** (166.7 mg, 345 µmol, 73% yield) as a yellow powder. Crystals suitable for X-ray diffraction analysis were obtained by recrystallization from *m*-xylene *via* solvent diffusion with hexanes at –30 °C. The synthesis of **1** can also be achieved by replacing $KCH(SiMe_3)_2$ with the weaker base $KN(SiMe_3)_2$, however, the yields are lower (circa 30%) and the product is contaminated with $KN(SiMe_3)_2$. Therefore, deprotonation with $KCH(SiMe_3)_2$ is the preferred synthetic route.

Elemental analysis (%): calculated: C 72.16, H 8.35, N 5.80; found: C 72.16, H 8.56, N 5.64.

^{1}H NMR (600 MHz, THF-d_8, 25 °C): δ =7.03+6.99 (4H, *m*-H DIPP), 6.94 (m, 2H, *p*-H DIPP), 4.69 (s, 1H, backbone CH), 3.74 (two overlapping septets, 4H, *i*Pr-CH), 2.90+2.09 (two broad singlets, 2H, CH_2), 1.36 (s, 3H, backbone CH_3) 1.23 (d overlapping, $^3J_{HH}$, 24H, *i*Pr-CH_3).

^{13}C{^{1}H} NMR (151 MHz, THF-d_8, 25 °C): δ = 156.1 (**C**=CH_2), 148.7 - 148.1 (3 signals, Ph**C**), 142.7 (CH=(N)**C**–CH_3), 124.6 – 123.0 (4 signals, Ph**C**H), 102.6 (backbone **C**H), 74.7 (**C**H_2), 28.7 & 28.6 (*i*Pr**C**H_3), 26.4 - 25.9 (overlapping with THF-d_8, *i*Pr**C**H_3), 24.6 & 24.3 ((N)C-**C**H_3).

No resonances were found in ^{27}Al NMR within the range of -560 to +560 ppm

^{1}H NMR (600 MHz, C_6D_6, 25 °C): δ = 6.98 (d, $^3J_{HH}$ = 7.4 Hz, 2H, *m*-PhH), 6.93 - 6.90 (m, 1H, *p*-PhH), 6.85 - 6.82 (m, 3H, DIPP-PhH), 5.36 (s, 1H, backbone CH) 3.92 (s, 1H, CH_2), 3.89 (sept, overlapping, $^3J_{HH}$, 2H, *i*PrCH), 3.72 (sept, $^3J_{HH}$ = 6.2 Hz, 2H, *i*PrCH), 2.98 (s, 1H, CH_2), 1.64 (s, 3H, backbone CH_3), 1.36 (d, $^3J_{HH}$ = 6.8 Hz, 3H, *i*PrCH_3), 1.13 - 1.11 (two d, overlapping, $^3J_{HH}$, 6H, *i*PrCH_3), 1.08 (d, $^3J_{HH}$ = 6.9 Hz, 3H, *i*PrCH_3).

^{13}C{^{1}H} NMR (151 MHz, C_6D_6, 25 °C): δ = 154.1 (**C**=CH_2), 150.3 & 149.7 (Ph**C**-*i*Pr), 147.8 & 147.6 (Ph**C**-N), 142.1 (CH=(N)**C**–CH_3), 125.18 - 122.9 (4 signals, Ph**C**H), 102.5 (backbone **C**H), 77.6 (**C**H2), 28.1 - 23.41 (*i*Pr**C**H_3 and backbone **C**H_3).

Reaction of 1 with C_6D_6 in presence of [2.2.2]cryptand. In a J. Young tube **1** (8.7 mg, 18 µmol) and [2.2.2]cryptand 4,7,13,16,21,24-hexaoxa-1,10-diazabicyclo[8.8.8]hexacosane (6.9 mg, 18 µmol) were combined with 1 ml of C_6D_6. Upon shaking, all solids dissolved quickly (< 1 min) and a clathrate was formed (liquid-liquid phase sepatration). The J. Young tube was stirred and heated to 65 C° for 24 h. After letting the tube cool to room temperature the supernatant C_6D_6 was decanted of. The clathrate was dissolved in

THF-d_8. Iodine (9.5 mg, 75 µmol) was added and the reaction mixture was heated to 80 °C for 24 hours. The reaction mixture was exposed to air to hydrolyze remaining reactive species followed by a filtration to removed solid residues. The filtrate was analyzed by GC/MS methods. Iodobenzene-d_5 (R_T: 6.01) was identified by its mass spectrum and fragmentation as well as retention time which was compared to an original sample of iodobenzene (R_T: 6.03). No traces of $C_6D_4I_2$ could be detected.
C_6D_5I: GC-MS (EI, 70 eV), R_T 6.01 min: m/z (%) = 209.0 (33) [M+], 82.1 (100) [C_6D_5+].

For comparison, reaction of **1** with benzene without added cryptand and subsequent quenching with I_2 gave only *para*-diiodobenzene.

Synthesis of complex 2. Complex **1** (11 mg, 23 µmol) was suspended in 500 µL of benzene and placed in an oil bath of 35 °C for 6 days. Occurrence of a reaction is indicated by the disappearance of the yellow powder of **1** and formation of colorless crystals of **2**. Traces of starting materials and minor impurities were removed by washing with benzene until the washing solution was colorless. Crystals were partially dried in the N_2 atmosphere of a glovebox but removal of all solvent under high vacuum led to decomposition. This precludes accurate elemental analysis. The washed crystals could be directly used for X-ray diffraction studies and were dissolved in THF-d_8 for NMR spectroscopy investigations. Due to chirality at both Al centers, a double set of signals was observed for the two diastereomers in a 1/1 ratio.

^{1}H NMR (600 MHz, THF-d_8, 25 °C): δ = 7.30 (s, 10 H, coordinated/excess C_6H_6), 6.94 - 6.75 (m, 12 H, DIPP), 6.53+6.52 (two s, 4H, C_6H_4), 4.73+4.72 (two s, 2H, backbone CH), 4.21 (broad, 2H, AlH), 3.91-3.62 (overlapping septets, 8H, *i*PrCH), 2.98 (d, $^2J_{HH}$ = 2.2 Hz, 1H, CH_2), 2.97 (d, $^2J_{HH}$ = 2.3 Hz, 1H, CH_2) 2.18 (d, $^2J_{HH}$ = 2.3 Hz, 1H, CH_2), 2.14 (d, $^2J_{HH}$ = 2.2 Hz, 1H, CH_2), 1.45+1.43 (two s, 6H, backbone CH_3), 1.21 – 0.50 (16 doublets some overlapping, $^3J_{HH}$ = 6.9 Hz, 48 H, *i*PrCH3).
^{13}C{^{1}H} NMR (151 MHz, THF-d_8, 25 °C): δ = 158.53 & 158.39 (H_2C=**C**(N)-C(H)), 149.7 – 146.7 (14 signals, Ph**C** and Al-Ph**C**), 137.71 (Al-Ph**C**H), 128.7 (t, *J* = 24.15 Hz, benzene), 124.4 – 123.1 (12 signals, Ph**C**H), 100.3 & 99.9 (backbone **C**H), 74.8 & 74.6 (**C**H_2), 29.0 – 24.2 (overlapped by THF-d_8. C=(N)C-**C**H_3, *i*Pr**C**H_3 and *i*Pr**C**H).

Reaction of 2 with I_2. In a J. Young tube containing a solution of **2** (14 mg of partially dried crystals, approximately 12 µmol) in THF-d_8 I_2 (3 mg, 12 µmol) was added. The reaction was accompanied by gas (H_2) evolution. Additional I_2 (4 mg, 16 µmol) was added and the reaction mixture was heated to 80 °C for 20 h during which time the color changed from purple to brown. The reaction mixture was exposed to air to

hydrolyze remaining reactive species and this was followed by evaporation of all volatiles under reduced pressure. The obtained crude product was suspended in diethyl ether and all solids were filtered off. The filtrate was analyzed by GC/MS measurements. These measurements proved that the sample contained only one compound with a mass for $C_6H_4I_2$. Remaining filtrates were combined and the diethyl ether was evaporated under reduced pressure. The product $C_6H_4I_2$ was extracted with hexanes, dried, resublimed and dissolved in $CDCl_3$ for NMR analysis. The chemical shifts are perfectly consistent with literature values for *para*-diiodobenzene.[(S4)]

$C_6H_4I_2$, GC-MS (EI, 70 eV), R_T 9.39 min: m/z (%) = 329.9 (51) [M+], 76.05 (100).

^{1}H NMR (400 MHz, $CDCl_3$, 25 °C): δ = 7.41 (s, 4 H).

Reaction of (^{DIPP}BDI)AlMe$_2$ with KN(SiMe$_3$)$_2$. (^{DIPP}BDI)Al(Me)$_2$ (24 mg, 51 µmol) and KN(SiMe$_3$)$_2$ (10 mg, 50 µmol) were combined in a J. Young tube and dissolved by 600 µl C_6D_6. After heating the reaction mixture to 80 °C for 5 days, the tube was cooled to room temperature. Subsequent ^{1}H NMR experiments showed that, apart from minor decomposition, the backbone Me group was not deprotonated.

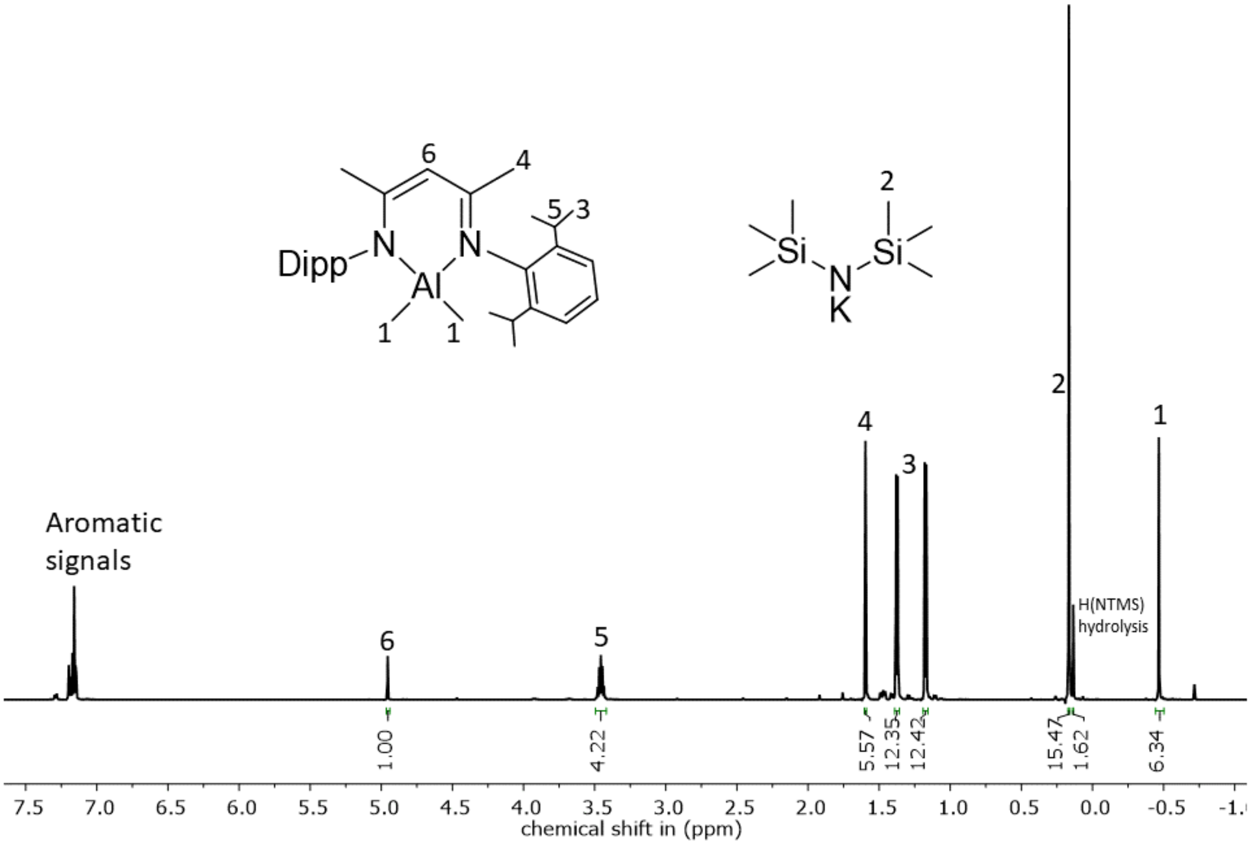

Figure S1: ^{1}H NMR spectrum of the reaction mixture of (^{DIPP}BDI)AlMe$_2$ and KN(SiMe$_3$)$_2$ after 5 days of heating to 80 °C. C_6D_6, 600 MHz, 298 K.

2. NMR Spectroscopy

2.1. NMR spectra of complexes 1 & 2

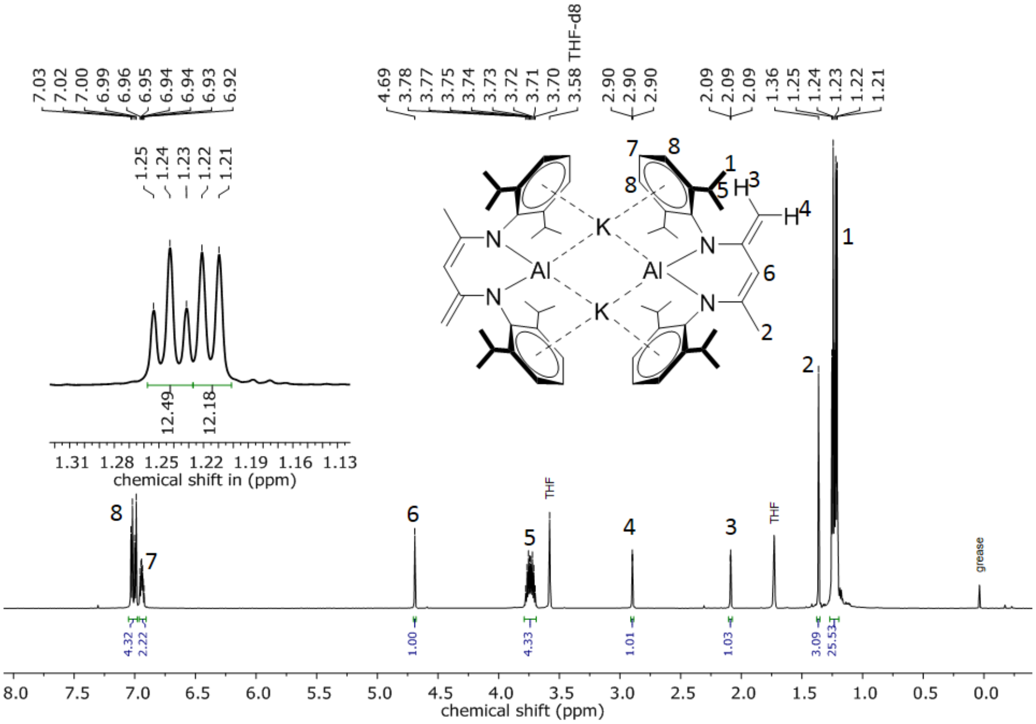

Figure S2: ^{1}H NMR spectrum of **1**. 600 MHz, THF-d_8, 25 °C

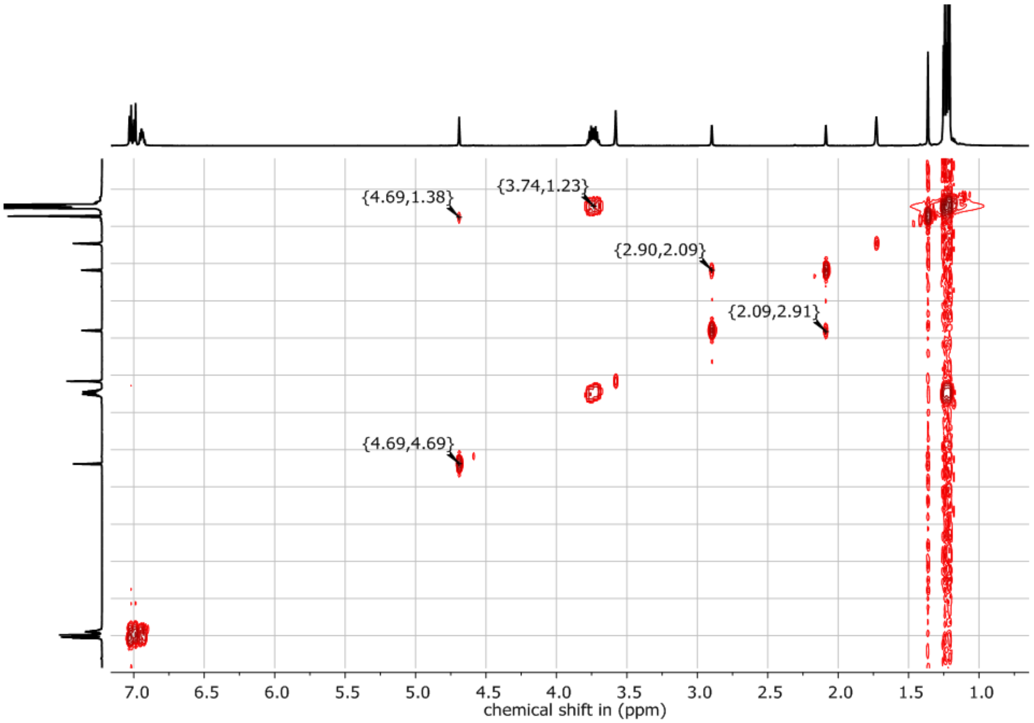

Figure S3: COSY NMR of **1**. 600 MHz, THF-d_8, 25 °C

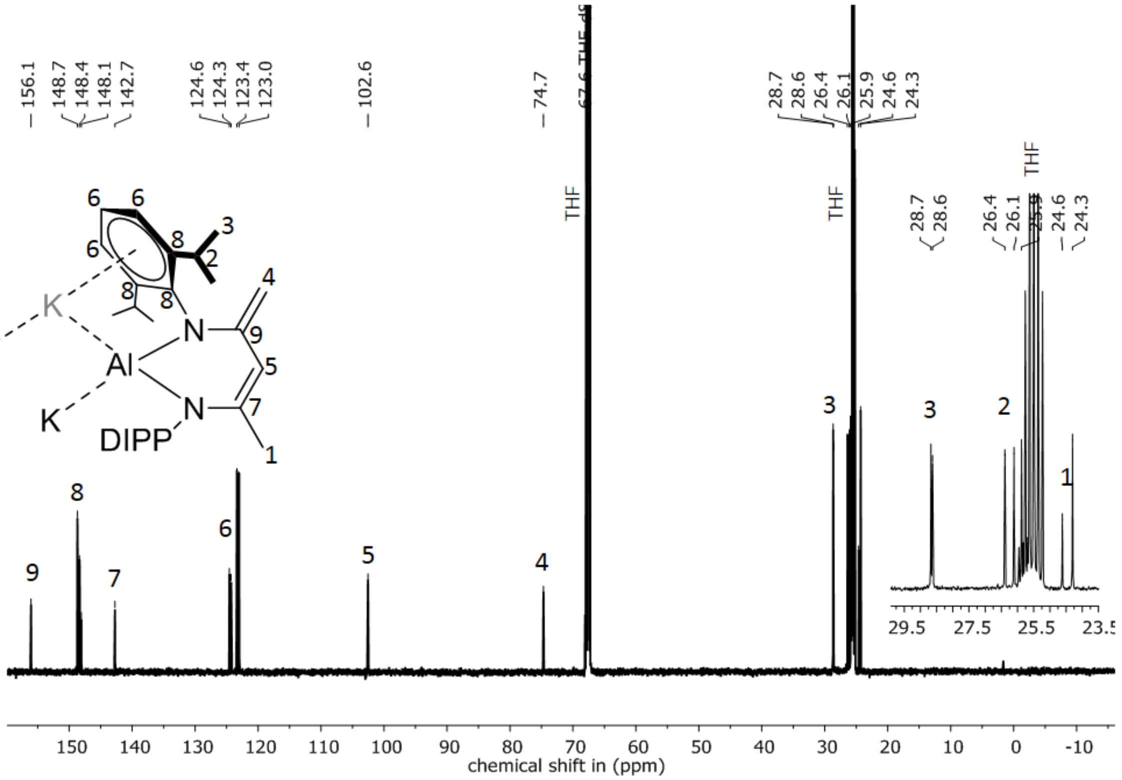

Figure S4: $^{13}C\{^1H\}$ NMR spectrum of **1**. 151 MHz, THF-d_8, 25 °C

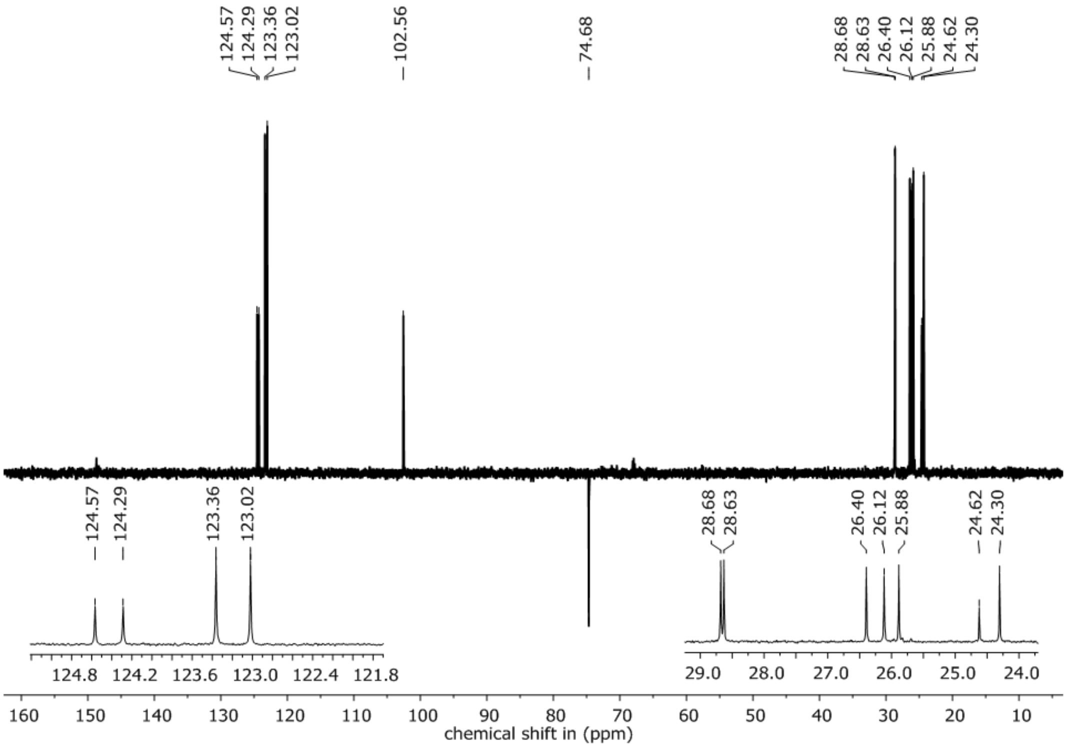

Figure S5: DEPT-135 NMR spectrum of **1**. 151 MHz, THF-d_8, 25 °C

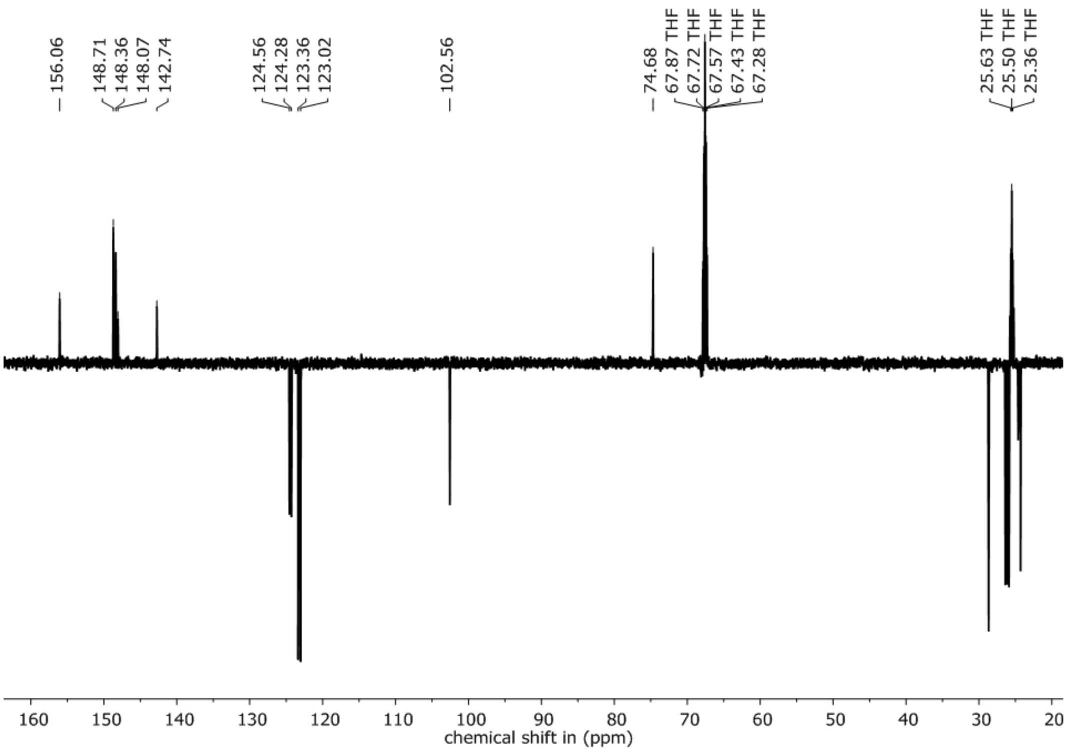

Figure S6: Attached Proton Test of **1**. 151 MHz, THF-d_8, 25 °C.

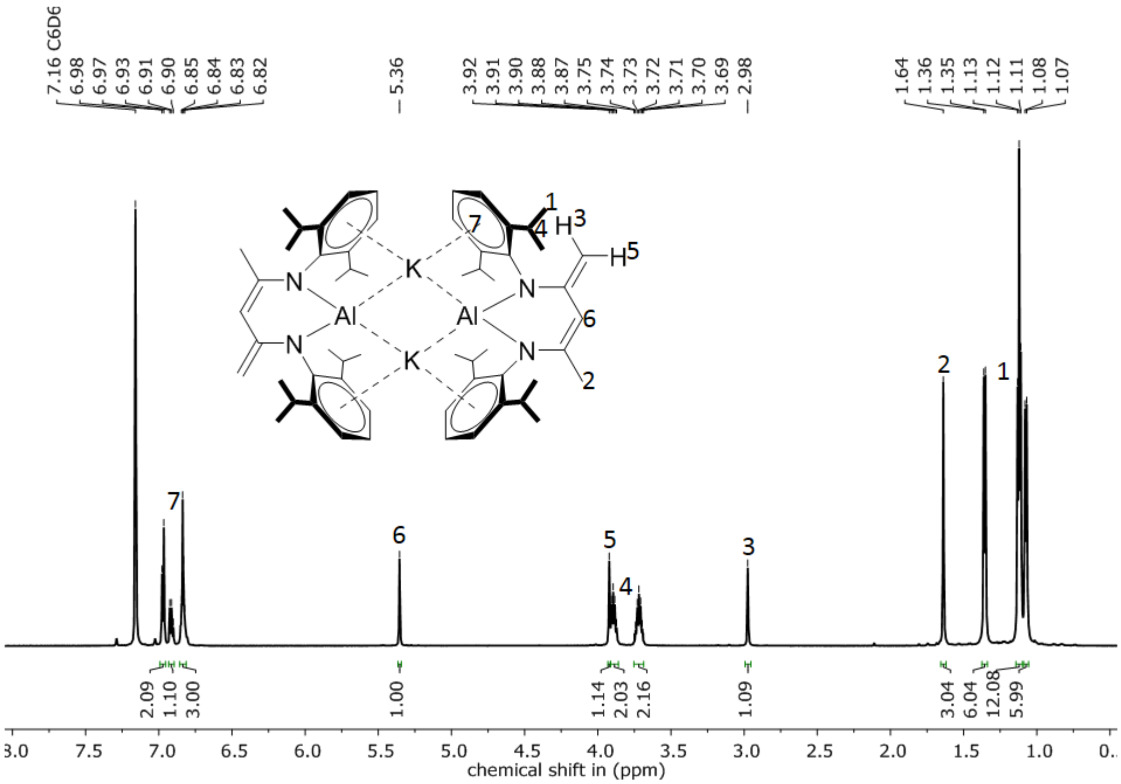

Figure S7: ^{1}H NMR spectrum of **1**. 600 MHz, C_6D_6, 25 °C.

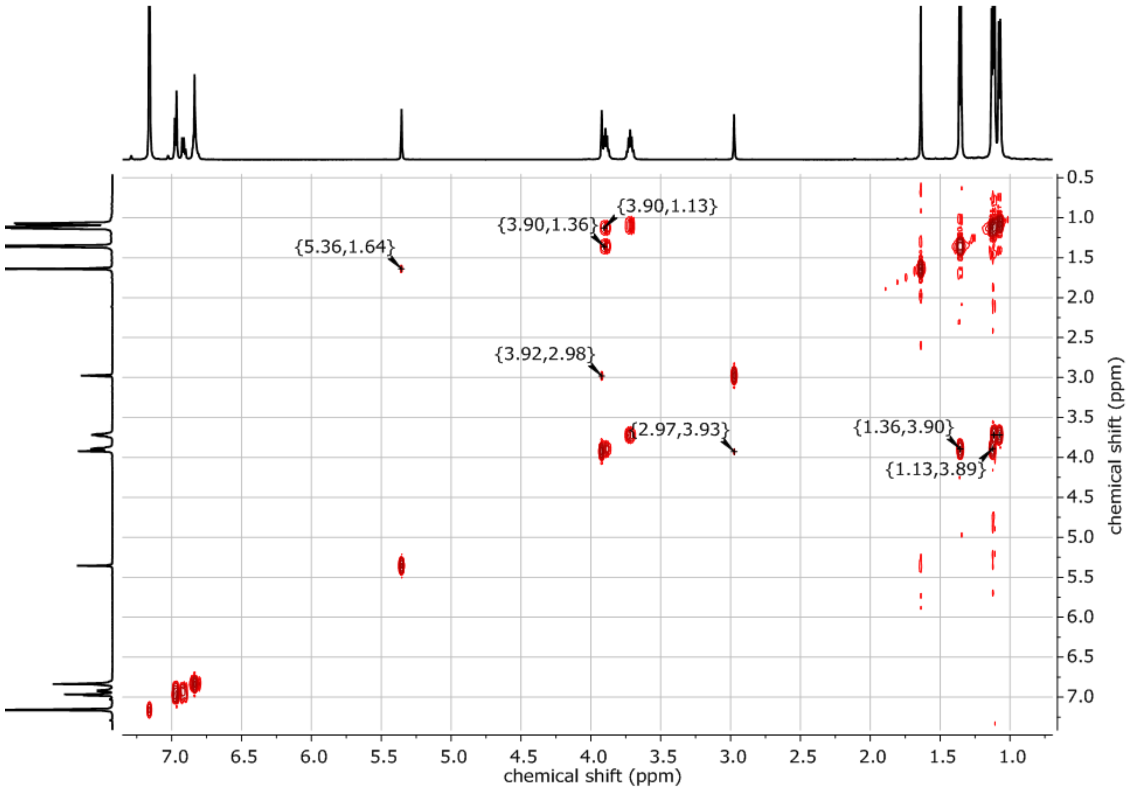

Figure S8: COSY NMR spectrum of **1**. 600 MHz, C_6D_6, 25 °C.

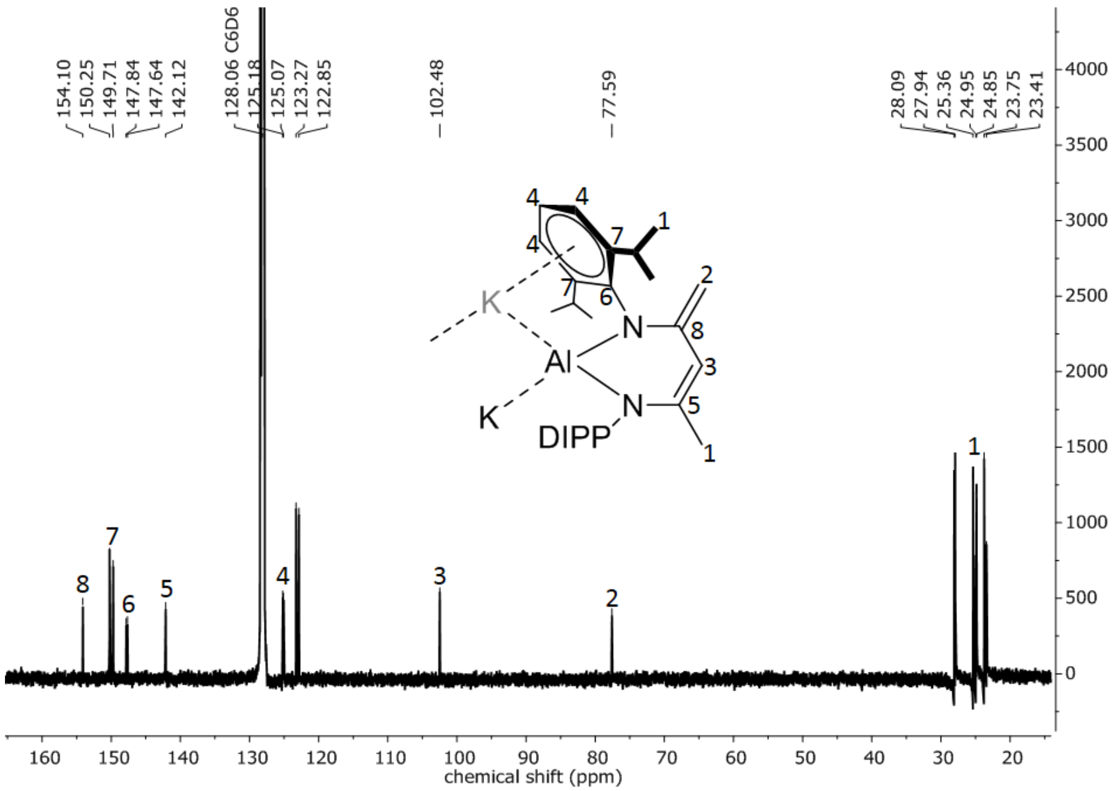

Figure S9: $^{13}C\{^{1}H\}$ NMR spectrum of **1**. 151 MHz, C_6D_6, 25 °C.

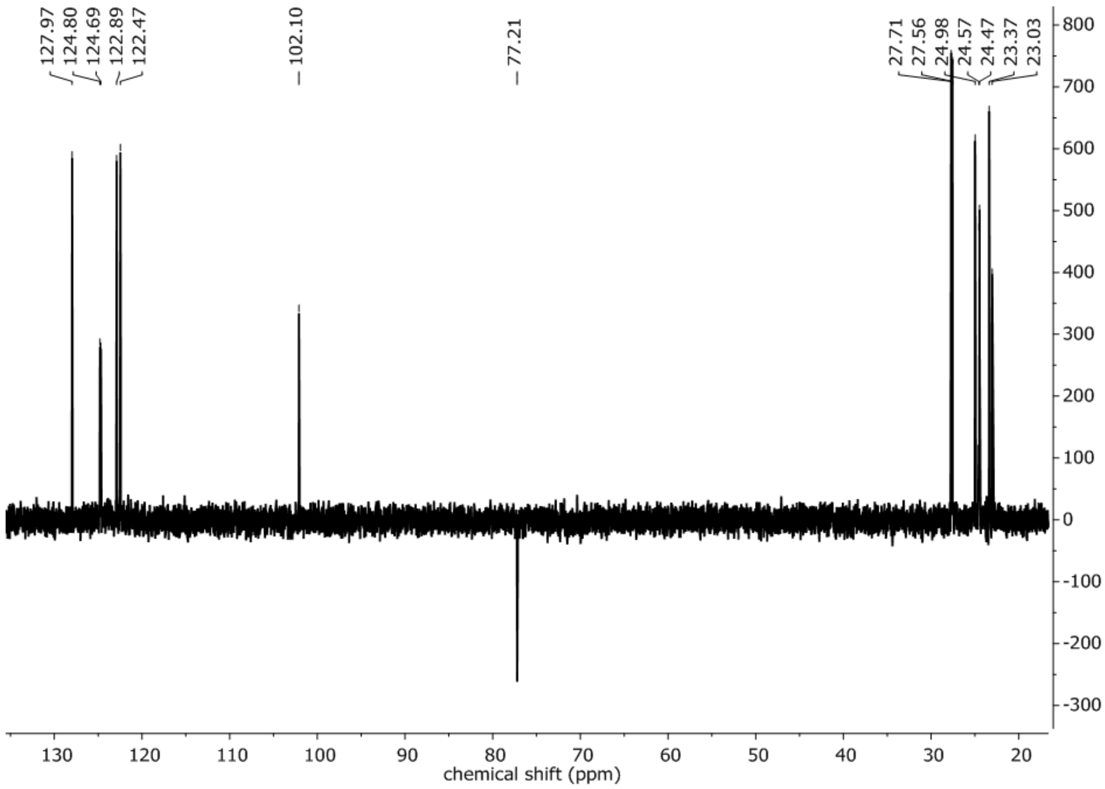

Figure S10: DEPT-135 NMR spectrum of **1**. 151 MHz, C_6D_6, 25 °C.

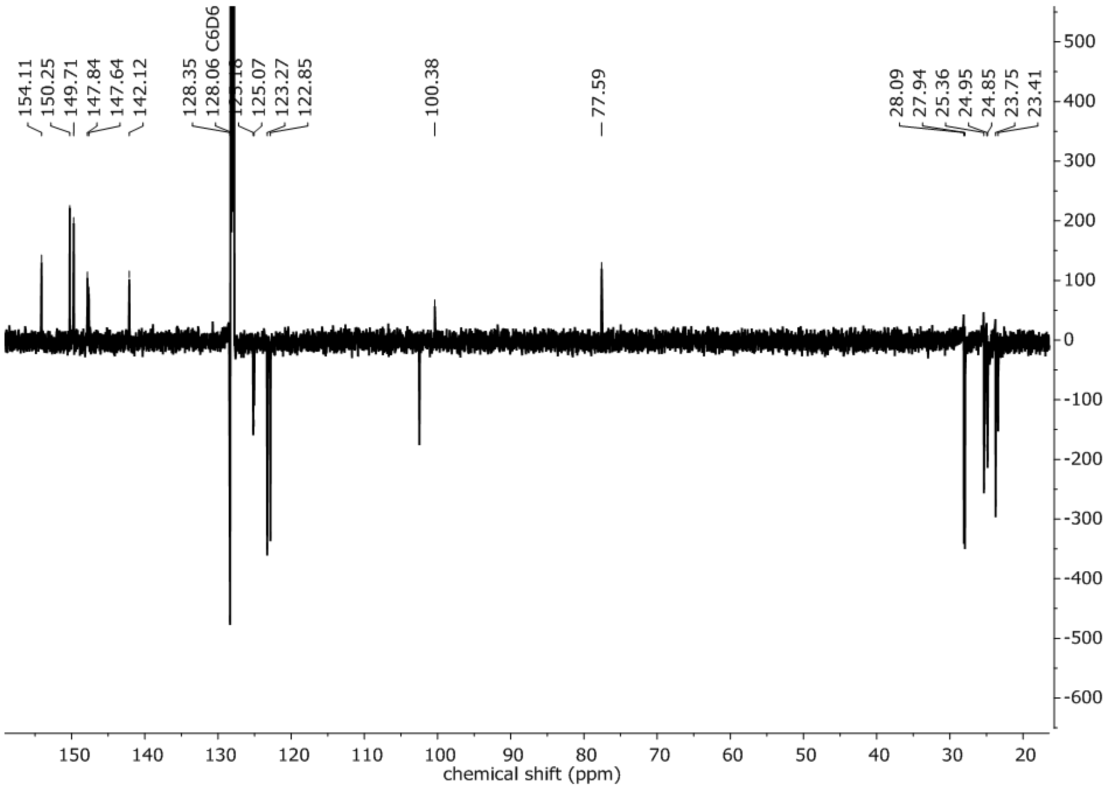

Figure S1: Attached Proton Test of **1**. 151 MHz, C_6D_6, 25 °C

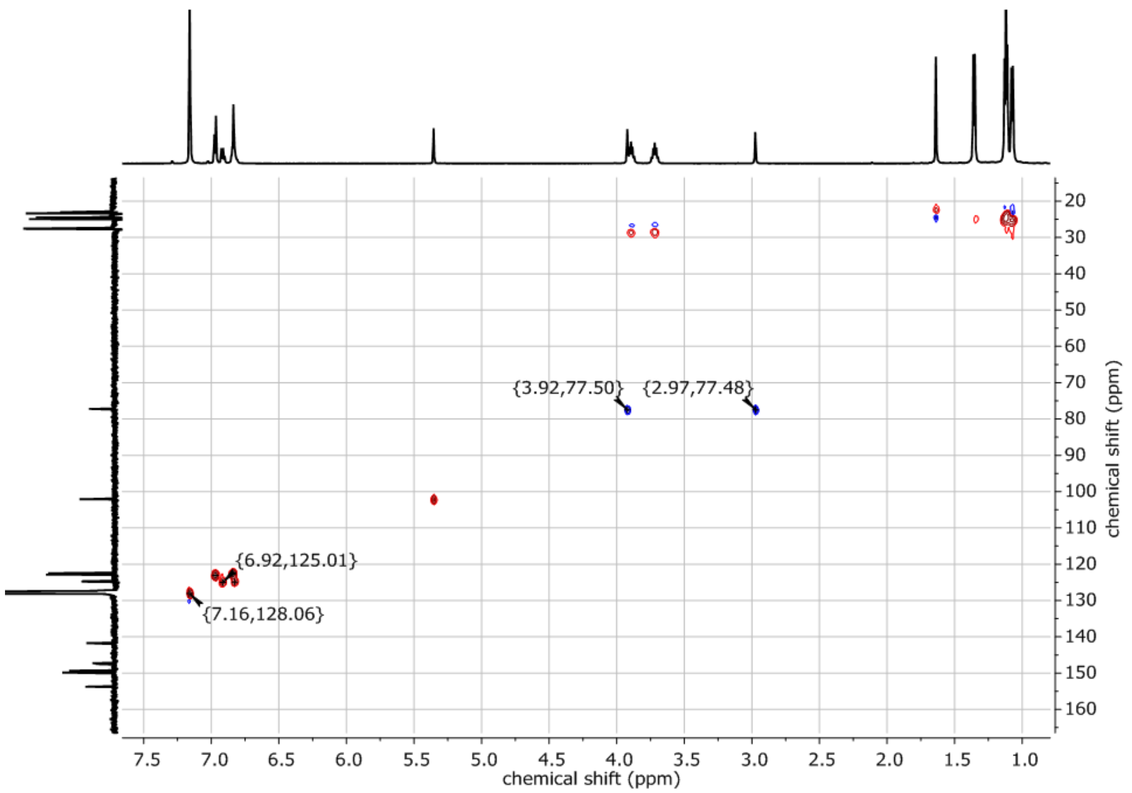

Figure S12: HSQC NMR spectrum of **1**. C_6D_6, 25 °C.

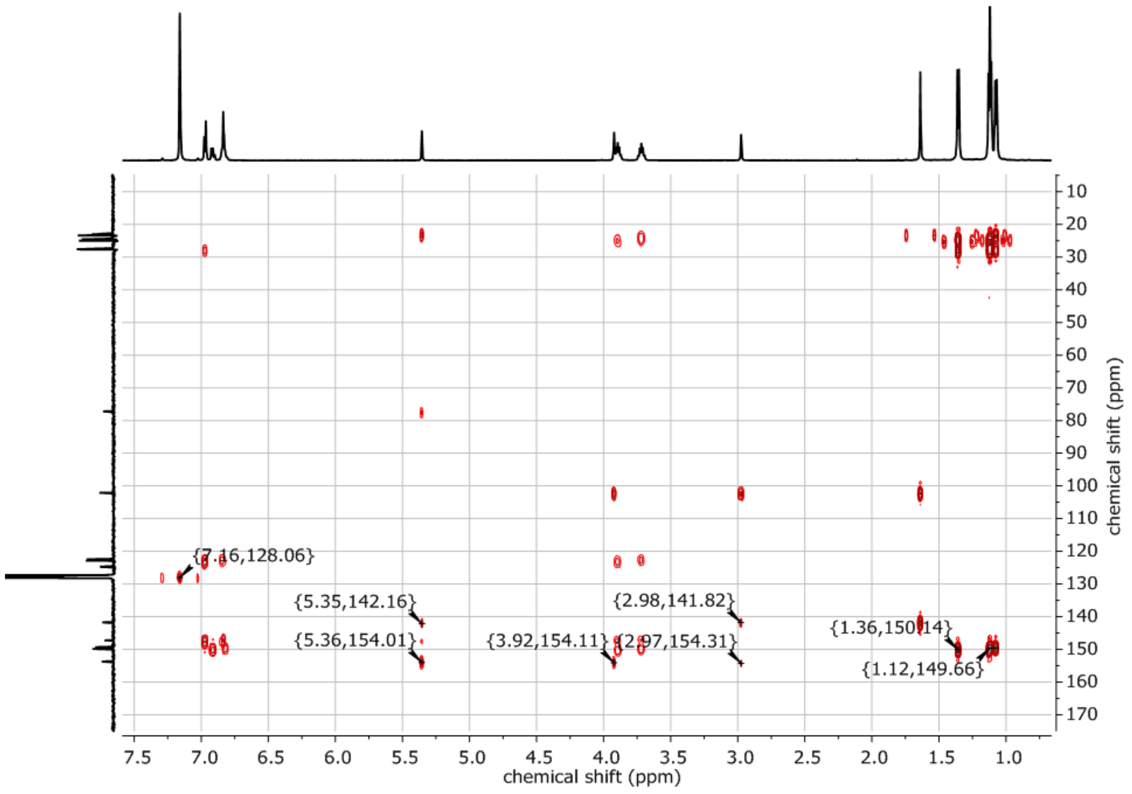

Figure S13: HMBC NMR spectrum of **1**. C_6D_6, 25°C.

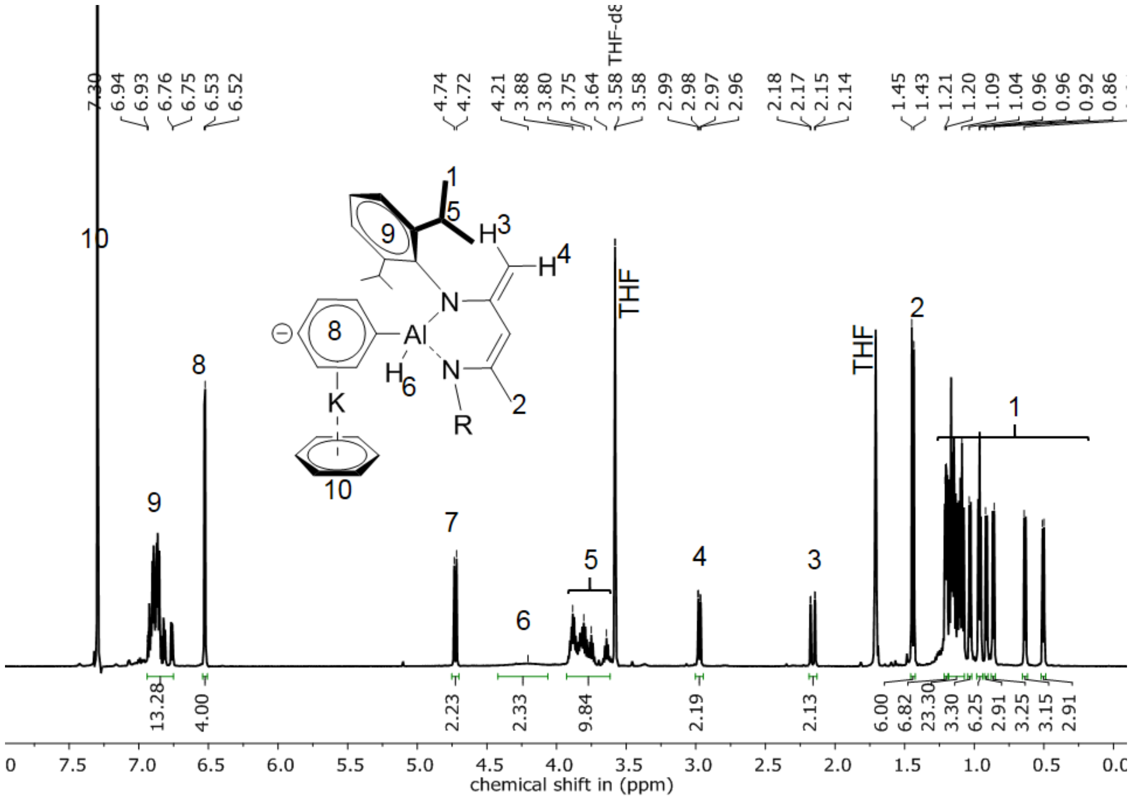

Figure S14: ^{1}H NMR spectrum of **2**. 600 MHz, THF-d_8, 25 °C.

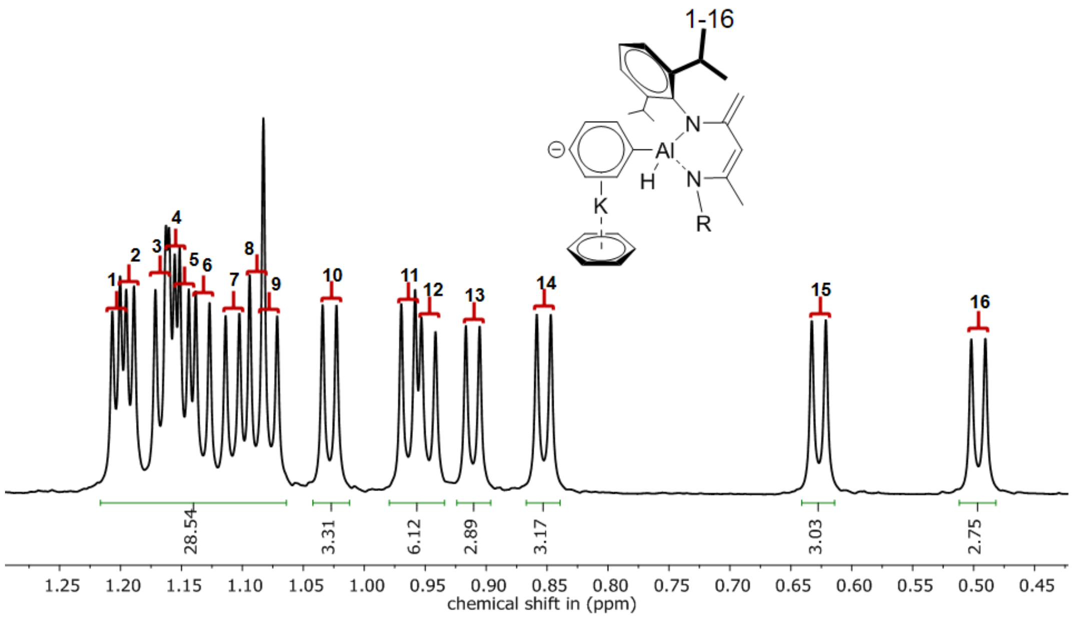

Figure S15: ^{1}H NMR spectrum of **2**, zoomed in on 1.3 to 0.4 ppm. 600 MHz, THF-$d_{8'}$ [25 °C.]

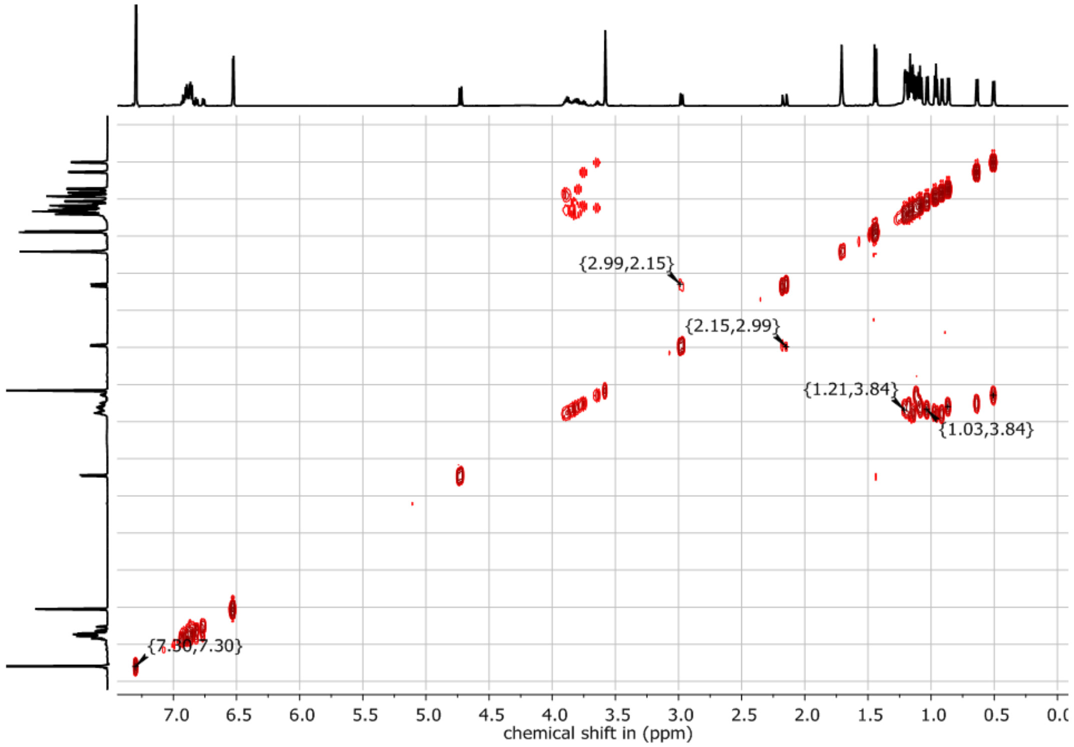

Figure S16: COSY NMR spectrum of **2**. 600 MHz, THF-d_8, 25 °C.

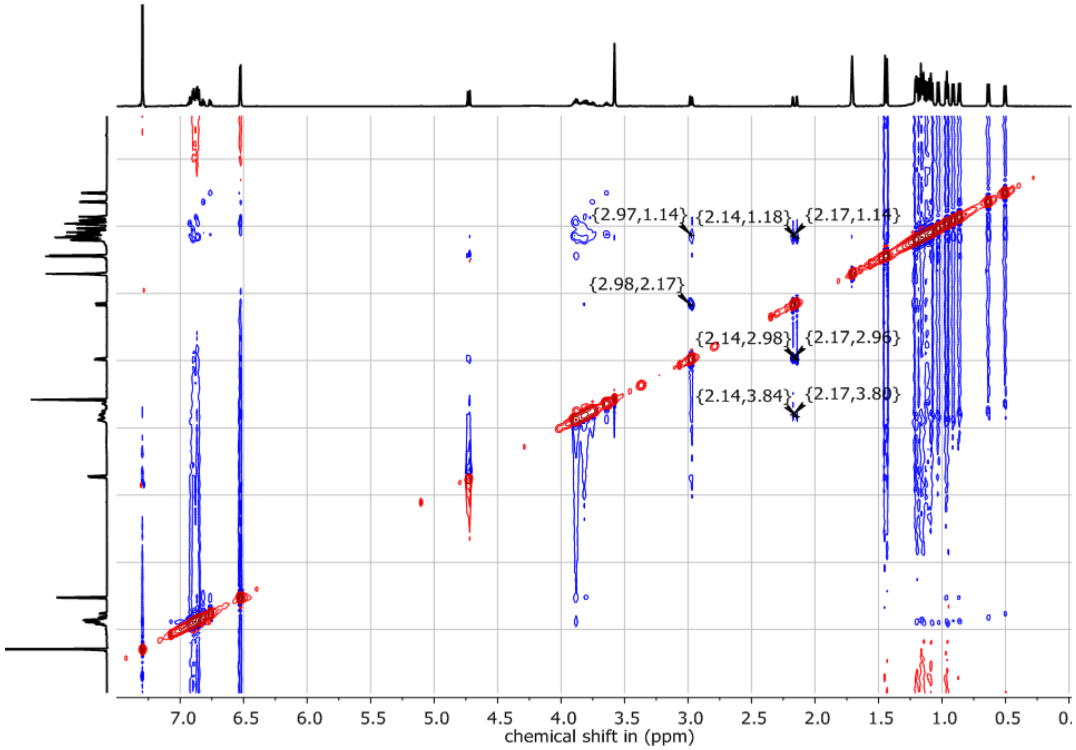

Figure S17: NOESY NMR spectrum on **2**. 600 MHz, THF-d_8, 25 °C.

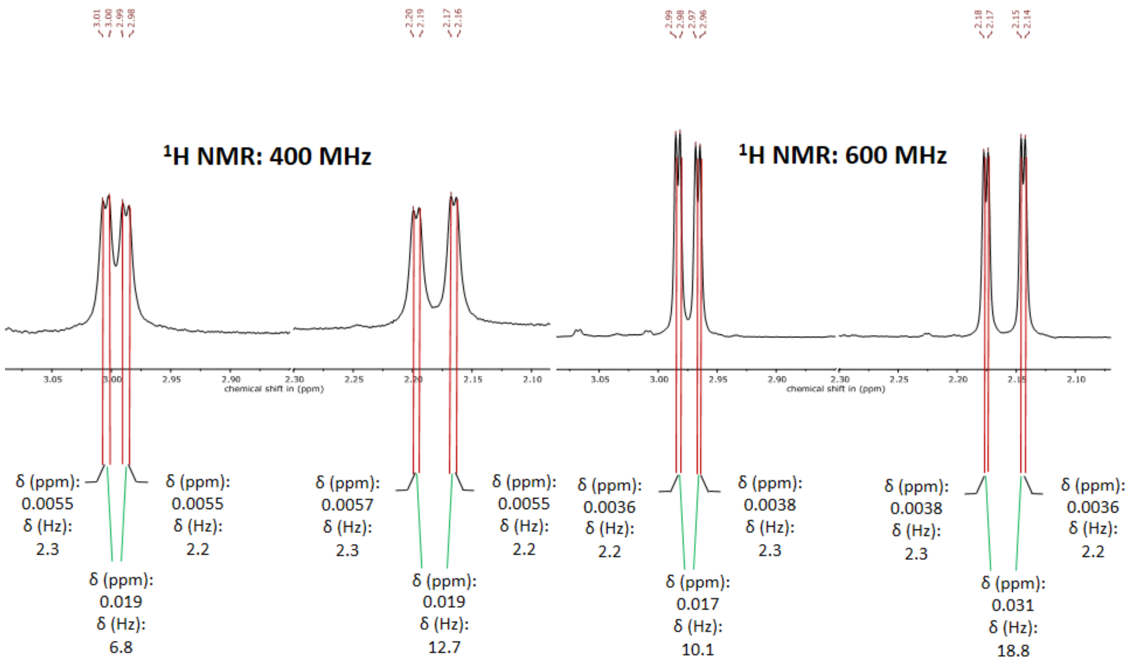

Figure S18: Comparison of ^1H NMR chemical shifts and coupling constants of the C=CH_2 signals of **2** on 400 MHz (left) and 600 MHz (right) NMR machines (THF-d_8, 25 °C).

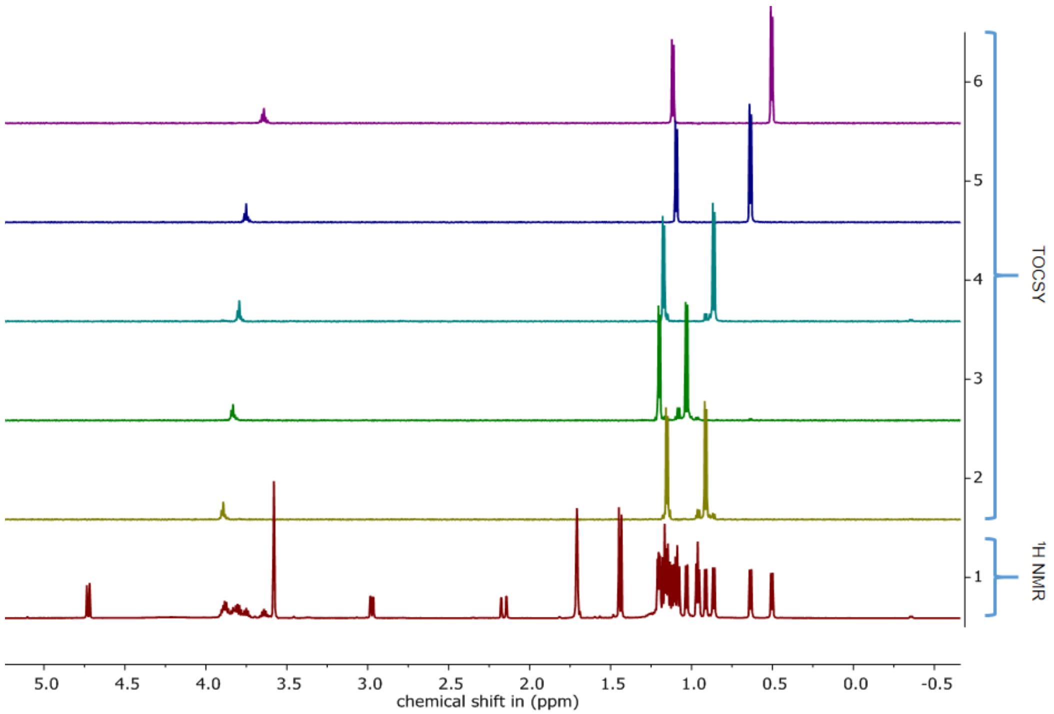

Figure S19: TOCSY NMR experiments. Five out of eight spin systems (iPrCH → iPrCH_3) could be separated.

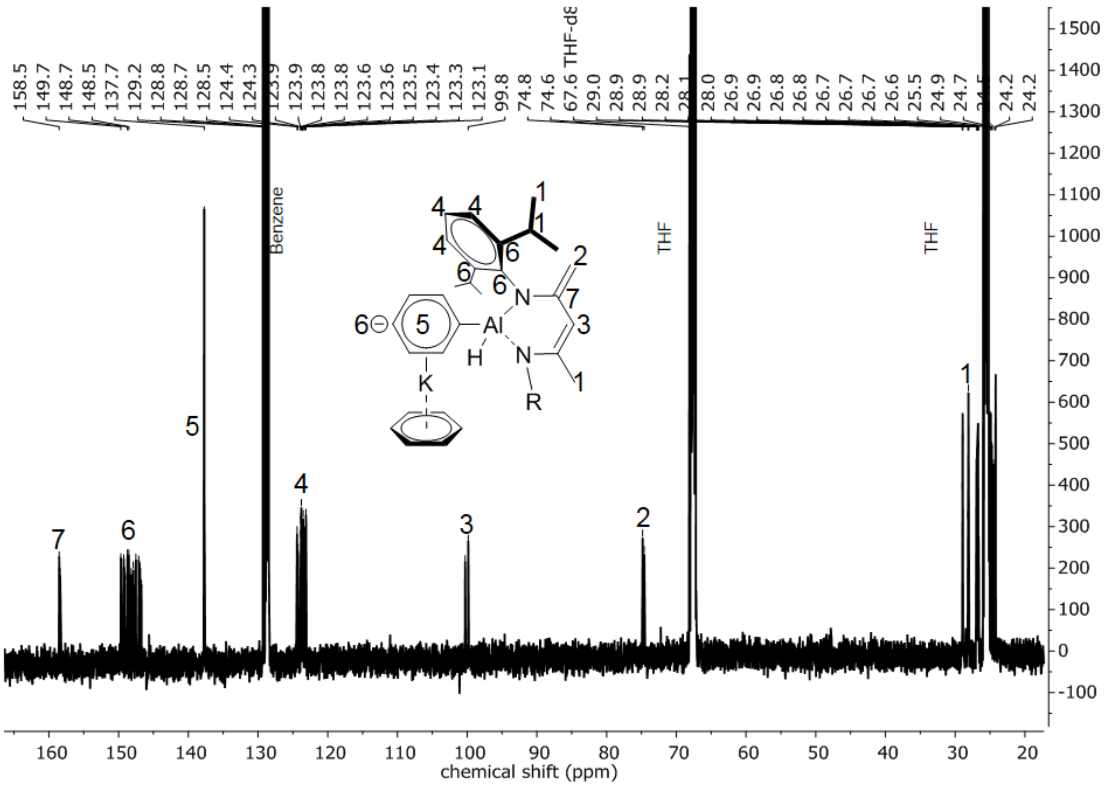

Figure S20: $^{13}C\{^{1}H\}$ NMR spectrum of **2**. 151 MHz, THF-d_8, 25 °C.

2.2. Temperature dependent ^{1}H NMR of complex 2

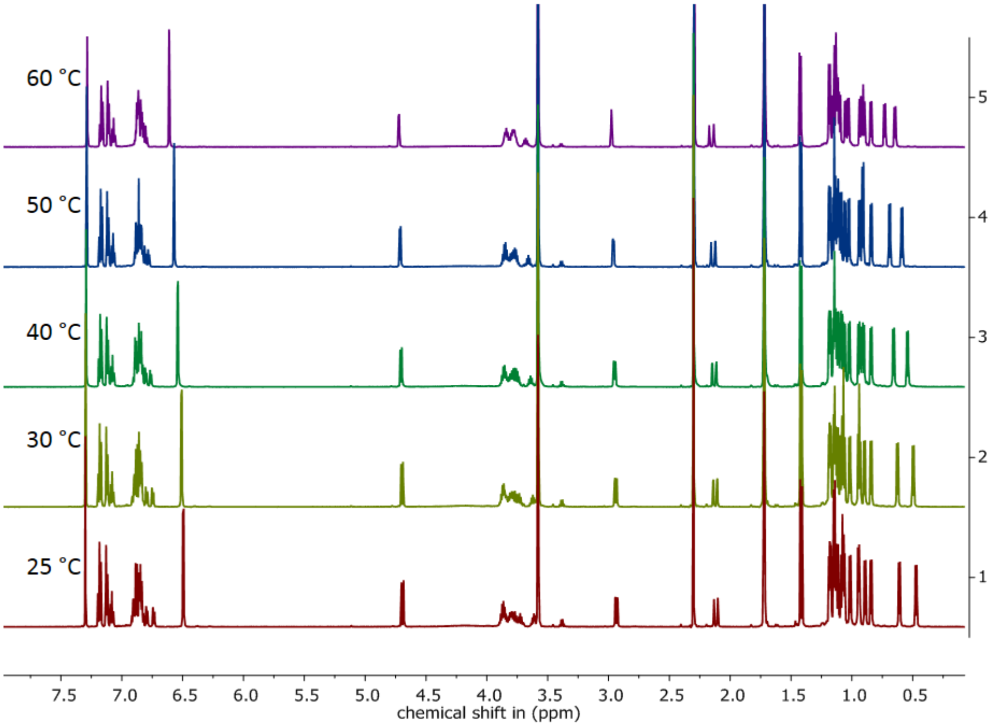

Figure S21: Temperature dependent ^{1}H NMR spectra of **2**. (600 MHz, THF-d_8).

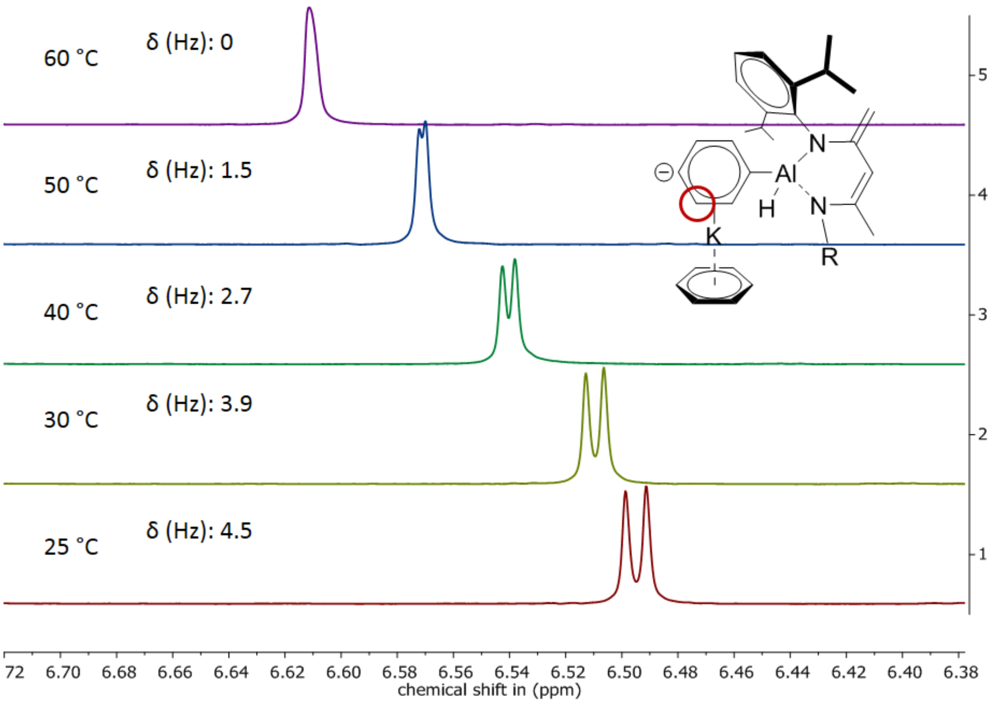

Figure S22: Comparison of temperature dependent ^{1}H NMR measurements of **2** in the range 6.7 – 6.4 ppm. (600 MHz, THF-d_8).

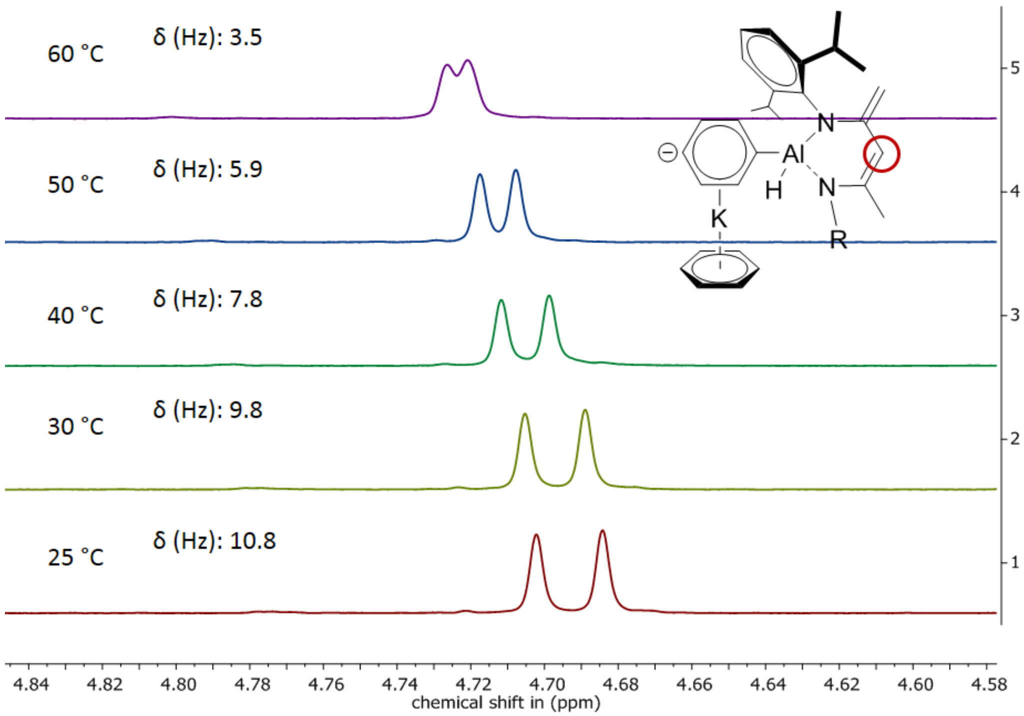

Figure S23: Comparison of temperature dependent ^{1}H NMR measurements of **2** in the range 4.8 – 4.6 ppm. (600 MHz, THF-d_8).

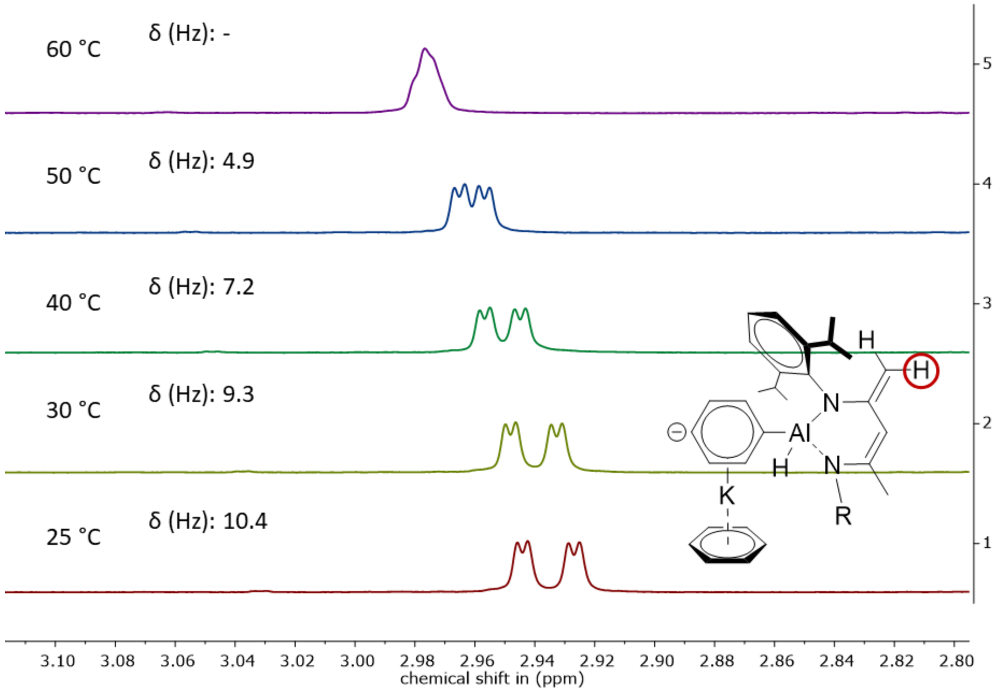

Figure S24: Comparison of temperature dependent ^{1}H NMR measurements of **2** in the range 3.1 – 2.8 ppm. (600 MHz, THF-d_8).

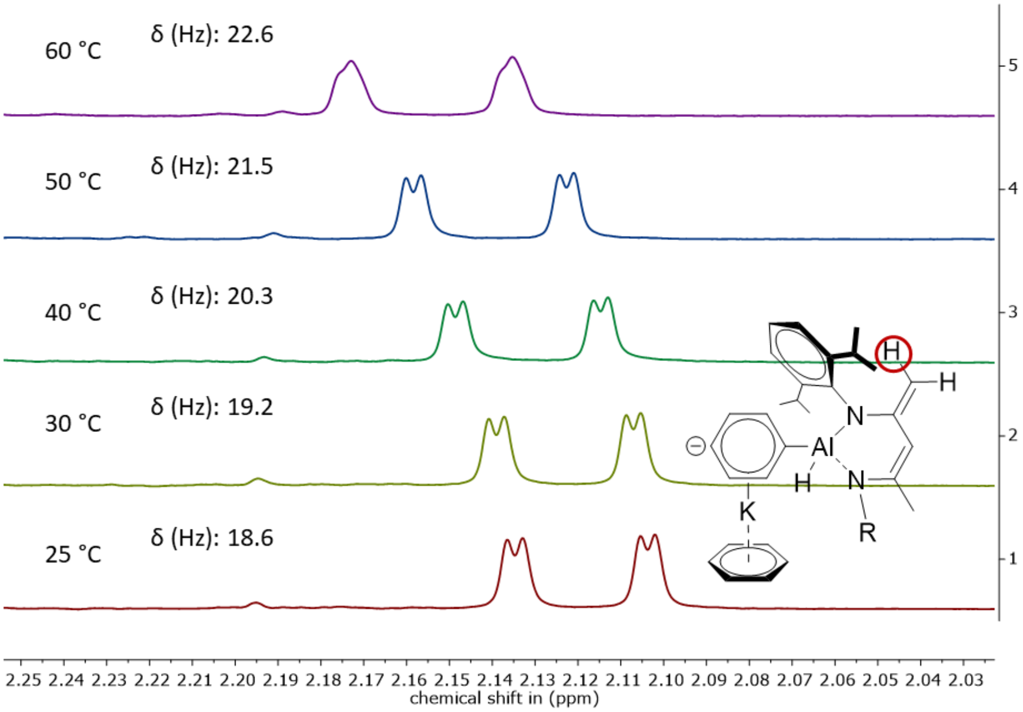

Figure S25: Comparison of temperature dependent ^{1}H NMR measurements of **2** in the range 2.2 – 2.0 ppm. (600 MHz, THF-d_8).

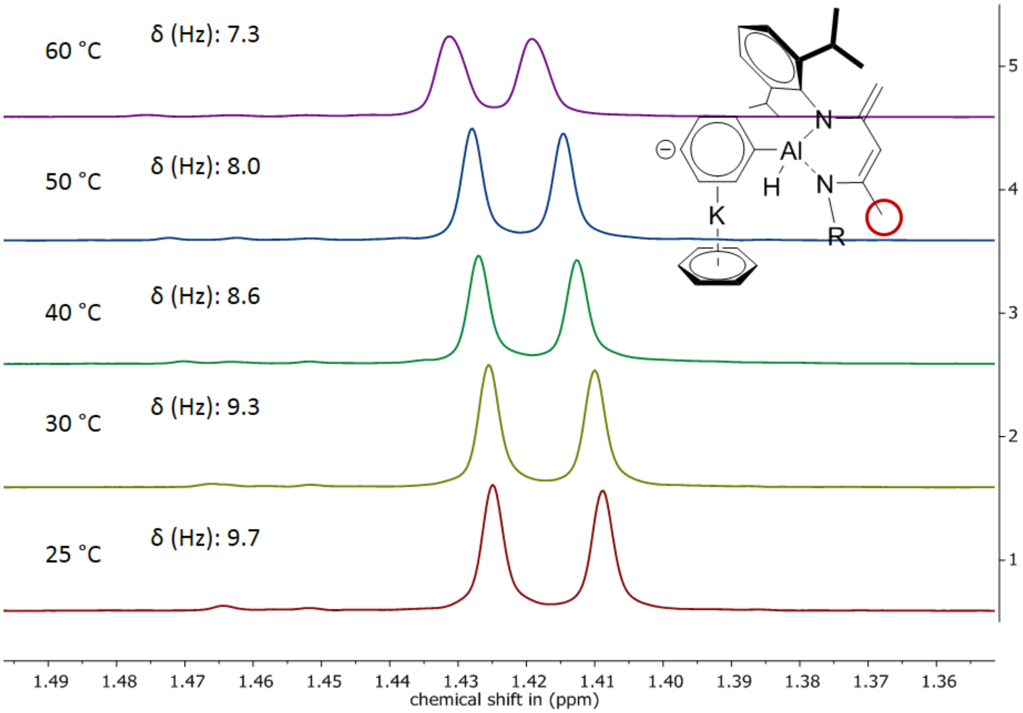

Figure S26: Comparison of temperature dependent ^{1}H NMR measurements of **2** in the range 1.5 – 1.3 ppm. (600 MHz, THF-d_8).

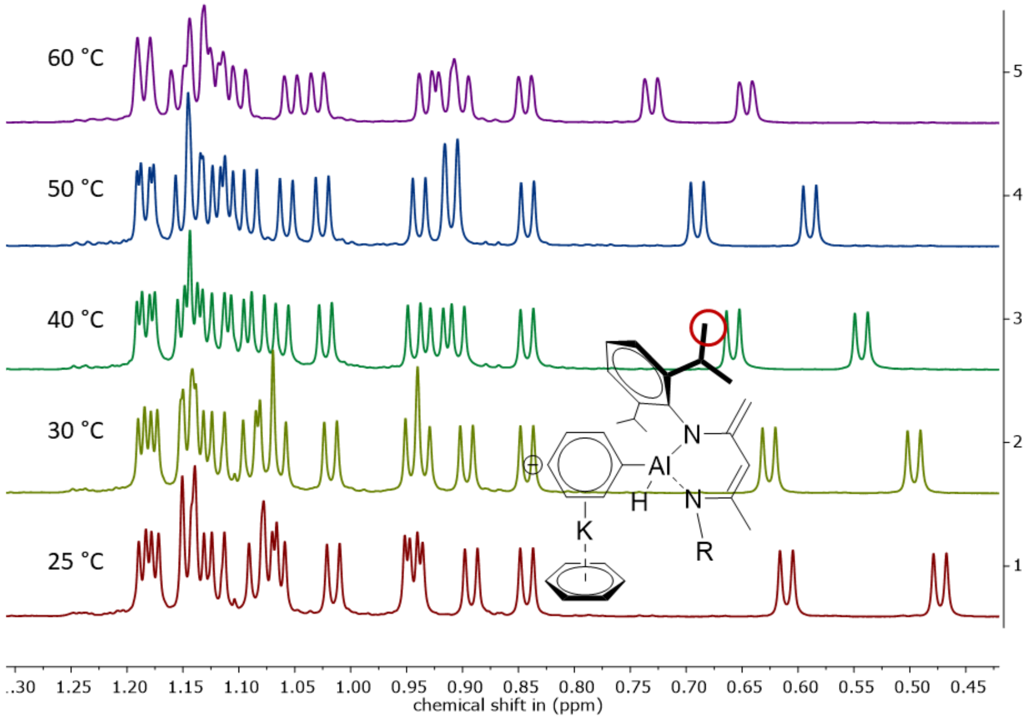

Figure S27: Comparison of temperature dependent ^{1}H NMR measurements of **2** in the range 1.3 – 0.4 ppm. (600 MHz, THF-d_8).

2.3. Diffusion measurements (DOSY)

Diffusion measurements were conducted on a Bruker AVANCE NMR spectrometer operating at 600.13 MHz for proton resonance equipped with a 5 mm PABDO BB/19F-1H/D probe with Z-GRD and actively shielded gradient coil with a maximum gradient strength of 5.3500094 G/mm (at 10 A).

Parameter optimization was carried out empirically employing the pulse programme ledbpgp2s1D using stimulated echo and LED (D21 = 5 ms, longitudinal eddy current delay as a Z-filter) with bipolar gradient pulses (P30) and two spoiling gradients (P19 = 600 µs) leading to values for gradient pulse length (P30 = adjusted [µs], in case of bipolar gradients little DELTA*0.5) and diffusion time (D20 = 60 ms, big DELTA). Delay for gradient recovery was set to 200 µs.

The diffusion experiment was executed with variable gradients from 2% to 98% gradient strength with 32 increment values (difframp calculated with the AU-program DOSY). In this case the pulse program ledbpgp2s was applied for data acquiring of this pseudo-2D Experiment. Data processing was performed with the T1/T2 software package (SimFit) of TopSpin (version 3.2, Bruker Biospin) by fitting area data

(integration of all peaks of interest of the same molecule) of diffusion decays. From these Stejskal-Tanner fitting curves calculated diffusion constants were obtained and assimilated statistically.

Table S1: Results for diffusion measurement of **1** applying ^{1}H DOSY in C_6D_6 and THF-d_8

	**1** in C_6D_6	**1** in THF-d_8
Sum formula	$C_{58}H_{80}N_4Al_2K_2$	$C_{37}H_{56}N_2AlKO_2$
Theoretical molecular weight MW^{Calc} (g/mol)	966 (dimer)	627 (monomer·(thf)$_2$)
Diffusion coefficient D (m/s^2)	$4.509*10^{-10}$	$6.537*10^{-10}$
Determined molecular weight MW^{Exp} (g/mol)	855	635

Comment on the measurements in THF-d_8

TMS was added as internal reference. The MW estimation was carried out following the method described in ref. S5. An own calibration curve (DMW) confirm the results obtained with Stalke´s method.

Some signals for complex **1** in THF-d_8 are affected strongly by signals of minor amounts of a decomposition product of very high MW (> 25.000) which leads to a considerable corruption of the diffusion coefficients. Apart from that, the obtained DOSY data seem reasonable as they suggest the presence of a monomeric aluminum complex with two THF ligands. The quality of the measurement is displayed and underlined by a reasonable molecular weight estimation of the THF molecule from its diffusion coefficient which also indicates a weakly bound THF ligand. Nevertheless, as the measurement was carried out in THF solution, associated THF molecules have always to be considered in the calculated molecular weights of complexes.

Table S2: Results for diffusion measurement of the decomposition product of **1** with THF-d_8 applying ^{1}H DOSY THF-d_8

	Decomposition product of **1** in THF-d_8
Sum formula	-
Theoretical molecular weight MW^{Calc} (g/mol)	-
Diffusion coefficient D (m/s^2)	$7.373*10^{-11}$
Determined molecular weight MW^{Exp} (g/mol)	26923

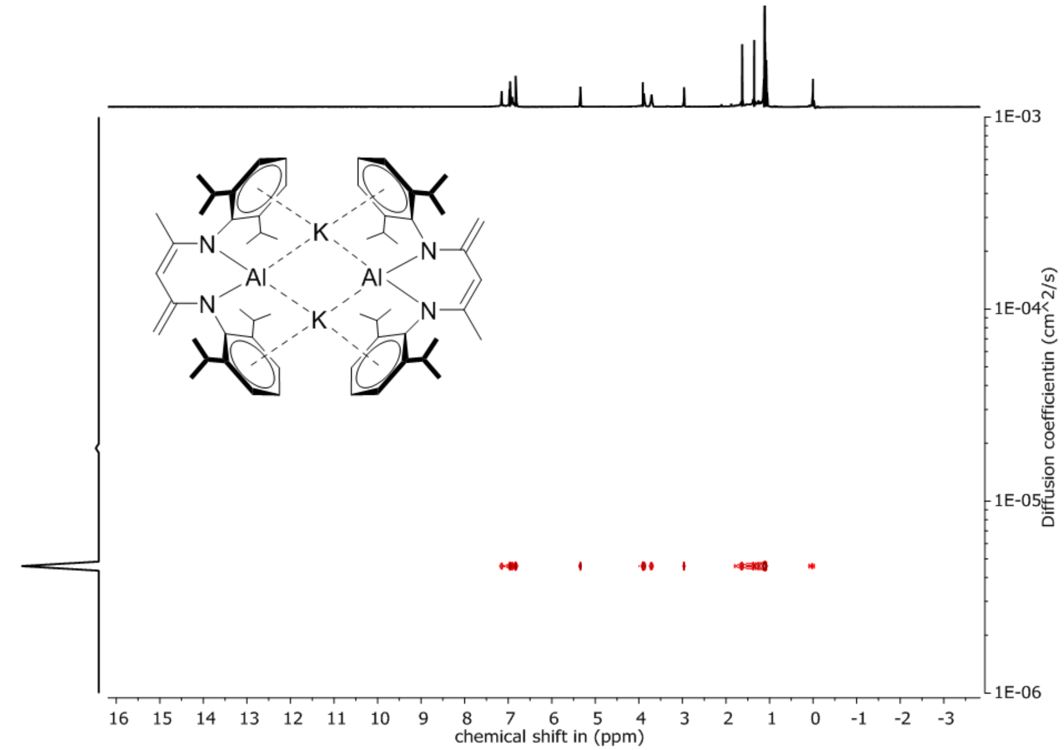

Figure S28: ^{1}H DOSY NMR spectrum of **1** in C_6D_6.

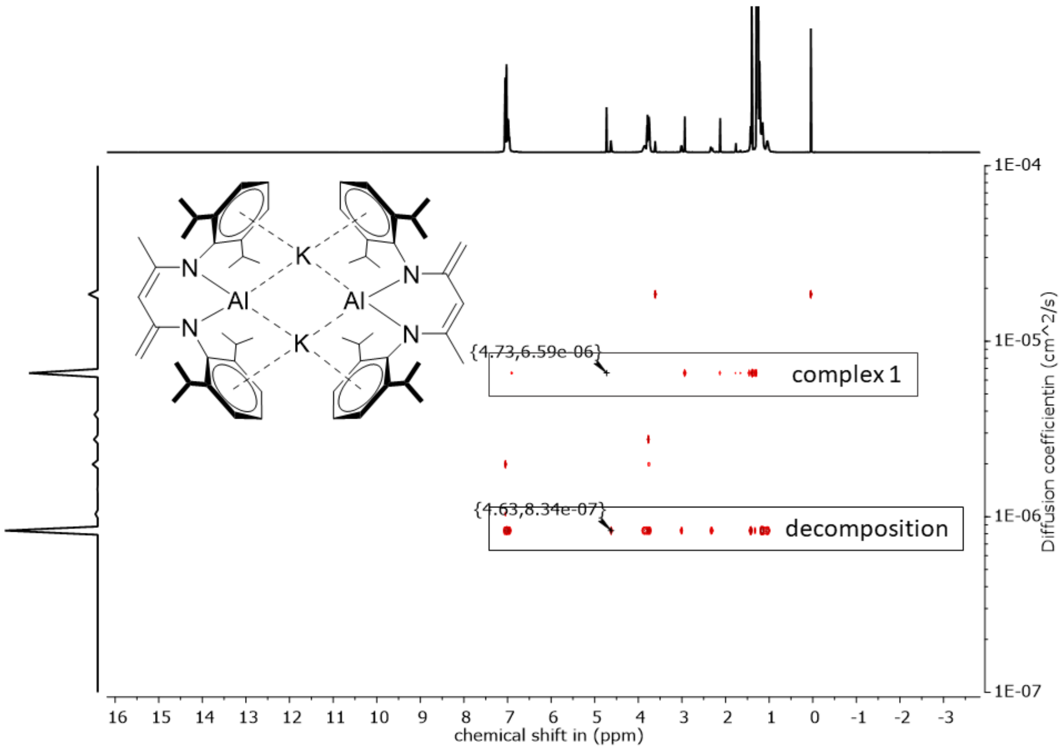

Figure S29: ^{1}H DOSY NMR spectrum of **1** in THF-d_8.

3. Computational Details

3.1 General methods

All calculations were carried out using Gaussian 16A.(S6) All methods were used as implemented. All structures were fully optimized on a ωB97XD/6-31+G** level of theory.(S7-9) All structures were characterized as true minima (Nimag=0) or transition states (Nimag=1) by frequency calculations on the same level of theory. Energies were determined at a ωB97XD/6-311+G** level of theory. Solvation effects were approximated using a PCM field of benzene.(S10) Charges and bond orders were calculated via NBO Analyses.(S11) All structures were evaluated using Molecule 2.3.(S12) Topological analyses were carried out with AIMAll17 using the wave function of the optimization.(S13-14) For comparison with earlier reported values, the HOMO-LUMO's have been calculated at the B3LYP/6-31+G* level of theory.

3.2 Summary of results

Comparison of calculated structure of 1 and its crystal structure

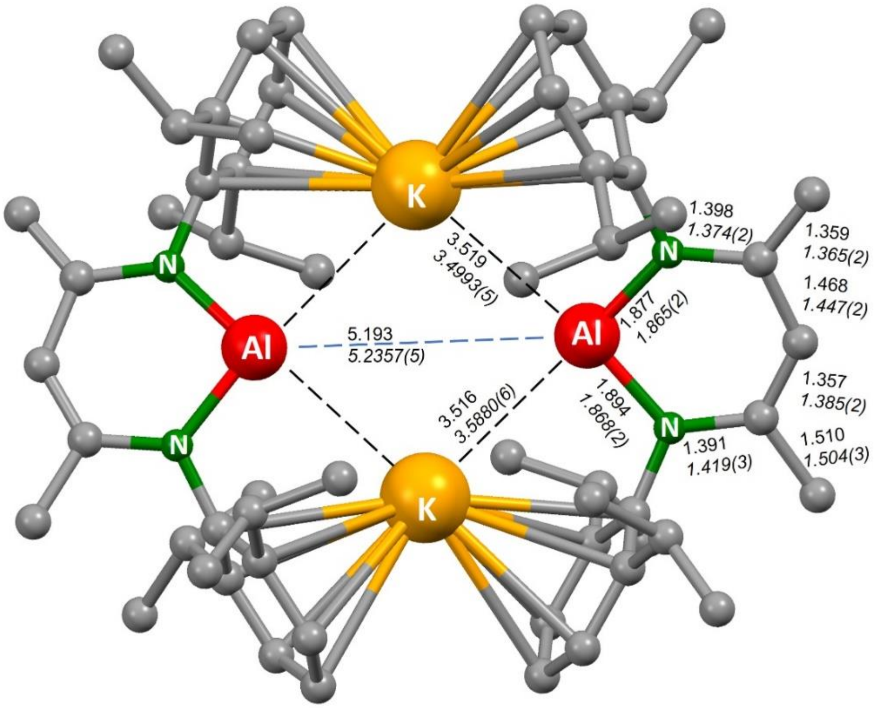

Figure S30: Comparison of calculated structure and crystal structure (in italic) of **1**.

HOMO and LUMO molecular orbitals and energies for 1

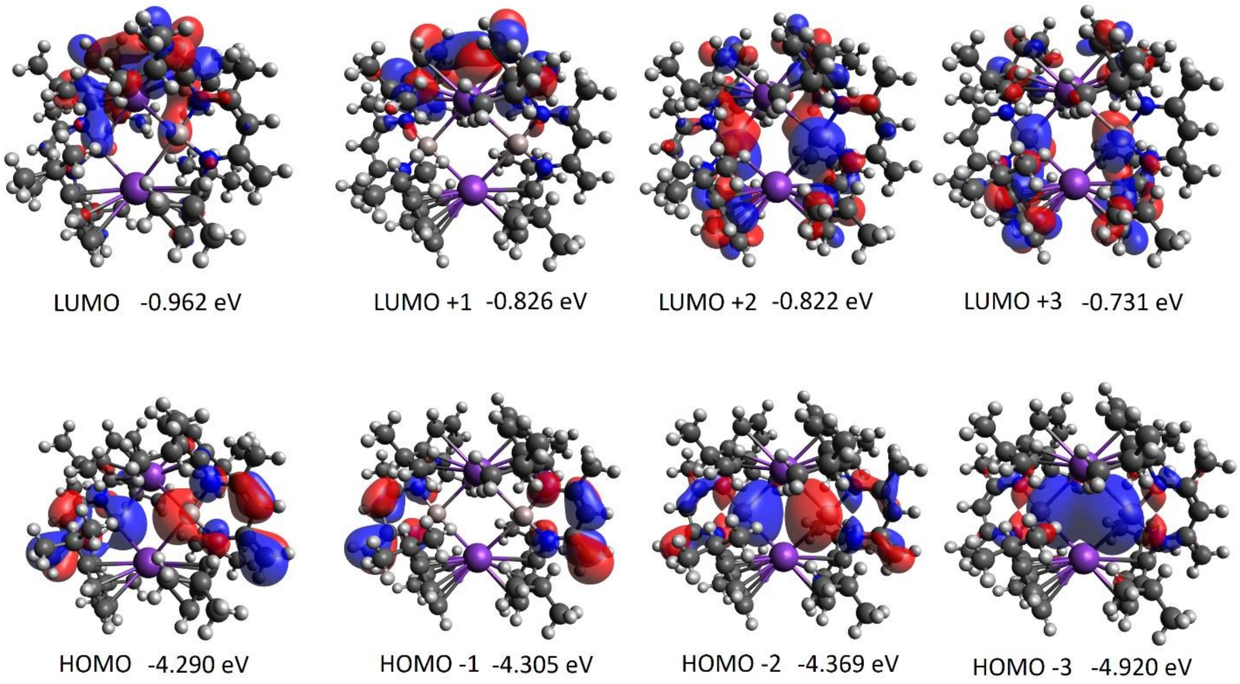

Figure S31: HOMO/LUMO energies and representations for complex **1** calculated at the B3LYP/6-31+G* level.

Formation of 1 by deprotonation of (^{DIPP}BDI)Al with $KN(SiMe_3)_2$

Table S3. Relative energies for deprotonation of (^{DIPP}BDI)Al by $KN(SiMe_3)_2$ and dimerization to **1**.

	ΔE	ΔH	ΔG
Complex [(^{DIPP}BDI)Al] (**A**)	0.0	0.0	0.0
Al(I)+$KN(SiMe_3)_2$-Adduct (**B**)	-27.3	-22.7	-1.3
TS* (**C**)	1.8	1.4	19.9
[(^{Ph}BDI)Al^-K^+] (**D**)	-5.5	-4.9	-2.5
[(^{Ph}BDI)Al^-K^+]$_2$ (**E**)	-42.3	-40.1	-17.6

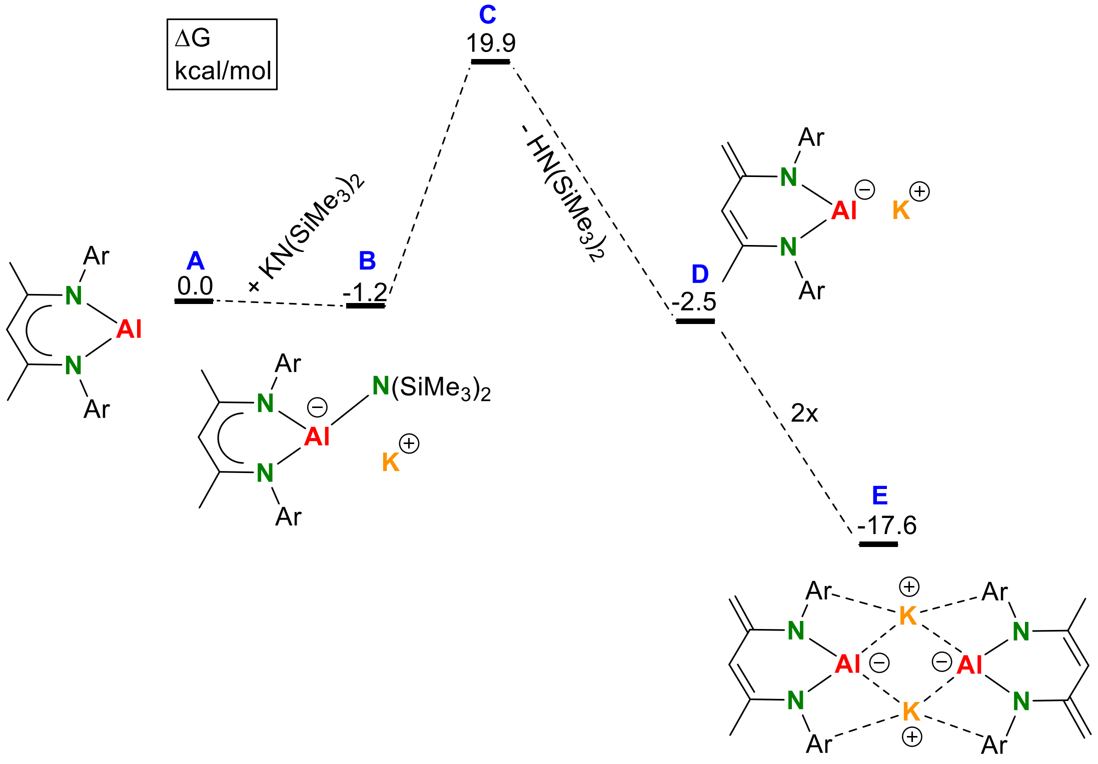

Figure S32. Energy profile for deprotonation of (^{DIPP}BDI)Al by $KN(SiMe_3)_2$ and dimerization to **1**.

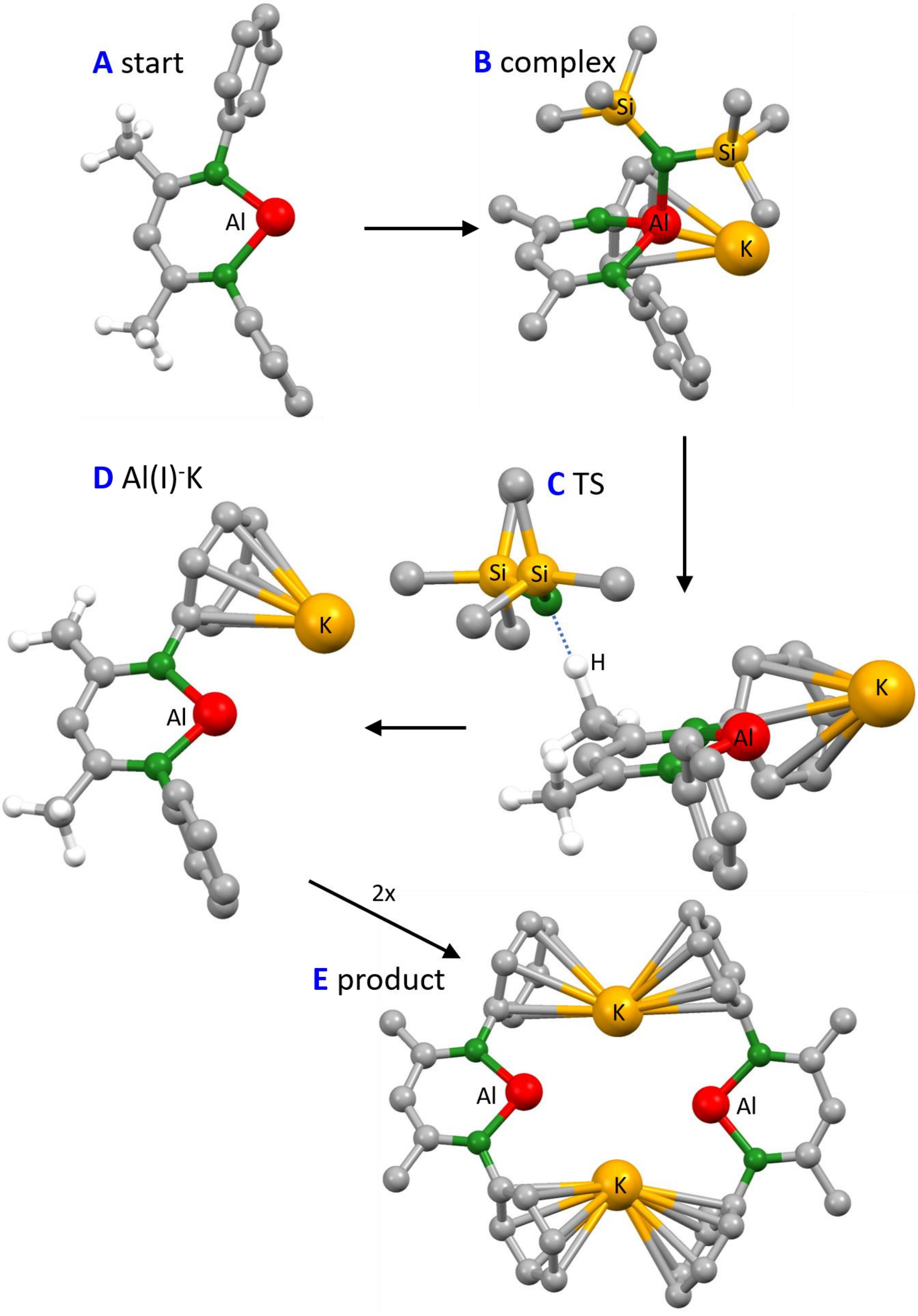

Figure S33. Optimized complexes on the pathway for deprotonation of (^{DIPP}BDI)Al by $KN(SiMe_3)_2$ and dimerization to **1**, *i*Pr-groups omitted for clarity.

Benzene C-H activation by neutral (^{DIPP}BDI)Al

Table S4. Relative energies for C-H activation of benzene by the neutral (^{DIPP}BDI)Al.

	ΔE	ΔH	ΔG
Complex (^{DIPP}BDI)Al (**A**)	0.0	0.0	0.0
Benzene adduct (**B**)	-6.5	-5.6	7.6
TS1* (**C**)	38.8	36.0	51.0
[(^{DIPP}BDI)Al(H)Ph] (**D**)	-40.4	-40.6	-25.0

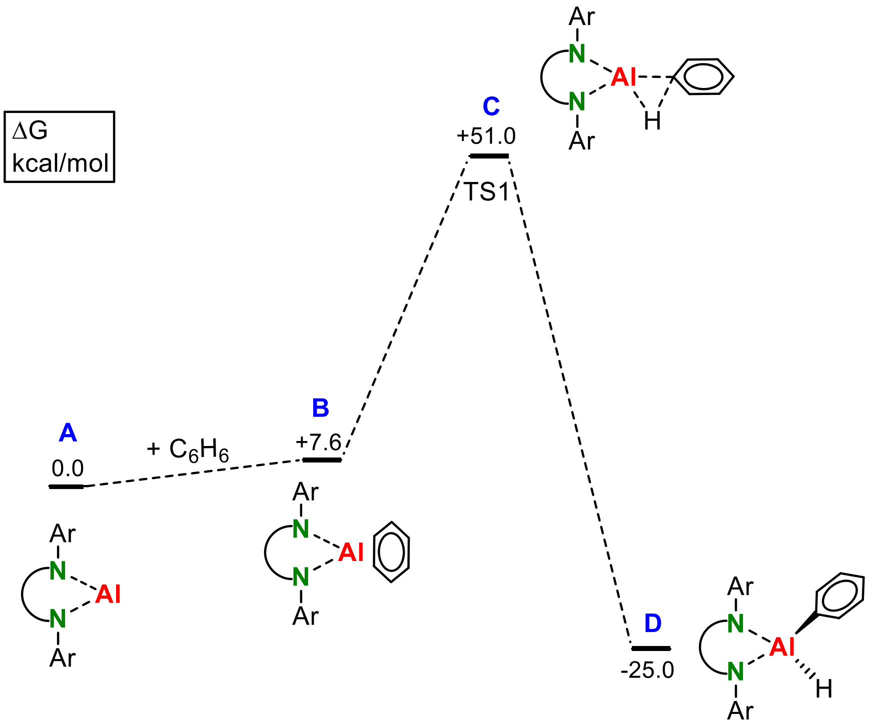

Figure S34. Energy profile for C-H activation of benzene by the neutral (^{DIPP}BDI)Al.

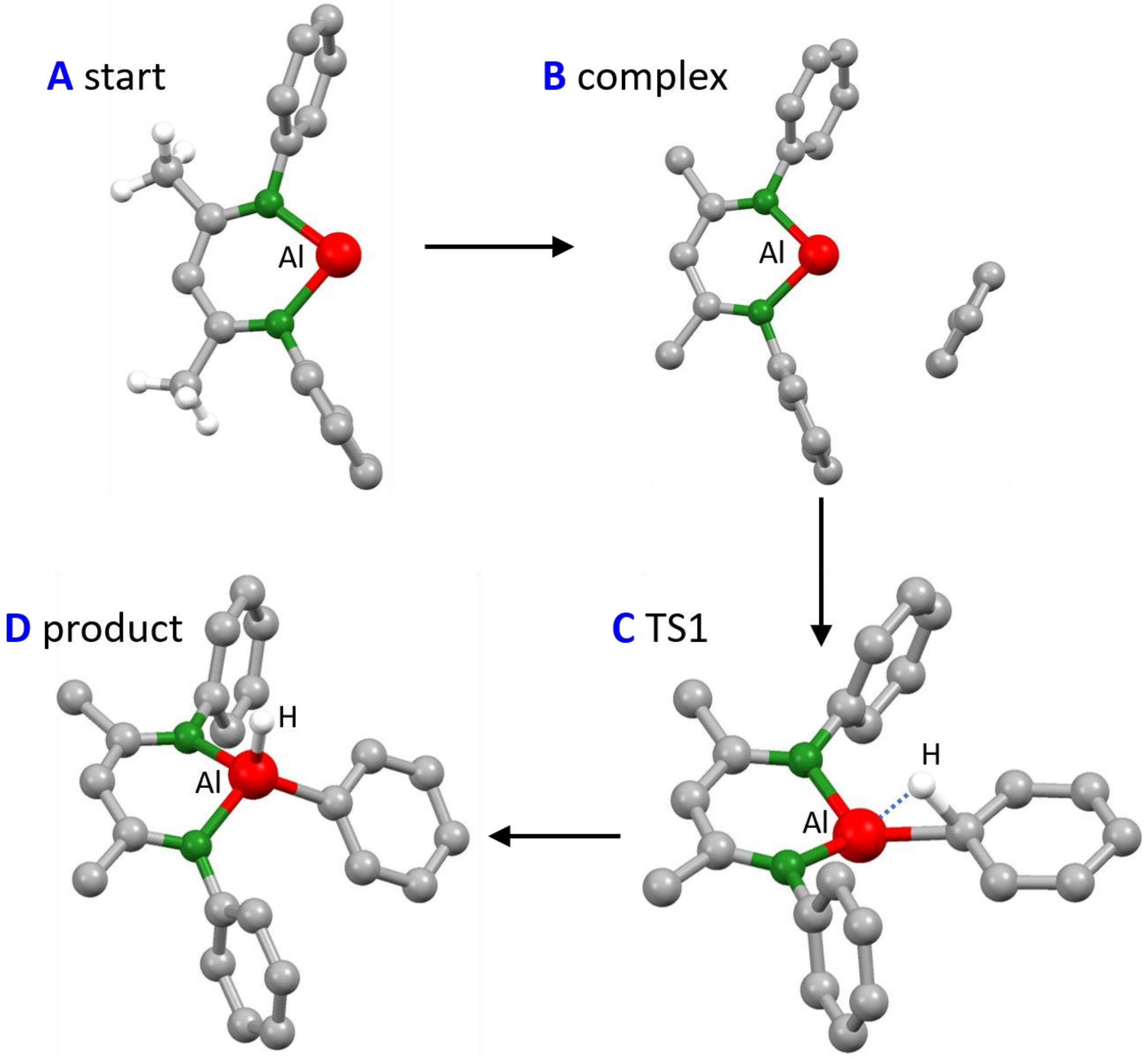

Figure S35. Optimized complexes on the pathway for C-H activation of benzene by the neutral (^{DIPP}BDI)Al, *i*Pr-groups omitted for clarity.

Benzene C-H activation by anionic (^{DIPP}BDI-H)Al$^{–}$

^{DIPP}BDI-H = deprotonated ^{DIPP}BDI anion

Table S5. Relative energies for C-H activation of benzene by the anionic (^{DIPP}BDI-H)Al^{-}.

	ΔE	ΔH	ΔG
Complex [(^{DIPP}BDI-H)Al(I)]$^{-}$ (**A**)	0.0	0.0	0.0
Benzene adduct (**B**)	-7.3	-6.6	4.1
TS1* (**C**)	21.1	18.2	31.6
[(^{DIPP}BDI-H)Al(H)Ph]$^{-}$ (**D**)	-41.7	-42.7	-27.8

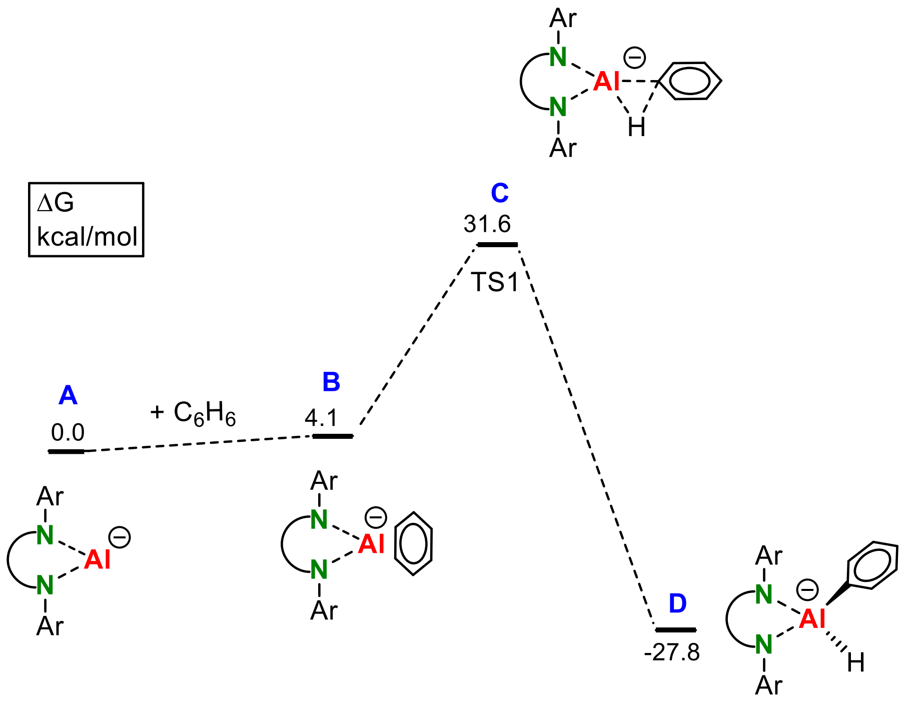

Figure S36. Energy profile for C-H activation of benzene by the anionic (^{DIPP}BDI-H)Al^{-}.

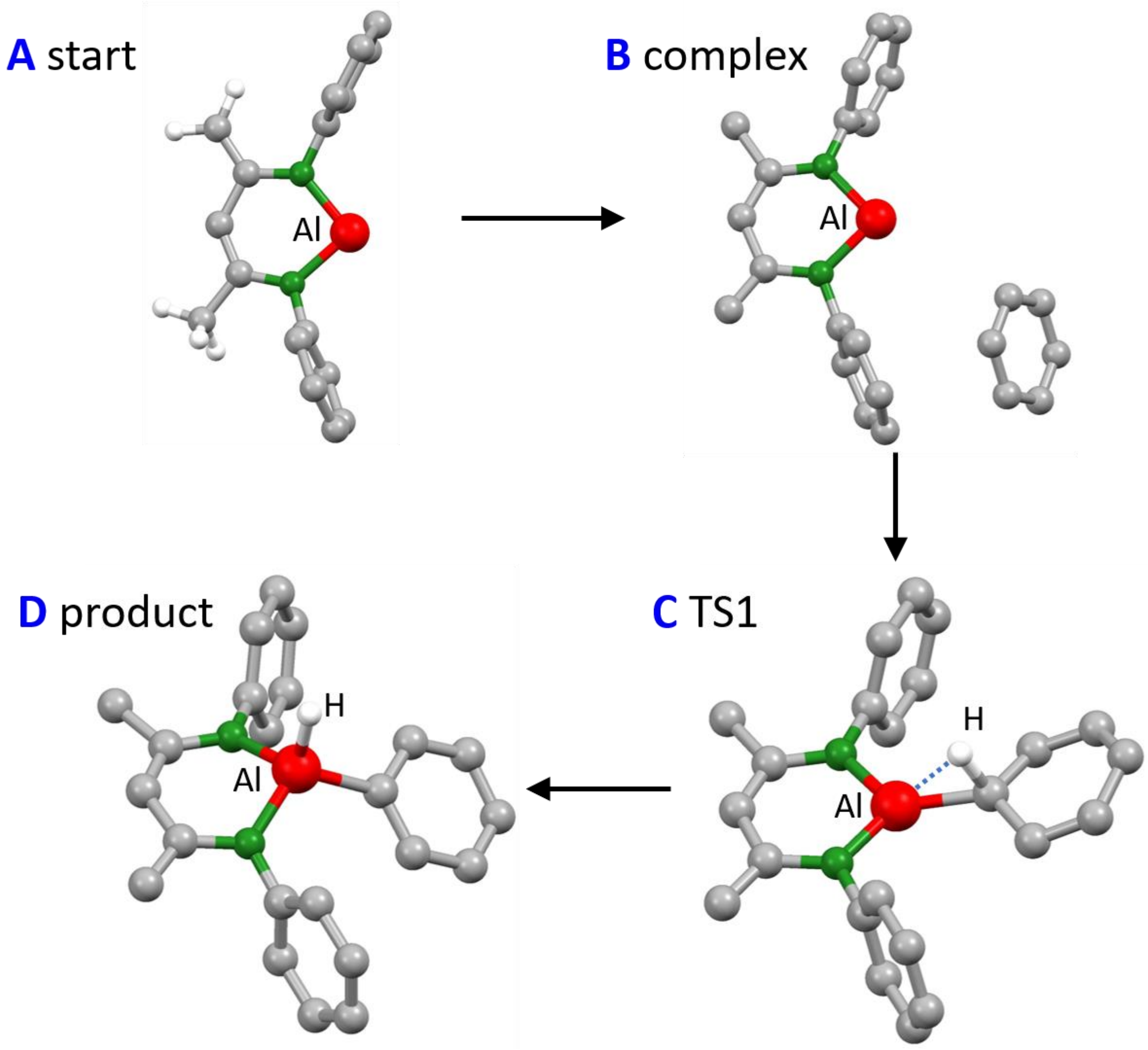

Figure S37. Optimized complexes on the pathway for C-H activation of benzene by anionic (^{DIPP}BDI-H)Al^{-}, *i*Pr-groups omitted for clarity.

Benzene C-H activation by monomeric (^{DIPP}BDI-H)Al^{-}K^{+}

Table S6. Relative energies for C-H activation of benzene by monomeric (^{DIPP}BDI-H)Al^{-}K^{+}.

	ΔE	ΔH	ΔG
Complex [(^{DIPP}BDI-H)Al^{-}K^{+}] (**A**)	0.0	0.0	0.0
Benzene adduct (**B**)	-9.7	-9.1	2.6
Meisenheimer Intermediate (**C**)	8.0	7.8	22.8
TS1* (**D**)	19.1	16.1	30.3
[(^{DIPP}BDI-H)Al(H)Ph^{-}K^{+}] (**E**)	-38.7	-39.8	-24.0

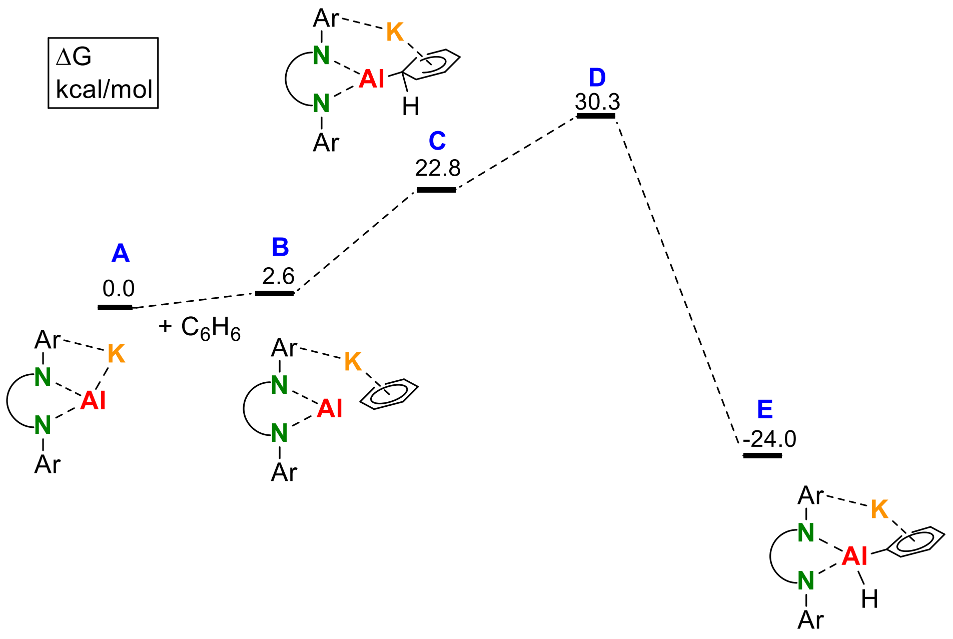

Figure S38. Energy profile for C-H activation of benzene by the monomeric (^{DIPP}BDI-H)Al^{-}K^{+}.

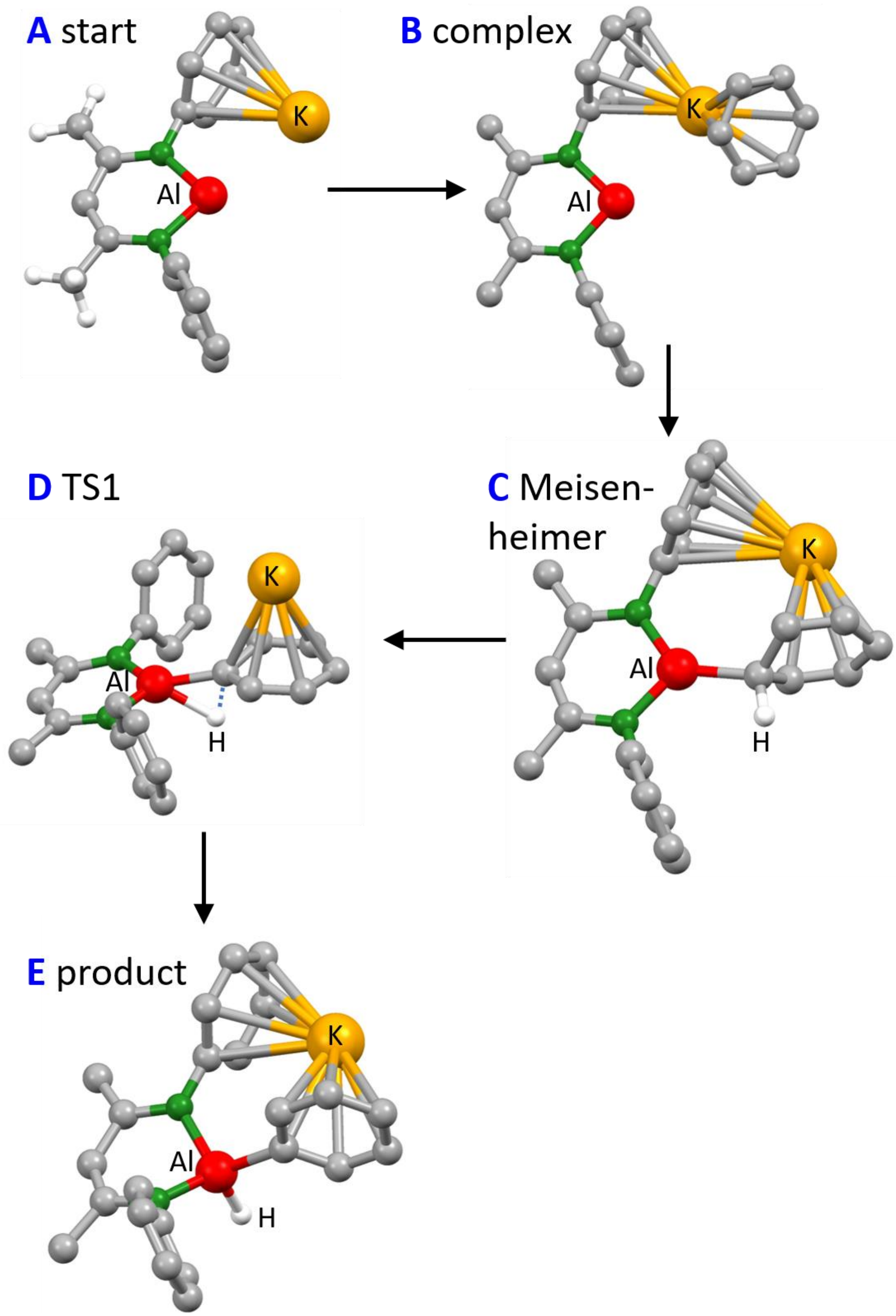

Figure S39. Optimized complexes on the pathway for C-H activation of benzene by the monomeric (^{DIPP}BDI-H)$Al^{-}K^{+}$, *i*Pr-groups omitted for clarity.

C-H activation of Ph-AlH_3^- by anionic (^{DIPP}BDI-H)Al^-

Table S7. Relative energies for second C-H activation of aluminated-benzene by anionic (^{DIPP}BDI-H)Al^-.

	ΔE	ΔH	ΔG
Complex [(^{DIPP}BDI-H)Al^-] (**A**)	0.0	0.0	0.0
Benzene adduct (**B**)	26.6	26.5	43.4
TS1* (**C**)	53.4	51.4	67.1
[(^{DIPP}BDI-H)Al(H)PhAlH_3]$^{2-}$ (**D**)	-23.2	-24.5	-7.8

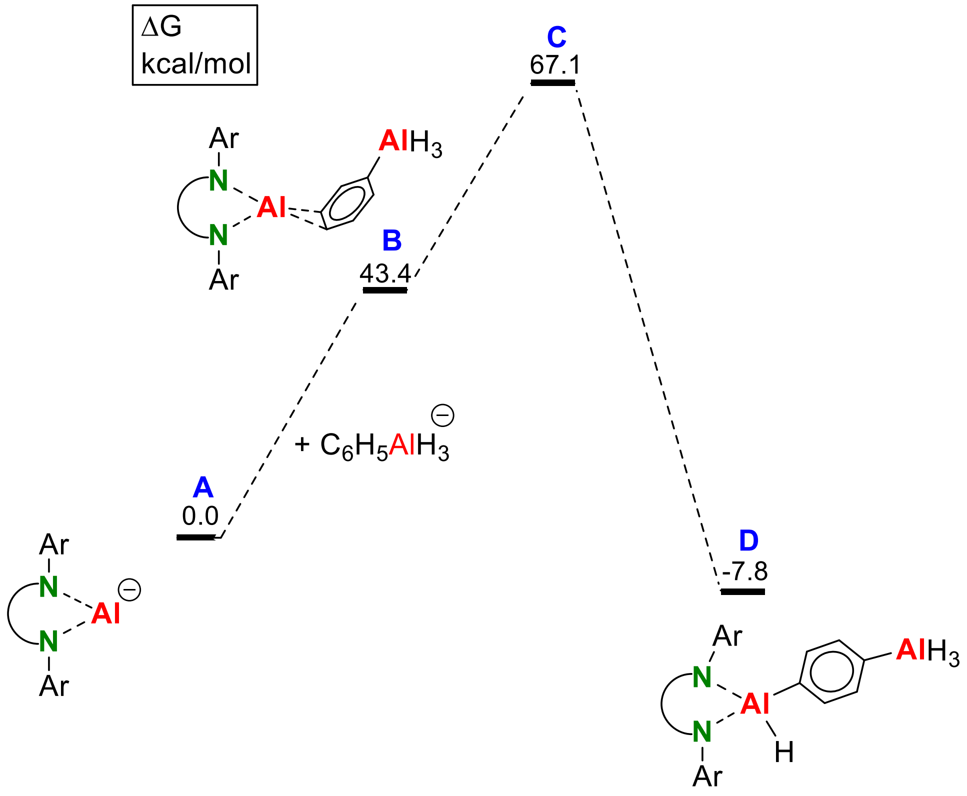

Figure S40. Energy profile for second C-H activation of aluminated-benzene by anionic (^{DIPP}BDI-H)Al^-.

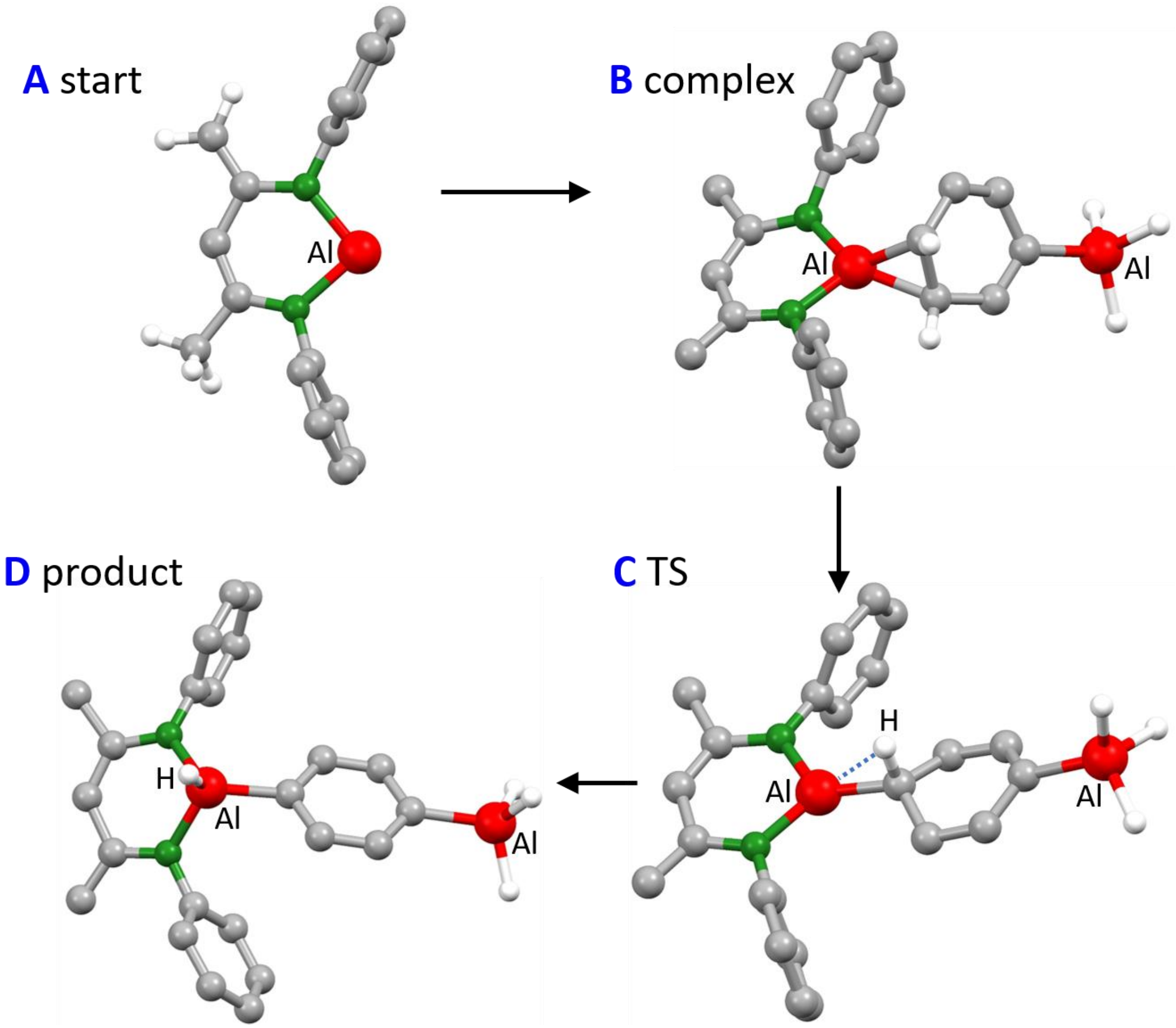

Figure S41. Optimized complexes on the pathway for second C-H activation of aluminated-benzene by anionic (^{DIPP}BDI-H)Al^{-}, *i*Pr-groups omitted for clarity.

C-H activation of Ph-AlH_3^- by monomeric (^{DIPP}BDI-H)Al^-K^+

Table S8. Relative energies for second C-H activation of aluminated-benzene by the monomeric (^{DIPP}BDI)Al^-K^+.

	ΔE	ΔH	ΔG
Complex [(^{DIPP}BDI-H)Al^-K^+] (**A**)	0.0	0.0	0.0
Benzene adduct (**B**)	-0.9	-0.1	18.5
TS1* (**C**)	27.4	24.5	41.9
[(^{DIPP}BDI-H)Al(H)Ph$AlH_3^-K^+$] (**D**)	-49.1	-49.7	-31.6

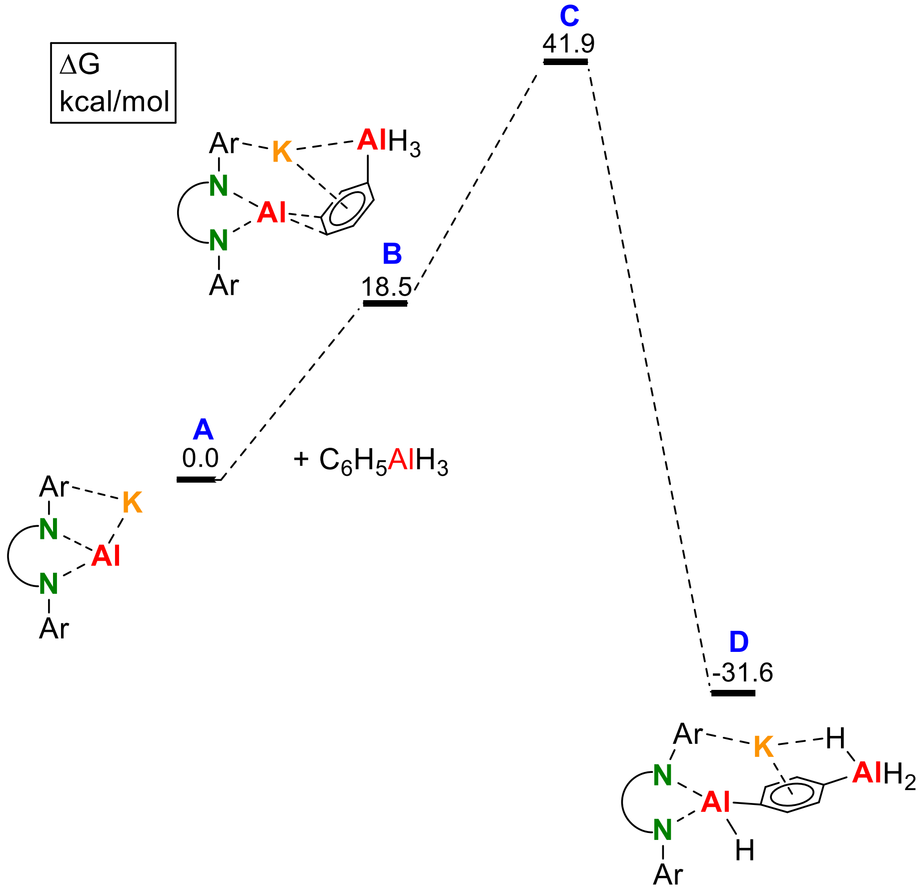

Figure S42. Energy profile for second C-H activation of aluminated-benzene by the monomeric (^{DIPP}BDI-H)Al^-K^+.

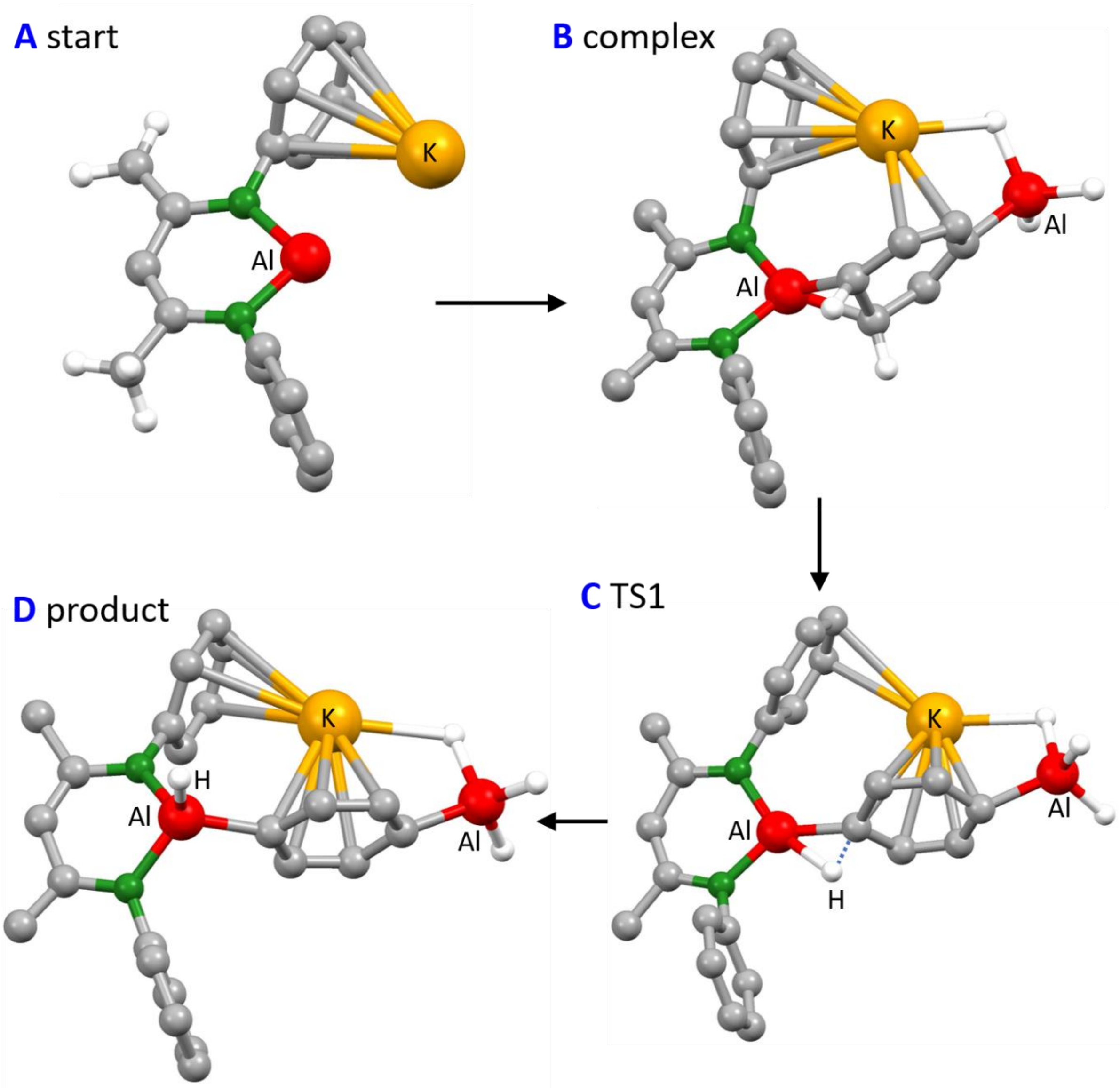

Figure S43. Optimized complexes on the pathway for second C-H activation of aluminated-benzene by the monomeric (^{DIPP}BDI-H)$Al^{-}K^{+}$, *i*Pr-groups omitted for clarity.

Double C-H activation of benzene by dimeric $[(^{Ph}BDI\text{-}H)Al^{-}K^{+}]_2$

Table S9. Relative energies for double C-H activation of benzene by the dimer $[(^{Ph}BDI\text{-}H)Al^{-}K^{+}]_2$.

	ΔE	ΔH	ΔG
Complex $[(^{Ph}BDI\text{-}H)Al^{-}K^{+}]_2$ (**A**)	0.0	0.0	0.0
Benzene adduct (**B**)	-16.1	-14.7	0.5
TS1* (**C**)	17.4	14.8	32.3
$[(^{Ph}BDI\text{-}H)Al(H)Ph][(^{Ph}BDI\text{-}H)Al^{-}K^{+}]$ (**D**)	-41.4	-41.1	-25.9
TS2* (**E**)	-6.5	-8.7	8.4
$[(^{Ph}BDI\text{-}H)Al(H)C_6H_4(H)Al(^{DIPP}BDI\text{-}H)K_2]$ (**F**)	-71.5	-75.0	-58.5

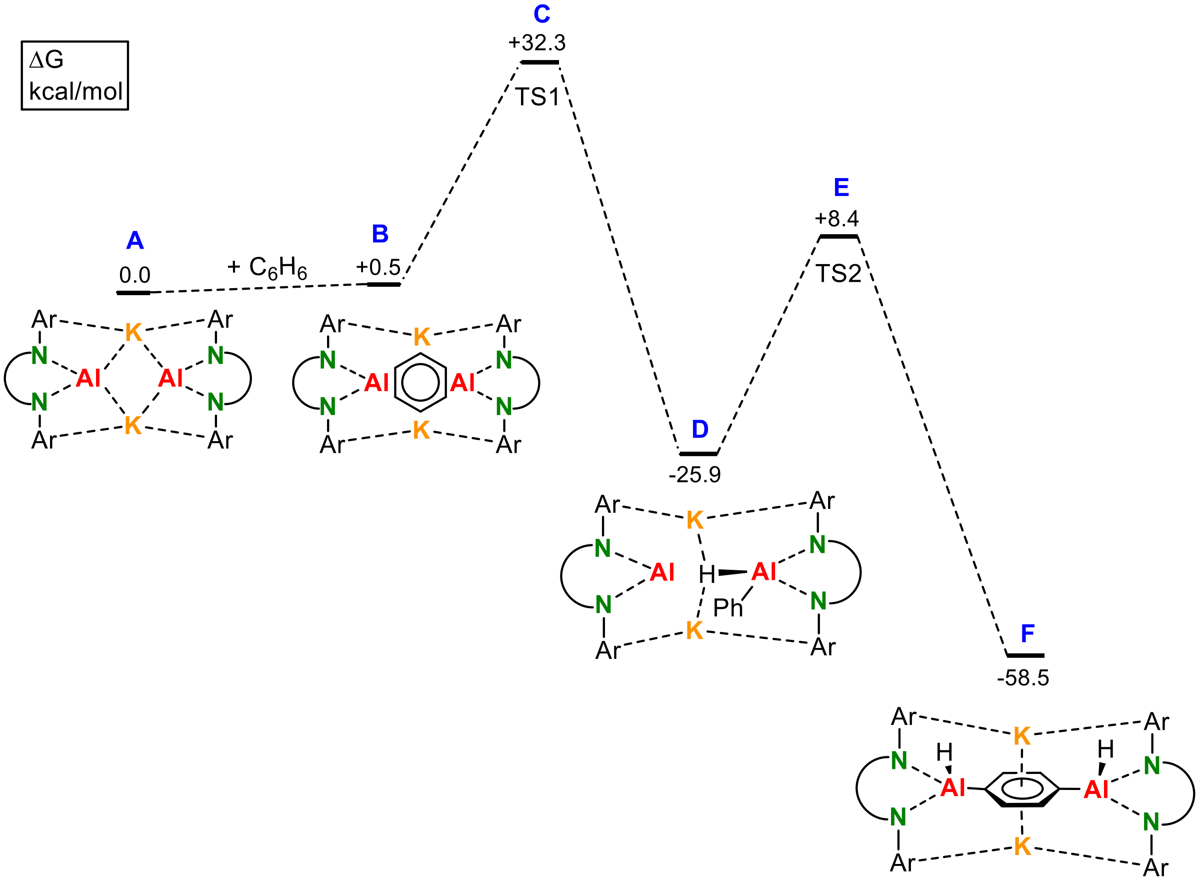

Figure S44. Energy profile for double C-H activation of benzene by the dimer $[(^{Ph}BDI\text{-}H)Al^{-}K^{+}]_2$.

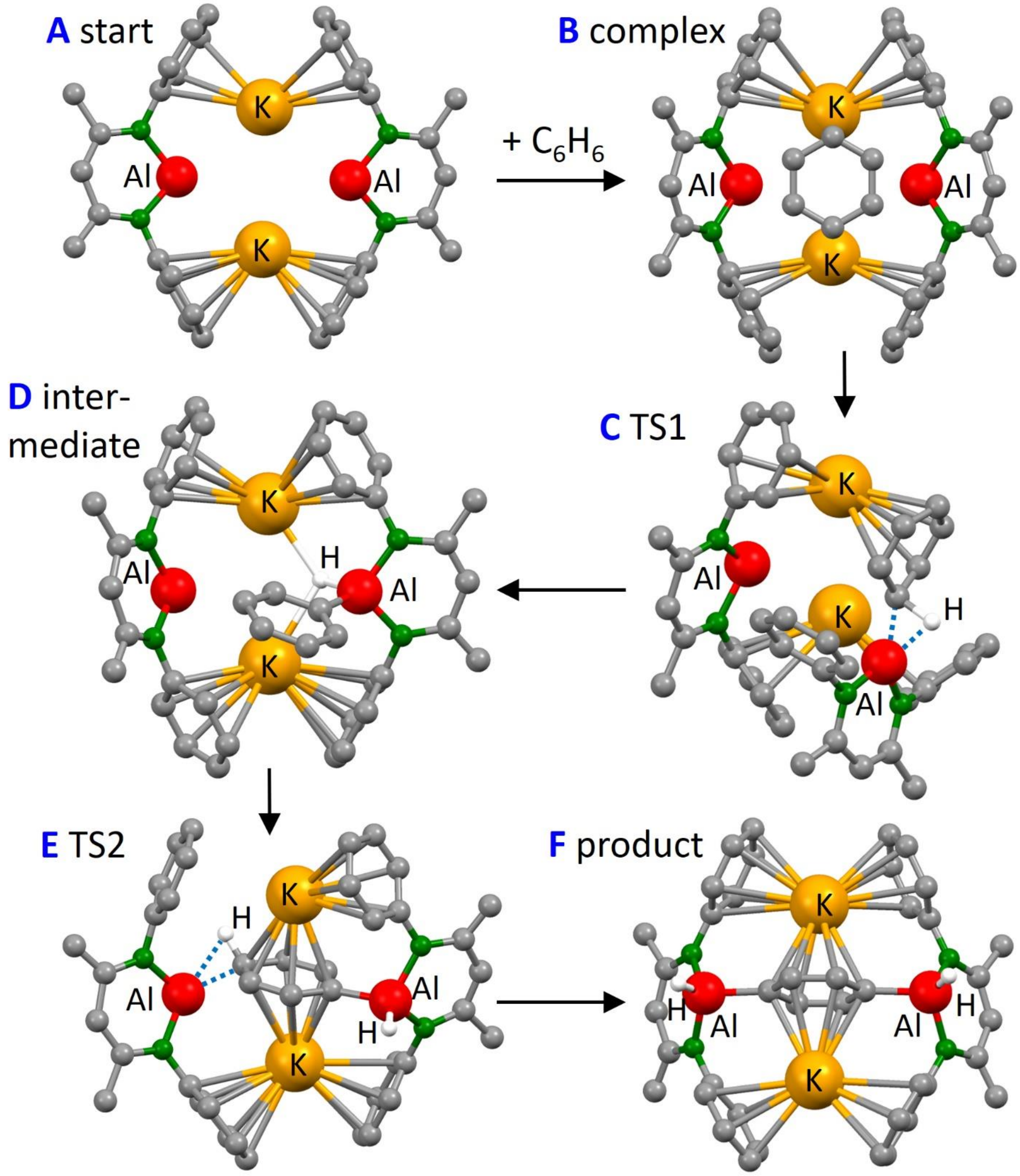

Figure S45. Optimized complexes on the pathway for double C-H activation of benzene by the dimer [(^{Ph}BDI-H)$Al^{-}K^{+}$]

3.3 XYZ-Files

146
[(BDI-H)Al-K]2 complex **1**

K	-0.000716	-2.245825	0.003315
K	0.000744	2.499315	0.005052
Al	2.554329	0.123014	-0.462160
Al	-2.555574	0.124819	0.463831
N	3.775401	-1.321174	-0.570300
N	3.778346	1.371075	0.220240
N	-3.777417	1.373244	-0.221729
N	-3.777552	-1.318736	0.569734
C	5.992201	-2.409807	-0.701981
C	5.754076	-0.056348	0.034479
C	3.181728	-2.606786	-0.659684
C	2.645383	-3.060335	-1.887221
C	1.924036	-4.260245	-1.913238
C	1.745839	-5.022300	-0.765252
C	2.296369	-4.585373	0.437108
C	3.008675	-3.386107	0.512310
C	2.841789	-2.285282	-3.180129
C	3.616416	-3.116216	-4.212313
C	1.507269	-1.792639	-3.757086
C	3.514949	-2.889271	1.859834
C	4.217288	-3.980175	2.675456
C	2.363503	-2.261419	2.658351
C	3.189318	2.602708	0.616311
C	3.080874	3.661922	-0.313018
C	2.386015	4.819505	0.051534
C	1.824835	4.953488	1.318155
C	1.957395	3.917915	2.237790
C	2.633907	2.737587	1.909311
C	3.673453	3.543454	-1.706981
C	2.564292	3.402061	-2.760250
C	4.608685	4.713714	-2.032220
C	2.737760	1.625779	2.941057
C	1.349707	1.125627	3.366375
C	3.568647	2.070189	4.151416
C	-5.937273	2.209112	-0.946838
C	-5.152511	1.229151	-0.426558
C	-5.754284	-0.052989	-0.039565
C	-5.151584	-1.195025	0.378067
C	-5.995206	-2.406027	0.697399
C	-3.186533	2.604210	-0.617089
C	-3.080225	3.664098	0.311726
C	-2.383173	4.820779	-0.051482
C	-1.817549	4.953138	-1.316304
C	-1.948117	3.917003	-2.235568
C	-2.626943	2.737621	-1.908442
C	-3.677466	3.547252	1.703841
C	-4.612525	4.718721	2.025205

C	-2.571896	3.405475	2.760828
C	-2.729058	1.625448	-2.939948
C	-3.558220	2.069576	-4.151614
C	-1.340442	1.125138	-3.363083
C	-3.184669	-2.604670	0.659683
C	-2.650383	-3.058833	1.887904
C	-1.929822	-4.259201	1.914699
C	-1.750196	-5.020994	0.766755
C	-2.298498	-4.583330	-0.436347
C	-3.010094	-3.383688	-0.512288
C	-2.848077	-2.283914	3.180692
C	-1.514041	-1.792369	3.759763
C	-3.624817	-3.114556	4.211535
C	-3.513788	-2.886173	-1.860522
C	-2.360308	-2.259902	-2.657319
C	-4.216352	-3.976201	-2.677132
C	5.940356	2.206213	0.939845
C	5.153861	1.226357	0.421977
C	5.149880	-1.198208	-0.381472
H	7.055280	-2.179114	-0.615652
H	6.836472	-0.079828	0.103840
H	1.504718	-4.609087	-2.853456
H	1.187160	-5.952235	-0.805843
H	2.153220	-5.181982	1.333694
H	3.447595	-1.407939	-2.937821
H	4.568863	-3.466708	-3.803169
H	3.828094	-2.514230	-5.101846
H	3.045742	-3.995099	-4.532939
H	0.849195	-2.630481	-4.017217
H	1.675495	-1.208373	-4.667792
H	0.984719	-1.148386	-3.041288
H	4.243507	-2.096662	1.674833
H	3.522136	-4.756901	3.012967
H	4.674689	-3.541943	3.567778
H	5.006673	-4.464483	2.092658
H	1.928938	-1.409543	2.120920
H	2.717675	-1.883683	3.622958
H	1.570425	-2.994620	2.855486
H	2.284697	5.630965	-0.664260
H	1.293827	5.861281	1.588682
H	1.525461	4.028469	3.229048
H	4.272046	2.629680	-1.723805
H	1.903026	4.277694	-2.761614
H	1.955289	2.508793	-2.572868
H	2.992738	3.305041	-3.763302
H	5.389359	4.804539	-1.271824
H	4.069982	5.666562	-2.089717
H	5.093168	4.549676	-3.000177
H	3.267411	0.794465	2.467737
H	0.759760	1.919287	3.839274
H	1.439178	0.308419	4.089709
H	0.778802	0.746438	2.509207

H	3.690223	1.238679	4.853859
H	3.086579	2.894820	4.689350
H	4.562096	2.397697	3.832801
H	-5.541164	3.173951	-1.241309
H	-6.998913	2.038386	-1.082173
H	-6.836557	-0.075859	-0.111049
H	-5.795914	-2.756298	1.716727
H	-7.057993	-2.174785	0.608966
H	-5.770255	-3.241830	0.025393
H	-2.283560	5.632782	0.663937
H	-1.284657	5.860147	-1.585758
H	-1.512697	4.026318	-3.225431
H	-4.277064	2.634119	1.719414
H	-5.390602	4.809847	1.262189
H	-5.100365	4.555843	2.991669
H	-4.073022	5.671048	2.083831
H	-1.909618	4.280347	2.763619
H	-3.003710	3.309810	3.762563
H	-1.963358	2.511319	2.576183
H	-3.259449	0.794267	-2.467222
H	-3.075500	2.894371	-4.688716
H	-3.678428	1.238027	-4.854254
H	-4.552264	2.396782	-3.834543
H	-0.770302	0.747286	-2.504824
H	-1.428948	0.306980	-4.085464
H	-0.750155	1.918276	-3.836414
H	-1.512190	-4.608579	2.855468
H	-1.191944	-5.951162	0.807908
H	-2.154044	-5.179595	-1.332954
H	-3.452881	-1.406069	2.937706
H	-0.856701	-2.630729	4.020100
H	-0.990225	-1.147693	3.045241
H	-1.683232	-1.208809	4.670744
H	-4.576907	-3.464259	3.800881
H	-3.055240	-3.993939	4.532747
H	-3.837383	-2.512666	5.100920
H	-4.241586	-2.092624	-1.676594
H	-1.568006	-2.994208	-2.853367
H	-2.712544	-1.881541	-3.622395
H	-1.925185	-1.408735	-2.119220
H	-4.671777	-3.537441	-3.570205
H	-3.521725	-4.753907	-3.013467
H	-5.007296	-4.459376	-2.095511
H	5.545521	3.171483	1.234598
H	7.002205	2.034950	1.072852
H	5.768071	-3.245250	-0.029251
H	5.790801	-2.760341	-1.720801

74

[(^{Ph}BDI)Al-K]$_2$

K	0.000337	-2.177056	0.000468
K	-0.000229	2.326713	0.000939

Al	-2.703449	0.082086	-0.669637
Al	2.703849	0.082728	0.670387
N	-3.893051	-1.387454	-0.466924
N	-3.894481	1.421684	-0.098437
N	3.894181	1.422525	0.098140
N	3.893603	-1.386598	0.466779
C	-5.212890	-1.264686	-0.018341
C	-5.790504	-0.080978	0.298691
C	-5.228259	1.273979	0.312877
C	-3.272443	-2.649309	-0.484753
C	-3.097270	-3.414695	0.684329
C	-2.383080	-4.610159	0.660694
C	-1.805830	-5.067874	-0.526321
C	-1.963255	-4.316994	-1.690796
C	-2.689903	-3.126072	-1.671984
C	-3.303492	2.695678	0.063159
C	-2.973710	3.183624	1.339809
C	-2.278990	4.380206	1.489077
C	-1.894518	5.119640	0.365442
C	-2.227139	4.653304	-0.904362
C	-2.932584	3.455654	-1.053912
C	6.016228	2.312275	-0.693113
C	5.227719	1.275094	-0.314038
C	5.790308	-0.079721	-0.300026
C	5.213163	-1.263540	0.017432
C	6.059214	-2.517285	-0.005705
C	3.302777	2.696346	-0.063282
C	2.972128	3.184028	-1.339811
C	2.277016	4.380408	-1.488850
C	1.892991	5.119896	-0.365098
C	2.226459	4.653822	0.904580
C	2.932314	3.456382	1.053894
C	3.273249	-2.648563	0.484778
C	3.097811	-3.413950	-0.684270
C	2.383886	-4.609567	-0.660419
C	1.807154	-5.067448	0.5267850
C	1.964829	-4.316566	1.6912250
C	2.691217	-3.125491	1.6721980
C	-6.017269	2.311025	0.6912790
C	-6.058611	-2.518654	0.004507
H	-7.107156	-2.267111	0.171484
H	-2.266177	-5.182314	1.576619
H	-1.249176	-5.998908	-0.543514
H	-1.530668	-4.664840	-2.624488
H	-2.030776	4.737346	2.484369
H	-1.350426	6.051224	0.483424
H	-1.945218	5.223869	-1.784538
H	5.671952	3.339174	-0.697162
H	7.040120	2.128021	-0.996256
H	6.842123	-0.116644	-0.562737
H	5.753013	-3.216208	-0.790174
H	7.107621	-2.265461	-0.173126

H	5.977608	-3.051477	0.947551
H	2.028130	4.737338	-2.484050
H	1.348587	6.051321	-0.482898
H	1.944900	5.224438	1.784838
H	2.266777	-5.181712	-1.576323
H	1.250719	-5.998608	0.544148
H	1.532656	-4.664537	2.625063
H	-6.842450	-0.118137	0.560840
H	-5.673241	3.338006	0.695408
H	-7.041321	2.126570	0.993760
H	-5.976468	-3.052881	-0.948685
H	-5.752528	-3.217445	0.789142
H	-3.273315	2.602504	2.206625
H	-3.515858	-3.045640	1.616255
H	3.205611	3.093855	2.039858
H	2.828190	-2.549088	2.581577
H	3.271379	2.602860	-2.206718
H	3.515971	-3.044774	-1.616340
H	-3.205224	3.092917	-2.039981
H	-2.826668	-2.549656	-2.581386

86

[(^{Ph}BDI)Al-K]$_2$+C6H6

K	-0.000041	-2.181442	-1.167349
K	0.000030	2.244263	-1.092901
Al	2.765008	0.024366	-0.945267
Al	-2.765017	0.024360	-0.945236
N	3.683041	-1.425772	-0.094382
N	3.659613	1.440132	-0.044608
N	-3.659558	1.440203	-0.044638
N	-3.683058	-1.425698	-0.094231
C	5.295362	-2.478472	1.496429
C	4.975573	-0.035986	1.413369
C	3.155096	-2.694725	-0.383020
C	3.126327	-3.136287	-1.719774
C	2.491023	-4.324381	-2.078379
C	1.875478	-5.118748	-1.111801
C	1.907862	-4.705458	0.220881
C	2.526528	-3.510902	0.579396
C	3.157433	2.715656	-0.372799
C	3.153340	3.128744	-1.715822
C	2.519456	4.308789	-2.110979
C	1.889582	5.120679	-1.171535
C	1.913292	4.740973	0.173606
C	2.534562	3.561154	0.567919
C	-5.181401	2.369736	1.624930
C	-4.585453	1.320014	1.004079
C	-4.975480	-0.035793	1.413502
C	-4.601468	-1.251431	0.942466
C	-5.295293	-2.478274	1.496744
C	-3.157379	2.715695	-0.372955
C	-2.534459	3.561263	0.567666

C	-1.913215	4.741051	0.173233
C	-1.889565	5.120648	-1.171939
C	-2.519460	4.308683	-2.111297
C	-3.153330	3.128669	-1.716014
C	-3.155183	-2.694685	-0.382851
C	-2.526519	-3.510812	0.579543
C	-1.907913	-4.705398	0.221026
C	-1.875695	-5.118768	-1.111634
C	-2.491343	-4.324454	-2.078188
C	-3.126581	-3.136327	-1.719581
C	5.181553	2.369520	1.624953
C	4.585554	1.319855	1.004058
C	4.601513	-1.251585	0.942276
H	6.172810	-2.184804	2.074424
H	5.743548	-0.044993	2.179283
H	2.493004	-4.636504	-3.118706
H	1.388701	-6.047824	-1.388656
H	1.434733	-5.310732	0.988691
H	2.534541	4.596993	-3.158019
H	1.403405	6.041959	-1.474907
H	1.438126	5.368185	0.922398
H	-5.001341	3.400385	1.347578
H	-5.907359	2.186979	2.408273
H	-5.743408	-0.044735	2.179462
H	-4.652328	-3.074293	2.151155
H	-6.172673	-2.184559	2.074820
H	-5.620800	-3.135002	0.683124
H	-1.438015	5.368325	0.921951
H	-1.403409	6.041907	-1.475403
H	-2.534582	4.596799	-3.158361
H	-1.388960	-6.047868	-1.388492
H	-2.493450	-4.636639	-3.118497
H	5.001509	3.400194	1.347679
H	5.907541	2.186694	2.408252
H	4.652455	-3.074506	2.150882
H	5.620771	-3.135171	0.682745
H	3.660572	2.511410	-2.450028
H	2.549394	3.286576	1.616276
H	-2.549239	3.286763	1.616045
H	-3.660588	2.511273	-2.450149
H	3.622552	-2.533502	-2.473282
H	2.509118	-3.195282	1.616616
H	-2.508967	-3.195124	1.616739
H	-3.622879	-2.533573	-2.473064
H	-1.434699	-5.310632	0.988816
C	-1.207112	-0.614759	2.282222
C	-0.000043	-1.312568	2.242286
C	1.207097	-0.614881	2.282212
C	1.208217	0.776798	2.352403
C	-1.208091	0.776921	2.352410
C	0.000099	1.473942	2.380997
H	-0.000096	-2.399919	2.198660

H	2.158753	-1.134606	2.264748
H	2.158793	1.298387	2.392530
H	-2.158612	1.298609	2.392540
H	0.000152	2.559545	2.445196
H	-2.158823	-1.134384	2.264767

86

TS1 [(^{Ph}BDI)Al-K]$_2$+C6H6

K	1.537981	0.841633	-1.923053
K	-2.187714	2.919752	-0.979964
Al	-1.698194	-0.412697	-2.003981
Al	1.657480	0.467763	1.550232
N	-0.966172	-2.157653	-1.867304
N	-3.374389	-0.807538	-1.261974
N	1.181485	-1.104051	2.289769
N	3.390782	0.228313	0.985454
C	-0.935400	-4.583368	-1.300205
C	-2.949503	-3.200769	-0.946769
C	0.412294	-2.251490	-2.151142
C	1.377481	-2.463792	-1.150034
C	2.736065	-2.455844	-1.455742
C	3.169557	-2.209324	-2.760246
C	2.225127	-1.980914	-3.760581
C	0.862860	-2.007201	-3.460055
C	-4.174607	0.323152	-0.972614
C	-4.310977	0.788720	0.346387
C	-4.995837	1.968845	0.616105
C	-5.557862	2.715879	-0.424814
C	-5.438214	2.257847	-1.735717
C	-4.758626	1.066356	-2.006257
C	1.681119	-3.412757	2.843208
C	2.063082	-2.189453	2.409199
C	3.474586	-1.959840	2.056228
C	4.075979	-0.919045	1.431950
C	5.578049	-0.941221	1.265427
C	-0.157383	-1.272828	2.761984
C	-0.467325	-0.948981	4.084830
C	-1.779110	-1.041429	4.540830
C	-2.788520	-1.471358	3.680061
C	-2.480818	-1.810530	2.363981
C	-1.169366	-1.706357	1.904629
C	4.032740	1.172634	0.152782
C	4.037890	2.527507	0.509913
C	4.574527	3.488472	-0.342994
C	5.132093	3.119328	-1.565255
C	5.142464	1.772462	-1.929210
C	4.592398	0.810754	-1.085341
C	-5.132475	-2.258744	-0.418652
C	-3.866907	-2.060327	-0.868955
C	-1.657167	-3.256977	-1.354225
C	0.552585	1.965889	1.153341
C	-0.846371	1.985621	1.661166

C	-1.466036	3.188694	1.891942
C	0.153609	4.327746	0.522411
C	0.794072	3.120628	0.260377
C	-0.935900	4.412382	1.401293
H	-1.642608	-5.389723	-1.100316
H	-3.387852	-4.137812	-0.620780
H	3.450410	-2.625121	-0.655607
H	4.229561	-2.210420	-2.996551
H	2.545061	-1.803866	-4.783639
H	-5.080046	2.316228	1.641472
H	-6.093222	3.635874	-0.211153
H	-5.885739	2.818145	-2.551704
H	0.660983	-3.631726	3.133042
H	2.412772	-4.207755	2.923463
H	4.132028	-2.762927	2.371174
H	6.016586	0.015891	1.565890
H	6.008834	-1.728581	1.885114
H	5.886500	-1.123716	0.231346
H	-2.012972	-0.774605	5.567005
H	-3.812753	-1.544972	4.033055
H	-3.259896	-2.146940	1.686060
H	5.557834	3.869085	-2.224029
H	5.576785	1.466441	-2.876731
H	-5.858829	-1.458744	-0.345619
H	-5.451518	-3.253485	-0.129992
H	-0.424510	-4.793492	-2.245860
H	-0.177110	-4.600648	-0.510404
H	-3.851476	0.219249	1.148143
H	-4.673211	0.695763	-3.023021
H	0.330579	-0.617451	4.742666
H	-0.936822	-1.952542	0.873150
H	1.064826	-2.629301	-0.123022
H	0.123666	-1.854523	-4.240187
H	3.603125	2.822788	1.461278
H	4.577644	-0.231703	-1.386894
H	4.556488	4.531940	-0.044448
H	1.303650	2.149321	2.241514
H	-1.322389	1.066775	1.991548
H	-2.433648	3.190565	2.393151
H	0.477853	5.219786	-0.014496
H	1.657640	3.143636	-0.401102
H	-1.416970	5.360693	1.615507

86

[(^{Ph}BDI)Al(Ph)H-K_2-(^{Ph}BDI)Al]

K	-0.707150	2.307073	-1.415846
K	0.655694	-2.086138	-1.251019
Al	-2.663616	-0.873269	-1.231885
Al	2.290798	0.641629	0.342851
N	-3.981075	0.282442	-0.466688
N	-2.999304	-2.362208	-0.107896
N	3.894885	-0.222375	-0.006049

N	2.872216	2.395116	0.635804
C	-5.981156	0.849848	0.918756
C	-4.840424	-1.323483	1.137050
C	-3.867699	1.639236	-0.800698
C	-3.931797	2.036688	-2.150008
C	-3.737473	3.366765	-2.520430
C	-3.492388	4.343728	-1.554687
C	-3.430694	3.965189	-0.212490
C	-3.589644	2.633312	0.158978
C	-2.061837	-3.411819	-0.231169
C	-1.940259	-4.108062	-1.441872
C	-0.932940	-5.060166	-1.626590
C	-0.041023	-5.349253	-0.597637
C	-0.166522	-4.676781	0.622725
C	-1.157966	-3.719681	0.803902
C	6.299144	-0.252421	-0.336598
C	5.149665	0.396085	-0.022199
C	5.219254	1.815079	0.358906
C	4.234983	2.711101	0.615698
C	4.616794	4.113451	1.037811
C	3.789099	-1.518623	-0.554227
C	3.493021	-2.617289	0.263177
C	3.285615	-3.883039	-0.289118
C	3.376884	-4.074712	-1.665831
C	3.685766	-2.988218	-2.490572
C	3.887145	-1.724874	-1.941905
C	1.905104	3.390589	0.463443
C	0.790637	3.429128	1.322722
C	-0.227193	4.360638	1.133948
C	-0.164253	5.281477	0.087057
C	0.930606	5.248979	-0.778926
C	1.943486	4.308257	-0.605363
C	-4.146885	-3.584585	1.666174
C	-3.956496	-2.472139	0.912912
C	-4.863200	-0.103591	0.550420
H	-6.745232	0.326146	1.495335
H	-5.625632	-1.517239	1.859938
H	-3.805046	3.643253	-3.568780
H	-3.363487	5.382646	-1.840465
H	-3.237132	4.711977	0.551370
H	-0.859459	-5.584225	-2.575065
H	0.737685	-6.092252	-0.737683
H	0.522874	-4.887084	1.435157
H	6.323494	-1.303391	-0.598698
H	7.241761	0.281101	-0.321140
H	6.234213	2.181923	0.467309
H	4.086996	4.388677	1.956483
H	5.690094	4.173902	1.224222
H	4.366586	4.867532	0.285816
H	3.051794	-4.719890	0.361600
H	3.216424	-5.059028	-2.094115
H	3.768753	-3.128457	-3.564525

H	-0.951855	6.014965	-0.050634
H	0.993385	5.957031	-1.600636
H	-3.569682	-4.491101	1.537252
H	-4.921778	-3.592190	2.423680
H	-5.640853	1.701349	1.514845
H	-6.448218	1.257612	0.015709
H	-2.648714	-3.896630	-2.236486
H	-1.238675	-3.186640	1.744726
H	3.423026	-2.462945	1.334650
H	4.131203	-0.875331	-2.572304
H	-4.156585	1.286360	-2.900876
H	-3.486462	2.343351	1.200409
H	0.748374	2.728395	2.150919
H	2.776397	4.266693	-1.300933
H	-1.067367	4.372801	1.821693
C	-0.299853	-0.124512	1.545704
C	-1.199418	-0.610324	2.495247
C	-0.707276	-1.198813	3.657242
C	0.671989	-1.291587	3.850935
C	1.095521	-0.188533	1.703376
C	1.551211	-0.791128	2.890459
H	-2.270600	-0.558201	2.314610
H	-1.394277	-1.593216	4.400071
H	1.060921	-1.751332	4.755559
H	2.621793	-0.864916	3.075822
H	-0.719167	0.307462	0.635965
H	1.334534	0.638131	-1.017079

86

TS2 [(^{Ph}BDI)Al(Ph)H-K_2-(^{Ph}BDI)Al]

K	-0.104826	2.440381	0.336280
K	0.550643	-3.029128	0.127821
Al	-3.283027	0.126033	0.962999
Al	3.124309	0.229013	-1.095057
N	-4.039619	1.470241	-0.069901
N	-4.329086	-1.326859	0.484719
N	4.204635	-1.262995	-0.588355
N	3.967483	1.582054	-0.046684
C	-5.851591	2.450803	-1.463618
C	-5.851646	0.054020	-0.868676
C	-3.320499	2.666791	-0.272385
C	-2.742224	2.968246	-1.517802
C	-2.028613	4.149447	-1.705595
C	-1.849614	5.042210	-0.646225
C	-2.393949	4.740981	0.601717
C	-3.124026	3.565466	0.787508
C	-3.811313	-2.613716	0.772526
C	-3.297308	-3.419876	-0.258097
C	-2.625507	-4.599968	0.036350
C	-2.449172	-5.002092	1.365965
C	-2.982485	-4.224067	2.389790
C	-3.674972	-3.047813	2.094031

C	6.398365	-2.120121	0.045521
C	5.467981	-1.132861	0.012903
C	5.833873	0.167583	0.589078
C	5.184066	1.358795	0.593655
C	5.894368	2.550176	1.205400
C	3.683474	-2.528611	-0.827991
C	3.743944	-3.584337	0.113766
C	3.166071	-4.822065	-0.147984
C	2.479359	-5.059352	-1.345388
C	2.390454	-4.022212	-2.275873
C	2.979490	-2.783464	-2.025351
C	3.237271	2.741321	0.202839
C	2.914329	3.183685	1.503275
C	2.176624	4.347938	1.708053
C	1.699114	5.089248	0.624805
C	1.971725	4.640006	-0.670201
C	2.724069	3.486947	-0.879577
C	-6.293515	-2.316669	-0.542189
C	-5.492674	-1.251661	-0.302517
C	-5.217814	1.248527	-0.802110
H	-6.853211	2.202319	-1.816851
H	-6.792324	0.046858	-1.408119
H	-1.600060	4.368110	-2.678959
H	-1.291917	5.961049	-0.793559
H	-2.266257	5.429847	1.431350
H	-2.242850	-5.216452	-0.773475
H	-1.929443	-5.927859	1.594636
H	-2.870928	-4.535199	3.424025
H	6.242917	-3.091267	-0.407774
H	7.358606	-1.939670	0.513887
H	6.826046	0.172729	1.027137
H	5.465236	2.861305	2.162236
H	6.944323	2.309615	1.378987
H	5.843887	3.414331	0.534305
H	3.256314	-5.614723	0.590482
H	2.054073	-6.035236	-1.557919
H	1.880621	-4.182894	-3.221992
H	1.133357	6.001205	0.786601
H	1.612268	5.203285	-1.527055
H	-6.096981	-3.297684	-0.127400
H	-7.171349	-2.202929	-1.166729
H	-5.928934	3.285270	-0.757988
H	-5.272505	2.808728	-2.320324
H	-3.426896	-3.095373	-1.285652
H	-4.097562	-2.440005	2.888758
H	4.277695	-3.421556	1.043740
H	2.930316	-2.001203	-2.777709
H	-2.860510	2.259905	-2.332296
H	-3.579713	3.343931	1.747984
H	3.245898	2.596437	2.353550
H	2.955864	3.155975	-1.887749
H	1.966018	4.673554	2.723191

C	0.136784	-0.266227	-1.126725
C	-1.095913	-0.611695	-0.593784
C	-1.330013	-0.665461	0.878537
C	-0.095988	-0.399359	1.659850
C	1.313689	-0.037630	-0.366644
C	1.110056	-0.140095	1.051586
H	-1.921933	-0.842580	-1.265421
H	-1.799658	-1.602580	1.201252
H	-0.156353	-0.453848	2.744270
H	1.974685	0.000730	1.703918
H	0.208328	-0.221084	-2.215270
H	3.094237	0.543460	-2.678248

86

[(^{Ph}BDI)Al(Ph)H-K$_2$-(Ph)HAl(^{Ph}BDI)]

K	-0.457416	-2.747083	-0.782460
K	0.464303	2.803928	-0.923767
Al	3.415825	-0.534496	-0.841961
Al	-3.396261	0.626334	-0.843556
N	3.617299	-2.004810	0.336742
N	4.071221	0.862479	0.247356
N	-3.554388	2.010601	0.427185
N	-4.131523	-0.816088	0.144017
C	4.882058	-3.183015	2.137204
C	5.021476	-0.738898	1.863269
C	2.805104	-3.120213	0.164327
C	2.691788	-3.711112	-1.112421
C	1.892095	-4.832534	-1.321372
C	1.168668	-5.401544	-0.269374
C	1.238680	-4.804610	0.992022
C	2.021046	-3.671218	1.201942
C	3.705965	2.167098	-0.087861
C	3.750606	2.585758	-1.432166
C	3.354908	3.871399	-1.805957
C	2.902532	4.783096	-0.852491
C	2.834020	4.375553	0.485193
C	3.212428	3.091560	0.860624
C	-4.728531	3.011628	2.317123
C	-4.415913	1.950859	1.531960
C	-5.081798	0.670475	1.816011
C	-4.955404	-0.551683	1.244995
C	-5.873620	-1.660865	1.721920
C	-2.757421	3.146492	0.247429
C	-1.909177	3.642517	1.261023
C	-1.114581	4.763504	1.048818
C	-1.114623	5.416779	-0.189637
C	-1.921159	4.917917	-1.211809
C	-2.730827	3.799501	-0.998118
C	-3.728161	-2.112675	-0.141750
C	-3.296011	-3.024581	0.847753
C	-2.916434	-4.323519	0.516933
C	-2.904218	-4.749250	-0.813625

C	-3.275283	-3.841568	-1.810252
C	-3.676111	-2.548592	-1.484388
C	5.630598	1.599521	1.972693
C	4.894226	0.637895	1.363545
C	4.456765	-1.900077	1.450418
H	5.775637	-3.005507	2.737444
H	5.737969	-0.829192	2.672388
H	1.856967	-5.282395	-2.309785
H	0.569414	-6.292324	-0.427185
H	0.669596	-5.218517	1.819891
H	3.431516	4.167395	-2.848599
H	2.625246	5.792836	-1.137931
H	2.477934	5.067090	1.243787
H	-4.336538	4.006799	2.149140
H	-5.416798	2.883281	3.143691
H	-5.834158	0.748641	2.593349
H	-5.376106	-2.387592	2.370017
H	-6.706294	-1.236720	2.285278
H	-6.280274	-2.213600	0.868719
H	-0.486528	5.131446	1.855321
H	-0.506699	6.302266	-0.346009
H	-1.947336	5.416587	-2.176829
H	-2.621561	-5.765729	-1.067148
H	-3.280351	-4.151342	-2.851698
H	5.650035	2.628880	1.637439
H	6.253922	1.342861	2.820673
H	4.115693	-3.592501	2.801020
H	5.111578	-3.955297	1.395411
H	4.141175	1.901025	-2.180113
H	3.159242	2.791258	1.901573
H	-1.909118	3.147388	2.226462
H	-3.385511	3.441722	-1.787854
H	3.282492	-3.301992	-1.927446
H	2.033566	-3.197170	2.177900
H	-3.259980	-2.699532	1.882217
H	-4.003281	-1.867061	-2.265036
H	-2.615056	-5.006065	1.306859
C	-0.693109	0.106838	-2.162288
C	0.692869	-0.129910	-2.163087
C	1.436961	-0.220407	-0.972656
C	0.701863	-0.061880	0.224021
C	-1.424419	0.266779	-0.971098
C	-0.677094	0.172031	0.224888
H	1.208739	-0.243179	-3.116895
H	4.063823	-0.716010	-2.300597
H	1.231296	-0.117748	1.175829
H	-1.194294	0.297223	1.176747
H	-1.217836	0.172959	-3.115734
H	-4.026140	0.866303	-2.301943

73

^{DIPP}BDI-Al

Al	-0.000002	-0.224876	-0.978596
N	-1.389161	-0.137133	0.451449
N	1.389159	-0.137144	0.451450
C	-0.000002	-0.204400	2.408410
H	-0.000002	-0.231891	3.490488
C	-1.249421	-0.160683	1.777638
C	-2.714730	-0.010850	-0.092265
C	1.249418	-0.160697	1.777638
C	2.714731	-0.010874	-0.092261
C	-3.217721	1.274513	-0.367307
C	-3.453079	-1.166898	-0.404298
C	-2.474853	-0.124989	2.657682
H	-3.053317	0.784734	2.472344
H	-2.201545	-0.161352	3.712738
H	-3.134642	-0.967624	2.432232
C	2.474850	-0.125011	2.657684
H	3.134667	-0.967614	2.432193
H	2.201543	-0.161431	3.712739
H	3.053284	0.784740	2.472390
C	-2.422910	2.534595	-0.053604
H	-1.526942	2.239478	0.500519
C	3.217753	1.274488	-0.367260
C	3.453053	-1.166928	-0.404337
C	-5.218530	0.249672	-1.277963
H	-6.193459	0.350735	-1.744691
C	-4.476113	1.380254	-0.961984
H	-4.879448	2.363272	-1.188299
C	-4.706280	-1.010951	-0.999188
H	-5.289216	-1.891161	-1.255028
C	2.422968	2.534575	-0.053514
H	1.527023	2.239462	0.500649
C	-3.208479	3.507596	0.835908
H	-4.089481	3.903729	0.319823
H	-2.578143	4.358626	1.113695
H	-3.551330	3.023639	1.755935
C	4.706256	-1.010990	-0.999225
H	5.289170	-1.891206	-1.255095
C	1.957148	3.223888	-1.343879
H	2.813062	3.558577	-1.940435
H	1.343165	4.100359	-1.110441
H	1.363595	2.542016	-1.961101
C	-1.957145	3.223900	-1.343993
H	-1.363629	2.542020	-1.961240
H	-1.343140	4.100364	-1.110586
H	-2.813082	3.558600	-1.940510
C	4.476143	1.380218	-0.961938
H	4.879502	2.363235	-1.188222
C	-2.914066	-2.563830	-0.133003
H	-1.995620	-2.461886	0.452765
C	5.218533	0.249630	-1.277958
H	6.193463	0.350685	-1.744686
C	2.914011	-2.563857	-0.133082

H	1.995547	-2.461910	0.452658
C	-2.545971	-3.271173	-1.445399
H	-3.432880	-3.414192	-2.072845
H	-2.112176	-4.256149	-1.241825
H	-1.819872	-2.685235	-2.017505
C	2.545952	-3.271175	-1.445501
H	1.819883	-2.685218	-2.017625
H	2.112132	-4.256147	-1.241957
H	3.432880	-3.414203	-2.072917
C	3.208582	3.507568	0.835965
H	3.551489	3.023601	1.755967
H	2.578258	4.358592	1.113797
H	4.089553	3.903713	0.319835
C	3.890284	-3.414400	0.690959
H	4.806265	-3.632509	0.131728
H	3.428462	-4.372118	0.951902
H	4.179988	-2.911801	1.619314
C	-3.890376	-3.414349	0.691019
H	-4.180120	-2.911723	1.619347
H	-3.428567	-4.372061	0.952007
H	-4.806332	-3.632473	0.131755

85

$(^{DIPP}BDI)Al-C6H6$

Al	-0.186579	-0.193472	0.607696
N	0.982332	-1.108968	-0.746328
N	-1.759533	-0.699002	-0.532770
C	-0.695404	-2.185005	-2.087005
H	-0.863651	-2.882480	-2.897762
C	0.635202	-1.954590	-1.718788
C	2.371992	-0.773524	-0.606554
C	-1.822555	-1.527912	-1.573279
C	-2.964182	-0.040659	-0.108387
C	2.911699	0.221557	-1.447166
C	3.150905	-1.384806	0.390511
C	1.706989	-2.671062	-2.505345
H	2.244907	-1.966436	-3.147805
H	1.273459	-3.450020	-3.133893
H	2.447541	-3.118070	-1.837212
C	1.582044	3.451212	1.443615
H	1.406968	4.106511	0.594908
C	2.663568	2.572443	1.433937
H	3.330415	2.532125	0.578630
C	2.880398	1.723269	2.516233
H	3.713531	1.027784	2.494948
C	-3.148564	-1.791383	-2.245529
H	-3.838935	-2.282247	-1.553307
H	-3.023368	-2.425297	-3.124029
H	-3.621431	-0.852538	-2.546820
C	2.065414	0.904419	-2.513845
H	1.187117	0.281226	-2.702819
C	-3.217065	1.271147	-0.549157

C	-3.831741	-0.687044	0.793227
C	5.021703	0.013775	-0.269715
H	6.053144	0.324387	-0.134255
C	0.931187	2.627033	3.615883
H	0.248026	2.637889	4.459531
C	0.714851	3.477834	2.533711
H	-0.135503	4.153320	2.535459
C	4.240653	0.599539	-1.260124
H	4.673787	1.369538	-1.891711
C	2.014694	1.751469	3.607441
H	2.176219	1.080013	4.445645
C	4.477221	-0.971645	0.541433
H	5.093725	-1.430295	1.309842
C	-2.268869	2.013578	-1.480700
H	-1.422926	1.354672	-1.699925
C	2.800058	1.063007	-3.850276
H	3.623755	1.781406	-3.782472
H	2.109014	1.432552	-4.614621
H	3.214409	0.110551	-4.196226
C	-4.971826	-0.005336	1.219862
H	-5.650727	-0.484589	1.919149
C	-1.708365	3.272009	-0.803514
H	-2.506597	3.981073	-0.558542
H	-1.001009	3.780528	-1.466928
H	-1.185462	3.014133	0.123191
C	1.548242	2.256647	-2.007404
H	0.992612	2.141804	-1.071474
H	0.885827	2.717424	-2.748670
H	2.379726	2.945124	-1.816354
C	-4.375531	1.909697	-0.100720
H	-4.591639	2.920331	-0.436005
C	2.601171	-2.481115	1.290129
H	1.570156	-2.679919	0.982196
C	-5.251355	1.280396	0.772986
H	-6.145131	1.793794	1.113827
C	-3.532121	-2.075105	1.340932
H	-2.720618	-2.505616	0.746950
C	2.565335	-2.035128	2.757897
H	3.574406	-1.826854	3.132451
H	2.130509	-2.821391	3.384173
H	1.962544	-1.129696	2.872463
C	-3.035569	-1.981432	2.791603
H	-2.152281	-1.338986	2.864003
H	-2.773903	-2.974351	3.173194
H	-3.812567	-1.560927	3.439991
C	-2.936177	2.354152	-2.819930
H	-3.314389	1.455446	-3.317380
H	-2.217460	2.836756	-3.490777
H	-3.778874	3.040627	-2.683208
C	-4.731855	-3.025921	1.236109
H	-5.550078	-2.720028	1.896544
H	-4.435860	-4.038007	1.530180

H	-5.127411	-3.069107	0.216186
C	3.387866	-3.789854	1.130286
H	3.412850	-4.119305	0.086671
H	2.927393	-4.584126	1.726816
H	4.423631	-3.677773	1.468746

85

TS (^{DIPP}BDI)Al + C6H6

Al	-0.000002	0.451331	0.322012
N	-1.415233	0.708664	-0.895148
N	1.415218	0.708661	-0.895164
C	-0.000015	1.626057	-2.612466
H	-0.000018	2.126888	-3.571582
C	-1.259261	1.293303	-2.081960
C	-2.711202	0.267854	-0.450356
C	1.259237	1.293297	-2.081974
C	2.711188	0.267849	-0.450374
C	-3.109932	-1.049452	-0.735216
C	-3.494606	1.126186	0.340329
C	-2.484809	1.623047	-2.891731
H	-3.087101	0.727961	-3.066777
H	-2.215199	2.065517	-3.850521
H	-3.117995	2.325233	-2.339769
C	0.000016	-0.821016	1.784071
H	0.000002	-1.329354	0.538753
C	-1.224746	-1.225855	2.454049
H	-2.186363	-0.893578	2.071048
C	-1.196507	-2.052920	3.560659
H	-2.146677	-2.336316	4.009543
C	2.484775	1.623043	-2.891760
H	3.117891	2.325342	-2.339859
H	2.215148	2.065396	-3.850599
H	3.087143	0.727989	-3.066699
C	-2.231816	-2.018289	-1.512782
H	-1.280278	-1.520239	-1.721795
C	3.109906	-1.049465	-0.735218
C	3.494602	1.126185	0.340296
C	-5.148139	-0.638201	0.514000
H	-6.101781	-0.993235	0.891730
C	1.196577	-2.052918	3.560634
H	2.146757	-2.336311	4.009499
C	1.224792	-1.225856	2.454022
H	2.186403	-0.893589	2.070994
C	-4.347276	-1.476420	-0.250084
H	-4.680295	-2.488958	-0.457771
C	0.000041	-2.507654	4.130297
H	0.000051	-3.147878	5.005188
C	-4.717389	0.648361	0.812969
H	-5.336737	1.286898	1.435432
C	2.231783	-2.018305	-1.512771
H	1.280236	-1.520265	-1.721761
C	-2.863065	-2.402011	-2.858301

H	-3.817885	-2.919248	-2.714012
H	-2.198464	-3.074351	-3.410582
H	-3.050215	-1.523740	-3.484300
C	4.717386	0.648357	0.812933
H	5.336743	1.286898	1.435383
C	1.915913	-3.266618	-0.676872
H	2.817728	-3.859815	-0.489893
H	1.204693	-3.904728	-1.210757
H	1.481129	-2.998716	0.290886
C	-1.915916	-3.266592	-0.676882
H	-1.481150	-2.998682	0.290880
H	-1.204674	-3.904681	-1.210762
H	-2.817717	-3.859814	-0.489913
C	4.347252	-1.476434	-0.250092
H	4.680262	-2.488976	-0.457769
C	-3.009838	2.509026	0.745997
H	-2.137703	2.756631	0.131957
C	5.148126	-0.638210	0.513975
H	6.101770	-0.993247	0.891701
C	3.009851	2.509032	0.745959
H	2.137699	2.756634	0.131942
C	-2.554047	2.495724	2.214117
H	-3.398967	2.284852	2.878632
H	-2.133565	3.467239	2.494264
H	-1.793457	1.726915	2.393304
C	2.554109	2.495761	2.214095
H	1.793540	1.726943	2.393332
H	2.133620	3.467276	2.494231
H	3.399056	2.284922	2.878586
C	2.863010	-2.402009	-2.858306
H	3.050141	-1.523729	-3.484296
H	2.198403	-3.074348	-3.410583
H	3.817836	-2.919241	-2.714040
C	4.058795	3.600298	0.500001
H	4.933467	3.474740	1.146431
H	3.633012	4.585713	0.713785
H	4.407127	3.595629	-0.537988
C	-4.058781	3.600301	0.500085
H	-4.407130	3.595658	-0.537899
H	-3.632990	4.585709	0.713885
H	-4.933443	3.474732	1.146527

85

(^{DIPP}BDI)Al(Ph)H

Al	-0.039172	-0.799693	0.696919
N	-1.386633	-0.800367	-0.688345
N	1.424314	-0.949127	-0.539086
C	0.034889	-2.062087	-2.157876
H	0.041550	-2.701933	-3.030935
C	-1.214055	-1.517379	-1.791623
C	-2.657426	-0.197647	-0.402761
C	1.283456	-1.719934	-1.622858

C	2.707637	-0.377028	-0.251772
C	-3.668549	-0.957117	0.211171
C	-2.819948	1.177530	-0.661621
C	-2.383157	-1.783591	-2.706266
H	-3.022147	-2.556862	-2.267947
H	-2.044047	-2.128442	-3.683450
H	-3.001156	-0.891736	-2.831664
C	-0.087757	0.876521	1.744953
H	-0.190250	-2.175185	1.500420
C	-1.294321	1.271168	2.355196
H	-2.185076	0.653268	2.253273
C	-1.406445	2.452866	3.086237
H	-2.358943	2.724188	3.533194
C	2.517000	-2.246460	-2.312671
H	3.193384	-1.437759	-2.597573
H	2.256733	-2.822246	-3.201005
H	3.071701	-2.889690	-1.621162
C	-3.464911	-2.409335	0.618610
H	-2.520680	-2.759153	0.191681
C	3.549311	-0.979417	0.698686
C	3.067914	0.823687	-0.894981
C	-5.077145	1.017065	0.214136
H	-6.025016	1.491634	0.448728
C	0.914993	2.916472	2.664419
H	1.789132	3.550972	2.783571
C	1.013042	1.730876	1.935325
H	1.979370	1.476438	1.506507
C	-4.878706	-0.324213	0.507390
H	-5.673703	-0.889704	0.984725
C	-0.299209	3.284913	3.238394
H	-0.380827	4.208567	3.804191
C	-4.050221	1.759784	-0.357916
H	-4.206120	2.815710	-0.549179
C	3.148105	-2.248905	1.432504
H	2.245587	-2.644941	0.957728
C	-4.583015	-3.326481	0.104172
H	-5.538419	-3.109834	0.593817
H	-4.337578	-4.372483	0.313323
H	-4.734409	-3.219108	-0.974591
C	4.304060	1.392159	-0.585739
H	4.605818	2.313838	-1.075115
C	2.795879	-1.936393	2.894228
H	3.673785	-1.565392	3.435409
H	2.433779	-2.836469	3.400919
H	2.013675	-1.174070	2.964051
C	-3.334443	-2.521392	2.145035
H	-2.502145	-1.917291	2.516155
H	-3.146892	-3.560090	2.436047
H	-4.252397	-2.186505	2.641188
C	4.767788	-0.361871	0.989942
H	5.427274	-0.805466	1.730164
C	-1.680346	2.009221	-1.234082

H	-0.749105	1.578660	-0.853759
C	5.150365	0.809563	0.350245
H	6.103765	1.272611	0.585145
C	2.146920	1.518443	-1.889042
H	1.192852	0.983992	-1.894750
C	-1.713696	3.465275	-0.761296
H	-2.559145	4.018015	-1.185775
H	-0.802579	3.978647	-1.078689
H	-1.768707	3.520045	0.330091
C	1.858503	2.965261	-1.469699
H	1.409956	3.007128	-0.473281
H	1.165502	3.428694	-2.179329
H	2.770246	3.571978	-1.459694
C	4.225161	-3.337904	1.345863
H	4.499482	-3.548053	0.306977
H	3.858353	-4.265289	1.797003
H	5.137252	-3.050393	1.879760
C	2.710006	1.475833	-3.316723
H	3.669479	2.001457	-3.378206
H	2.015575	1.959791	-4.011546
H	2.866390	0.449812	-3.661639
C	-1.634633	1.928158	-2.766307
H	-1.475520	0.902962	-3.112960
H	-0.813237	2.539492	-3.155543
H	-2.569354	2.296821	-3.203837

12
C6H6

C	-1.376989	0.218157	-0.000041
H	-2.449334	0.388097	-0.000273
C	-0.877436	-1.083260	0.000065
H	-1.560282	-1.927396	0.000017
C	0.499580	-1.301433	0.000040
H	0.888148	-2.315261	-0.000071
C	0.877382	1.083291	0.000034
H	1.560573	1.927140	0.000114
C	-0.499526	1.301441	0.000009
H	-0.888513	2.315098	0.000041
C	1.376993	-0.218158	-0.000026
H	2.449379	-0.387900	-0.000317

72
(BDI-H)Al(-)

Al	-0.011262	-0.458461	-0.932800
N	-1.395072	-0.187026	0.382821
N	1.407400	-0.317504	0.394423
C	0.009792	-0.260765	2.375560
H	0.005630	-0.304195	3.460154
C	-1.313576	-0.107136	1.769170
C	-2.701653	-0.025073	-0.156450
C	1.222906	-0.322925	1.765979
C	2.711736	-0.037758	-0.096426

C	-3.144589	1.251790	-0.559304
C	-3.547838	-1.143446	-0.307124
C	-2.388744	0.097017	2.588976
C	2.457839	-0.422035	2.636847
H	3.056017	-1.299702	2.366827
H	2.181052	-0.497371	3.690831
H	3.107928	0.450813	2.510574
C	-2.272582	2.483734	-0.375552
H	-1.316401	2.142991	0.031285
C	3.146657	1.304780	-0.181090
C	3.551764	-1.075560	-0.550546
C	-5.246747	0.282868	-1.290342
H	-6.231004	0.401470	-1.736077
C	-4.415306	1.383746	-1.123848
H	-4.760762	2.365793	-1.439505
C	-4.807844	-0.971597	-0.882279
H	-5.458204	-1.833850	-1.012945
C	2.249815	2.440598	0.290867
H	1.430724	1.998702	0.863451
C	-2.884931	3.450798	0.646338
H	-3.846746	3.845931	0.295025
H	-2.213623	4.301154	0.817260
H	-3.046016	2.940296	1.600417
C	4.794063	-0.753322	-1.103235
H	5.439721	-1.549799	-1.466391
C	1.624042	3.170867	-0.904497
H	2.396941	3.638181	-1.527370
H	0.938327	3.954026	-0.560467
H	1.058994	2.474158	-1.532613
C	-1.990656	3.182462	-1.711210
H	-1.514459	2.491418	-2.414061
H	-1.321017	4.037430	-1.562962
H	-2.913355	3.556286	-2.171735
C	4.396308	1.584599	-0.733946
H	4.728986	2.616881	-0.813268
C	-3.104960	-2.525410	0.143415
H	-2.131165	-2.407387	0.624588
C	5.220218	0.565003	-1.199378
H	6.187846	0.798232	-1.635684
C	3.128736	-2.531701	-0.448170
H	2.185423	-2.548905	0.105302
C	-2.924440	-3.467668	-1.054040
H	-3.874226	-3.626619	-1.580208
H	-2.554545	-4.445320	-0.722021
H	-2.205864	-3.050527	-1.766638
C	2.865186	-3.134178	-1.834376
H	2.097405	-2.559503	-2.361081
H	2.519081	-4.170772	-1.743821
H	3.778226	-3.132333	-2.443179
C	2.976446	3.423227	1.217435
H	3.438177	2.902491	2.062800
H	2.267220	4.156958	1.616164

H	3.763781	3.976846	0.691893
C	4.148147	-3.374396	0.330759
H	5.102933	-3.448511	-0.203628
H	3.768529	-4.392613	0.473480
H	4.351219	-2.943958	1.316903
C	-4.062721	-3.118239	1.184542
H	-4.161144	-2.441285	2.038282
H	-3.679595	-4.078641	1.549458
H	-5.060316	-3.295793	0.763259
H	-2.241532	0.149641	3.662707
H	-3.396486	0.217409	2.210235

84
(BDI-H)Al(-)-C6H6

Al	-0.416603	-0.392014	0.691287
N	0.573155	-1.408780	-0.609116
N	-2.049932	-0.456920	-0.355630
C	-1.289643	-1.993331	-2.070511
H	-1.586900	-2.562935	-2.945296
C	0.117950	-2.142077	-1.701362
C	1.984058	-1.386839	-0.433875
C	-2.244397	-1.210610	-1.498591
C	-3.090388	0.421477	0.053877
C	2.756124	-0.460360	-1.169926
C	2.610875	-2.252642	0.483592
C	0.917155	-2.952128	-2.458219
C	4.585828	3.531429	1.434679
H	5.566597	3.940446	1.206297
C	4.397433	2.150263	1.477015
H	5.224008	1.477216	1.270145
C	3.137853	1.624220	1.757094
H	2.977543	0.549263	1.750163
C	-3.614430	-1.174202	-2.143033
H	-4.388731	-1.529797	-1.454360
H	-3.633378	-1.798915	-3.038631
H	-3.893235	-0.152611	-2.423582
C	2.105664	0.464485	-2.186527
H	1.067284	0.144171	-2.296596
C	-3.077000	1.767997	-0.370126
C	-4.103423	-0.028514	0.928702
C	4.750215	-1.218782	-0.015546
H	5.821062	-1.144565	0.156975
C	2.251965	3.860340	1.958612
H	1.410535	4.525084	2.134168
C	3.510936	4.388209	1.672201
H	3.653534	5.464808	1.628438
C	4.129681	-0.389673	-0.944619
H	4.723806	0.340241	-1.489508
C	2.065699	2.479335	2.004009
H	1.084646	2.049298	2.189934
C	3.989787	-2.142814	0.689353
H	4.476163	-2.798235	1.408541

C	-1.985897	2.298253	-1.289039
H	-1.318922	1.462988	-1.520406
C	2.764391	0.348308	-3.566107
H	3.802633	0.702752	-3.554814
H	2.215927	0.952900	-4.297901
H	2.755364	-0.692228	-3.904638
C	-5.085015	0.867637	1.354021
H	-5.862003	0.528279	2.034814
C	-1.150447	3.379165	-0.590119
H	-1.765852	4.246604	-0.321427
H	-0.342258	3.724531	-1.243657
H	-0.699632	2.982349	0.325133
C	2.092255	1.914755	-1.688193
H	1.547025	1.996841	-0.743515
H	1.605876	2.566937	-2.424077
H	3.107379	2.291338	-1.512986
C	-4.080763	2.631828	0.073976
H	-4.073721	3.670311	-0.249660
C	1.820938	-3.315082	1.228581
H	0.782170	-3.226448	0.899617
C	-5.083195	2.191435	0.928866
H	-5.854477	2.877298	1.269036
C	-4.110012	-1.460886	1.439082
H	-3.398220	-2.023329	0.828397
C	1.856386	-3.091139	2.745358
H	2.878188	-3.173228	3.136953
H	1.239354	-3.838682	3.258129
H	1.470352	-2.097944	2.995328
C	-3.612709	-1.516592	2.890421
H	-2.610905	-1.082977	2.971313
H	-3.570958	-2.553268	3.245361
H	-4.283224	-0.954163	3.552235
C	-2.555772	2.812527	-2.617555
H	-3.129582	2.032802	-3.128562
H	-1.743198	3.127145	-3.282099
H	-3.217795	3.673687	-2.464938
C	-5.481321	-2.135174	1.305335
H	-6.225867	-1.678054	1.967908
H	-5.408005	-3.194508	1.575620
H	-5.861784	-2.069925	0.280561
C	2.300750	-4.725075	0.858452
H	2.238668	-4.877415	-0.223179
H	1.676166	-5.481127	1.348953
H	3.338641	-4.891951	1.173238
H	0.497521	-3.478566	-3.309072
H	1.972307	-3.084827	-2.251133

84

TS (BDI-H)Al(-) + C6H6

Al	0.027864	-0.045802	0.041167
N	-1.422122	0.534427	-0.909681
N	1.442221	0.571166	-0.917326

C	-0.024137	1.518570	-2.641547
H	-0.038380	2.050700	-3.586696
C	-1.238352	1.221773	-2.105165
C	-2.738548	0.333997	-0.391879
C	1.329206	1.248440	-2.134881
C	2.753087	0.342708	-0.398181
C	-3.432481	-0.856956	-0.674894
C	-3.297582	1.305362	0.462798
C	-2.487119	1.664191	-2.828581
H	-3.136432	0.811460	-3.052607
H	-2.231428	2.167506	-3.762548
H	-3.074977	2.351116	-2.209311
C	0.020178	-1.205496	1.525556
H	0.001798	-1.936793	0.495111
C	-1.209516	-1.524226	2.299549
H	-2.124611	-0.951434	2.162485
C	-1.198921	-2.640809	3.105481
H	-2.128537	-2.949685	3.586951
C	2.405892	1.674776	-2.846325
C	-2.820246	-1.952648	-1.531173
H	-1.857338	-1.584480	-1.896188
C	3.438307	-0.842315	-0.720152
C	3.319106	1.286155	0.480429
C	-5.259457	-0.092565	0.726174
H	-6.236944	-0.263352	1.168310
C	1.208195	-2.684290	3.086815
H	2.132183	-3.026720	3.555864
C	1.248697	-1.570275	2.281110
H	2.181555	-1.031669	2.127535
C	-4.694514	-1.047418	-0.108380
H	-5.234154	-1.969697	-0.307222
C	-0.006457	-3.339458	3.401531
H	-0.017148	-4.198572	4.066160
C	-4.558894	1.073958	1.011412
H	-4.994451	1.806200	1.685694
C	2.826407	-1.889920	-1.633138
H	1.863604	-1.501587	-1.975874
C	-3.684410	-2.277294	-2.757161
H	-4.659278	-2.683555	-2.462883
H	-3.186755	-3.027198	-3.381828
H	-3.863455	-1.387786	-3.370428
C	4.569728	1.019156	1.038111
H	5.007724	1.725983	1.737795
C	2.563606	-3.195804	-0.869618
H	3.505115	-3.664936	-0.558167
H	2.027816	-3.907818	-1.507536
H	1.966852	-3.020109	0.031680
C	-2.553009	-3.211909	-0.693712
H	-1.960571	-2.983742	0.198057
H	-2.012609	-3.955921	-1.289143
H	-3.494790	-3.664772	-0.359563
C	4.691063	-1.068037	-0.146724

H	5.222593	-1.988953	-0.372585
C	-2.508271	2.546231	0.847889
H	-1.693495	2.656535	0.126423
C	5.256934	-0.148588	0.726771
H	6.226193	-0.346983	1.175811
C	2.552708	2.536641	0.876089
H	1.730766	2.651516	0.164297
C	-1.880065	2.356537	2.237554
H	-2.660089	2.271383	3.002970
H	-1.238865	3.207722	2.492425
H	-1.278085	1.442734	2.292727
C	1.949909	2.364196	2.278829
H	1.337840	1.457970	2.350349
H	1.324993	3.225723	2.541360
H	2.742241	2.274671	3.031258
C	3.688597	-2.125961	-2.879742
H	3.839193	-1.190379	-3.427263
H	3.198930	-2.842577	-3.549073
H	4.671592	-2.534589	-2.615330
C	3.403993	3.807793	0.787341
H	4.220583	3.805811	1.518900
H	2.784977	4.689169	0.989623
H	3.838107	3.914686	-0.211529
C	-3.340591	3.832045	0.789509
H	-3.813509	3.955008	-0.190594
H	-2.699809	4.701526	0.971915
H	-4.130519	3.842843	1.549067
H	2.258451	2.202402	-3.781700
H	3.421940	1.505579	-2.510975

84

(BDI-H)Al(Ph)H(-)

Al	0.023096	-0.864514	-0.603608
N	1.401728	-0.782293	0.696851
N	-1.441612	-0.927854	0.578699
C	-0.017381	-1.854887	2.343907
H	-0.020453	-2.403091	3.280507
C	1.198759	-1.419738	1.901687
C	2.651365	-0.178252	0.408265
C	-1.354692	-1.618495	1.784857
C	-2.708170	-0.391596	0.240919
C	3.693143	-0.925335	-0.180322
C	2.811200	1.209124	0.629545
C	2.412064	-1.682417	2.767749
H	3.106633	-2.370527	2.274606
H	2.114249	-2.116242	3.724422
H	2.969166	-0.759150	2.960585
C	0.076740	0.777218	-1.758705
H	0.197316	-2.211795	-1.484201
C	1.279472	1.130136	-2.402598
H	2.161836	0.503372	-2.281492
C	1.406166	2.279215	-3.183392

H	2.360947	2.515545	-3.647591
C	-2.447409	-2.038774	2.485742
C	3.505409	-2.389947	-0.545538
H	2.567940	-2.723864	-0.092487
C	-3.530131	-1.029503	-0.708874
C	-3.126976	0.823377	0.832427
C	5.078454	1.065029	-0.226314
H	6.021745	1.547973	-0.467375
C	-0.898191	2.803247	-2.751034
H	-1.763110	3.450063	-2.879849
C	-1.006662	1.649472	-1.971834
H	-1.969826	1.433742	-1.515524
C	4.898114	-0.286929	-0.485388
H	5.704240	-0.855114	-0.943464
C	0.313420	3.126956	-3.357813
H	0.405249	4.027532	-3.960178
C	4.034357	1.804344	0.320161
H	4.171737	2.868376	0.486437
C	-3.124618	-2.354662	-1.331090
H	-2.214871	-2.685367	-0.823753
C	4.639084	-3.281855	-0.021889
H	5.588432	-3.066959	-0.526983
H	4.402528	-4.336482	-0.201377
H	4.796893	-3.144722	1.052748
C	-4.343858	1.381963	0.445510
H	-4.667908	2.319027	0.892666
C	-2.795557	-2.190525	-2.820675
H	-3.681278	-1.876710	-3.387886
H	-2.435594	-3.136509	-3.240549
H	-2.013529	-1.439800	-2.969726
C	3.354201	-2.550055	-2.065125
H	2.493042	-1.985508	-2.431780
H	3.193358	-3.602719	-2.324672
H	4.253227	-2.199093	-2.587317
C	-4.732599	-0.423894	-1.086710
H	-5.358311	-0.902363	-1.836801
C	1.655504	2.038107	1.171356
H	0.736766	1.551692	0.832125
C	-5.144893	0.772536	-0.516314
H	-6.083735	1.229769	-0.817494
C	-2.263635	1.534890	1.861427
H	-1.325987	0.977922	1.936094
C	1.638944	3.467559	0.622521
H	2.479038	4.065461	0.997340
H	0.719479	3.972138	0.932728
H	1.670059	3.464279	-0.471737
C	-1.923957	2.964765	1.424173
H	-1.423074	2.970493	0.451497
H	-1.257333	3.434720	2.156529
H	-2.822697	3.588859	1.348132
C	-4.189474	-3.436766	-1.111138
H	-4.403681	-3.554097	-0.044458

H	-3.835167	-4.399240	-1.497880
H	-5.127946	-3.195975	-1.625975
C	-2.922117	1.524641	3.247025
H	-3.861351	2.093048	3.243381
H	-2.254456	1.979605	3.988506
H	-3.133368	0.497377	3.556777
C	1.626660	2.032439	2.705852
H	1.489247	1.019419	3.093488
H	0.792846	2.642250	3.072526
H	2.558310	2.442359	3.116151
H	-2.320402	-2.532786	3.442879
H	-3.457668	-1.875518	2.128274

73

(BDI-H)Al(-)K

Al	0.109056	-0.169695	-0.599386
N	1.599908	-0.115775	0.541460
N	-1.192408	-0.068309	0.777603
C	0.372534	-0.066536	2.642466
H	0.469239	-0.063872	3.722695
C	1.530390	-0.094577	1.929890
C	2.881621	-0.015521	-0.083817
C	-1.007689	-0.025940	2.162382
C	-2.526870	0.004441	0.308563
C	3.577777	-1.182472	-0.457062
C	3.414154	1.257096	-0.377217
C	2.839697	-0.108957	2.683830
C	-2.037593	0.051252	3.049045
C	3.033265	-2.563384	-0.126206
H	2.193927	-2.424495	0.561247
C	-3.260728	-1.180873	0.075910
C	-3.093142	1.260385	-0.009507
C	5.303041	0.193618	-1.464764
H	6.239822	0.274465	-2.008159
C	4.782095	-1.055866	-1.152520
H	5.322443	-1.948742	-1.455690
C	4.621990	1.339621	-1.071748
H	5.036993	2.314735	-1.312790
C	-2.709870	-2.532623	0.497836
H	-1.785996	-2.340402	1.048051
C	4.069653	-3.446287	0.580802
H	4.907217	-3.698214	-0.079048
H	3.608980	-4.386658	0.901492
H	4.479898	-2.948989	1.465258
C	-4.341103	1.302985	-0.641624
H	-4.778772	2.264819	-0.897586
C	-2.361111	-3.401956	-0.717720
H	-3.247506	-3.603456	-1.333145
H	-1.954166	-4.366647	-0.398123
H	-1.600503	-2.914786	-1.340330
C	2.492133	-3.256821	-1.384450
H	1.717268	-2.648053	-1.862403

H	2.060473	-4.232281	-1.132889
H	3.292620	-3.417548	-2.116116
C	-4.505672	-1.096731	-0.558445
H	-5.071399	-2.005220	-0.750098
C	2.685312	2.528042	0.034555
H	1.875550	2.238482	0.709989
C	-5.042829	0.134098	-0.930310
H	-6.016817	0.184331	-1.408875
C	-2.364718	2.552127	0.323232
H	-1.456989	2.275984	0.864492
C	2.047957	3.212493	-1.183012
H	2.815170	3.533021	-1.897450
H	1.477513	4.096379	-0.875649
H	1.370076	2.528029	-1.704627
C	-1.943390	3.307129	-0.944340
H	-1.269689	2.696105	-1.557591
H	-1.405467	4.224604	-0.685192
H	-2.813130	3.591893	-1.550129
C	-3.672819	-3.256773	1.446385
H	-3.904136	-2.628251	2.310702
H	-3.215341	-4.181859	1.811495
H	-4.613179	-3.524547	0.950270
C	-3.204697	3.438468	1.251018
H	-4.121418	3.788956	0.762162
H	-2.629477	4.320410	1.550645
H	-3.481761	2.889747	2.155315
C	3.591769	3.502552	0.797080
H	4.063610	3.016298	1.656490
H	3.007188	4.352067	1.165721
H	4.388157	3.902086	0.159462
H	2.663430	-0.115525	3.760564
H	3.435224	-0.989900	2.421765
H	3.449240	0.766395	2.436249
K	-2.363005	-0.120333	-2.864147
H	-1.834048	0.083123	4.113033
H	-3.074327	0.084547	2.734651

85

(BDI-H)Al(-)K +C6H6

K	-2.602523	1.003947	-1.292009
Al	0.365389	-0.294889	-0.342721
N	-0.545030	-1.682392	0.581960
N	2.065717	-0.692246	0.363962
C	1.416437	-2.652664	1.649001
C	-0.023666	-2.690084	1.397086
C	-1.952408	-1.650749	0.420662
C	2.343624	-1.760780	1.207757
C	3.114398	0.220366	0.036494
C	-2.745730	-0.926645	1.340817
C	-2.555095	-2.262115	-0.701058
C	-0.789832	-3.664796	1.959554
C	-0.954284	3.918488	-1.079489

C	-1.708736	3.957454	0.093312
C	-3.097480	4.080243	0.029127
C	3.777523	-1.931789	1.652676
C	-2.127307	-0.324923	2.592131
C	3.316622	1.365960	0.833984
C	3.897603	0.010579	-1.116743
C	-4.694296	-1.292954	-0.066508
C	-2.978498	4.145832	-2.384982
C	-1.589565	4.017579	-2.318558
C	-4.106174	-0.745556	1.072653
C	-3.734661	4.172515	-1.210256
C	-3.920082	-2.056511	-0.936148
C	2.489614	1.605670	2.088670
C	-2.880704	-0.743041	3.859316
C	4.850211	0.968237	-1.469942
C	1.462357	2.722752	1.855459
C	-2.025087	1.200932	2.473644
C	4.279954	2.299234	0.446487
C	-1.746744	-3.140092	-1.641111
C	5.041853	2.109139	-0.700097
C	3.702647	-1.224482	-1.982420
C	-1.657167	-2.535660	-3.048382
C	2.975012	-0.867735	-3.286446
C	3.357185	1.904135	3.317371
C	5.021558	-1.951495	-2.273452
C	-2.306927	-4.567698	-1.679718
H	1.778065	-3.448908	2.290616
H	0.122299	3.795732	-1.027391
H	-1.213426	3.880130	1.055709
H	-3.681101	4.108852	0.944139
H	-1.111895	-0.719588	2.667747
H	-5.754724	-1.152786	-0.256373
H	-3.470219	4.235862	-3.349134
H	-1.000716	3.992296	-3.230016
H	-4.718783	-0.177492	1.768352
H	-4.813852	4.282030	-1.260227
H	-4.387961	-2.515430	-1.803718
H	1.933072	0.686784	2.293966
H	-3.897833	-0.334826	3.889129
H	-2.352730	-0.378854	4.746548
H	-2.943481	-1.832769	3.924920
H	5.452195	0.821564	-2.362789
H	1.959917	3.673845	1.630950
H	0.834581	2.865300	2.742933
H	0.814514	2.468093	1.008874
H	-1.404970	1.479278	1.613246
H	-1.561476	1.630907	3.367974
H	-3.015452	1.659962	2.353981
H	4.437826	3.190114	1.049016
H	-0.734664	-3.193723	-1.232781
H	5.786132	2.845060	-0.989770
H	3.061112	-1.913175	-1.425404

H	-2.649756	-2.435548	-3.505579
H	-1.054173	-3.173405	-3.702894
H	-1.181895	-1.548016	-3.018142
H	2.010406	-0.392746	-3.077846
H	2.795097	-1.766468	-3.887114
H	3.571162	-0.170388	-3.886855
H	4.101346	1.117673	3.475738
H	2.732910	1.967442	4.215014
H	3.891272	2.855695	3.218601
H	5.685805	-1.352572	-2.906373
H	4.825754	-2.891654	-2.799679
H	5.560793	-2.184187	-1.349796
H	-2.347634	-4.990931	-0.672295
H	-1.664103	-5.208042	-2.292443
H	-3.315132	-4.598100	-2.109709
H	-0.331201	-4.417922	2.589750
H	-1.860644	-3.724459	1.802414
H	3.877860	-2.802646	2.302402
H	4.445925	-2.058160	0.794317
H	4.129917	-1.048862	2.196765

85

(BDI-H)Al(-)K-C6H6-Meisenheimer

K	3.411871	1.670546	-1.437892
Al	-0.312848	0.142881	0.093149
N	-1.884060	-0.348563	0.843307
N	0.945191	-0.805597	1.002524
C	-0.780606	-1.706428	2.525525
C	-1.897441	-1.170913	1.971671
C	-3.109355	-0.064580	0.155784
C	0.619390	-1.651956	2.087150
C	2.309061	-0.795709	0.591152
C	-3.815370	1.119845	0.431294
C	-3.557310	-0.962965	-0.833456
C	-3.243686	-1.456176	2.585554
C	-0.093528	1.472315	-1.301805
C	0.675839	1.039047	-2.548080
C	1.198185	2.009420	-3.369531
C	1.533922	-2.427744	2.717903
C	-3.332498	2.106145	1.481785
C	3.265978	-0.077196	1.340486
C	2.693172	-1.517168	-0.564757
C	-5.418796	0.520181	-1.287481
C	1.164940	3.621557	-1.566137
C	0.643132	2.679131	-0.706190
C	-4.968346	1.394668	-0.307298
C	1.382850	3.352743	-2.940561
C	-4.715874	-0.651032	-1.545071
C	2.875932	0.756386	2.551079
C	-4.430109	2.467605	2.490995
C	4.044771	-1.534005	-0.923630
C	3.115191	2.249538	2.282305

C	-2.759781	3.368687	0.821107
C	4.608180	-0.130641	0.947898
C	-2.767915	-2.221250	-1.166246
C	5.002445	-0.861874	-0.167604
C	1.668107	-2.285386	-1.388278
C	-1.968207	-2.017417	-2.462395
C	2.067440	-2.474423	-2.853790
C	3.606769	0.304201	3.821455
C	1.330784	-3.636671	-0.741780
C	-3.645964	-3.474239	-1.255783
H	-0.931335	-2.309290	3.413828
H	-3.754328	-0.526601	2.857557
H	-3.136853	-2.072169	3.479359
H	-3.894729	-1.978578	1.876759
H	-1.127631	1.773748	-1.597150
H	0.611862	0.015954	-2.910738
H	1.568862	1.716254	-4.353943
H	-2.523072	1.622095	2.036605
H	-6.315163	0.752667	-1.854263
H	1.503012	4.574833	-1.154913
H	0.537583	2.927741	0.349881
H	-5.519755	2.311103	-0.117018
H	1.773058	4.111119	-3.610348
H	-5.070548	-1.326112	-2.318609
H	1.806075	0.611950	2.716628
H	-5.243287	3.029861	2.019490
H	-4.017442	3.093761	3.288752
H	-4.865033	1.573397	2.948657
H	4.360260	-2.093164	-1.798097
H	2.553035	2.590735	1.405775
H	4.180474	2.458329	2.121441
H	2.790480	2.848876	3.138815
H	-1.950108	3.132512	0.124302
H	-2.366774	4.054790	1.579353
H	-3.536387	3.897768	0.257416
H	5.356452	0.397178	1.533431
H	-2.052072	-2.393217	-0.357078
H	6.051153	-0.914409	-0.445699
H	0.743922	-1.696253	-1.395895
H	-2.640302	-1.901029	-3.319657
H	-1.312960	-2.874656	-2.654655
H	-1.350355	-1.112765	-2.421821
H	2.373745	-1.530676	-3.316123
H	1.211724	-2.859627	-3.416591
H	2.880339	-3.200540	-2.965434
H	3.425549	-0.755533	4.016391
H	3.246069	0.875059	4.682985
H	4.688192	0.466921	3.746130
H	2.218130	-4.279504	-0.721633
H	0.554318	-4.146384	-1.322665
H	0.977657	-3.516413	0.284275
H	-4.229859	-3.613096	-0.340837

H	-3.020386	-4.361419	-1.398707
H	-4.344486	-3.425509	-2.097906
H	1.221461	-3.064703	3.536303
H	2.575185	-2.465872	2.424214

85

TS (BDI-H)Al(-)K +C6H6

K	-0.134032	4.233046	-0.223901
Al	-0.013775	-0.351595	-0.118855
N	1.450978	-1.183440	0.560325
N	-1.428126	-1.200055	0.615707
C	0.061459	-2.827238	1.703540
C	1.272794	-2.321172	1.352074
C	2.755268	-0.648095	0.333464
C	-1.299189	-2.351171	1.407027
C	-2.737487	-0.698906	0.348988
C	3.459727	-0.980531	-0.839092
C	3.289924	0.278912	1.251921
C	2.528580	-3.006511	1.828572
C	-0.033785	1.200721	-1.203540
C	1.184346	1.953227	-1.572920
C	1.158674	2.867647	-2.610910
C	-2.368610	-3.014366	1.910645
C	2.894796	-1.950058	-1.863637
C	-3.425781	-1.111381	-0.806587
C	-3.298513	0.258279	1.216881
C	5.228264	0.544698	-0.177847
C	-1.252767	2.808231	-2.654312
C	-1.273116	1.892230	-1.620162
C	4.694341	-0.370524	-1.074643
C	-0.046724	3.296212	-3.205793
C	4.526025	0.864611	0.978988
C	-2.844265	-2.149532	-1.750169
C	3.832320	-3.140666	-2.104889
C	-4.532382	0.822050	0.891472
C	-2.541695	-1.537433	-3.125175
C	2.572455	-1.227734	-3.179967
C	-4.660372	-0.524758	-1.094939
C	2.503972	0.690968	2.487044
C	-5.211647	0.438073	-0.259781
C	-2.548154	0.716727	2.456197
C	1.822177	2.046880	2.241396
C	-1.904748	2.089486	2.201708
C	-3.758502	-3.375526	-1.868474
C	-3.424113	0.738428	3.712260
C	3.352692	0.726616	3.762045
H	0.082486	-3.727446	2.307604
H	3.159791	-3.300334	0.983351
H	2.284536	-3.896806	2.409425
H	3.130571	-2.337484	2.453477
H	0.001486	0.007644	-1.910656
H	2.140714	1.684537	-1.128828

H	2.099233	3.314515	-2.933305
H	1.958058	-2.345670	-1.461055
H	6.188603	1.009796	-0.379100
H	-2.201182	3.210167	-3.010664
H	-2.230507	1.575967	-1.211256
H	5.243472	-0.610869	-1.980876
H	-0.048941	4.000895	-4.030103
H	4.946712	1.585572	1.674501
H	-1.900100	-2.492209	-1.318416
H	4.779294	-2.822015	-2.554596
H	3.364988	-3.856845	-2.788488
H	4.064622	-3.663241	-1.171673
H	-4.971291	1.572667	1.543417
H	-3.461426	-1.210779	-3.624196
H	-2.052996	-2.274509	-3.771342
H	-1.880120	-0.669123	-3.036956
H	1.897707	-0.381748	-3.016036
H	2.092638	-1.915413	-3.884204
H	3.485162	-0.844535	-3.650983
H	-5.195749	-0.822532	-1.992426
H	1.712970	-0.048368	2.640293
H	-6.170587	0.886376	-0.502288
H	-1.742468	-0.000721	2.632311
H	2.574595	2.828855	2.080262
H	1.199651	2.331311	3.097050
H	1.189558	1.990449	1.347351
H	-1.249627	2.028612	1.324043
H	-1.311601	2.411105	3.065500
H	-2.676426	2.845142	2.006924
H	-3.944105	-3.814995	-0.884094
H	-3.290206	-4.136991	-2.501148
H	-4.723862	-3.116205	-2.318344
H	-4.220662	1.488178	3.647105
H	-2.817553	0.977118	4.592428
H	-3.887625	-0.238766	3.872699
H	3.864926	-0.227189	3.919460
H	2.717672	0.919465	4.632867
H	4.113183	1.514393	3.728303
H	-2.219535	-3.894454	2.524492
H	-3.386147	-2.696542	1.716871

85

(BDI-H)Al(Ph)H(-)K

K	-2.977580	1.856618	1.385686
Al	0.191898	0.594906	-0.960850
N	1.733175	-0.447788	-0.963567
N	-1.085158	-0.802210	-0.950775
C	0.533255	-2.103761	-2.261706
C	1.689565	-1.512084	-1.853184
C	2.900846	-0.225462	-0.187252
C	-0.845184	-1.898176	-1.789356
C	-2.395581	-0.677879	-0.443133

C	3.790302	0.816769	-0.516987
C	3.145436	-1.036701	0.944958
C	3.002056	-1.944955	-2.464568
C	-0.012735	1.739548	0.680106
C	0.082076	1.255719	1.998208
C	-0.123652	2.071387	3.113787
C	-1.812947	-2.768980	-2.186280
C	3.556281	1.698520	-1.732588
C	-3.395074	-0.005486	-1.187597
C	-2.716949	-1.221734	0.828266
C	5.164721	0.231382	1.395335
C	-0.497771	3.950236	1.648705
C	-0.292745	3.113702	0.545917
C	4.907410	1.035130	0.292907
C	-0.425272	3.424857	2.940908
C	4.287195	-0.800124	1.711245
C	-3.086575	0.607198	-2.542897
C	4.768460	1.720783	-2.672600
C	-4.026894	-1.108868	1.307987
C	-3.103595	2.139599	-2.456999
C	3.158292	3.120787	-1.315943
C	-4.688115	0.103867	-0.659865
C	2.188557	-2.158600	1.322335
C	-5.013962	-0.454497	0.572627
C	-1.658223	-1.928629	1.656352
C	1.900370	-2.211019	2.827637
C	-1.780471	-1.638782	3.156741
C	-4.032775	0.095832	-3.635008
C	-1.662230	-3.440710	1.391301
C	2.690631	-3.528812	0.843979
H	0.625443	-2.867759	-3.026194
H	3.406091	-1.134121	-3.082087
H	2.874466	-2.831283	-3.089027
H	3.753785	-2.160673	-1.699361
H	0.050465	1.587193	-2.224997
H	0.328147	0.209934	2.161300
H	-0.033570	1.657947	4.114992
H	2.716672	1.271289	-2.287936
H	6.044332	0.407137	2.007583
H	-0.695857	5.009193	1.503439
H	-0.342425	3.542675	-0.454642
H	5.594313	1.841753	0.049847
H	-0.571176	4.068502	3.803763
H	4.493717	-1.424983	2.575799
H	-2.074234	0.303638	-2.815594
H	5.635019	2.203421	-2.206688
H	4.530101	2.278271	-3.584714
H	5.065260	0.706713	-2.958280
H	-4.285900	-1.549604	2.266257
H	-4.095673	2.513272	-2.169946
H	-2.844483	2.582328	-3.423430
H	-2.359545	2.499708	-1.736305

H	2.279787	3.106959	-0.664137
H	2.921032	3.727610	-2.196525
H	3.972251	3.612793	-0.770199
H	-5.458405	0.608982	-1.237208
H	1.247840	-1.960308	0.800358
H	-6.030671	-0.395773	0.950853
H	-0.696040	-1.546796	1.307569
H	2.779017	-2.526433	3.400691
H	1.107187	-2.939039	3.028586
H	1.584218	-1.237774	3.217350
H	-1.824443	-0.562441	3.363609
H	-0.910426	-2.037042	3.685801
H	-2.667580	-2.107021	3.597271
H	-4.007052	-0.996207	-3.681313
H	-3.720784	0.484345	-4.609472
H	-5.068729	0.414108	-3.467926
H	-2.611948	-3.887572	1.709629
H	-0.853550	-3.923688	1.950850
H	-1.520634	-3.646749	0.327594
H	2.790574	-3.560668	-0.242790
H	1.984813	-4.314807	1.134545
H	3.665760	-3.761210	1.288282
H	-1.555093	-3.610407	-2.818223
H	-2.847083	-2.675266	-1.875591

15
AlH_3-C_6H_5(-)

C	2.373984	0.005963	0.000567
C	1.660913	1.204075	0.000157
C	0.263123	1.185694	-0.000573
C	-0.484103	-0.006801	-0.000540
C	1.671093	-1.199668	0.000204
C	0.274721	-1.193127	-0.000547
H	2.194640	2.153155	0.000174
H	-0.269971	2.136064	-0.001318
Al	-2.523115	0.000083	0.000253
H	2.213307	-2.143901	0.000277
H	-0.243982	-2.152023	-0.001244
H	-3.061918	-0.755447	1.357757
H	-2.990132	1.577778	-0.026612
H	-3.061994	-0.803997	-1.328987
H	3.462162	0.010478	0.001048

18
AlH_3-C_6H_4-AlH_3(2-) meta

C	1.210250	1.419098	-0.000005
C	0.000000	2.116921	0.000000
C	-1.210250	1.419098	0.000005
C	-1.255988	0.011827	0.000004
C	1.255988	0.011827	-0.000004
C	0.000000	-0.636110	0.000000
H	0.000000	3.209552	0.000000

H	-2.145672	1.981769	0.000010
Al	-3.049558	-0.938221	-0.000001
Al	3.049558	-0.938221	0.000001
H	0.000000	-1.729479	0.000000
H	2.145672	1.981769	-0.000010
H	3.256450	-1.892191	-1.338201
H	-3.256449	-1.892193	1.338200
H	4.233232	0.231316	-0.000091
H	3.256521	-1.892038	1.338303
H	-4.233232	0.231316	0.000094
H	-3.256522	-1.892035	-1.338305

18

AlH_3-C_6H_4-AlH_3(2-) para

C	1.464664	0.005892	-0.000206
C	0.695325	1.185066	-0.000407
C	-0.705404	1.180334	-0.001017
C	-1.464664	-0.005802	-0.000939
C	0.705402	-1.180251	-0.000438
C	-0.695324	-1.184983	-0.000964
H	1.206179	2.151614	-0.000520
H	-1.228920	2.139077	-0.001805
Al	-3.490693	-0.000391	0.000849
H	1.228913	-2.138997	-0.000520
H	-1.206170	-2.151535	-0.001584
Al	3.490695	0.000296	0.000732
H	-4.099843	-0.748607	1.350512
H	-4.025881	1.569726	-0.025817
H	-4.101487	-0.797066	-1.319942
H	4.025576	-1.570152	0.000670
H	4.101382	0.773073	-1.334279
H	4.100221	0.772565	1.336548

18

AlH_3-C_6H_4-AlH_3(2-) ortho

C	-2.472209	0.695532	-0.017078
C	-2.471919	-0.696302	0.017065
C	-1.247270	-1.371836	0.033791
C	0.002865	-0.717184	0.010760
C	-1.247824	1.371538	-0.033768
C	0.002538	0.717376	-0.010741
H	-3.411087	-1.252924	0.033445
H	-1.262503	-2.463028	0.065062
Al	1.610153	-1.978140	-0.015546
H	-1.263453	2.462733	-0.065012
H	2.800535	-1.608302	1.057701
H	0.992317	-3.479551	0.398944
H	2.255584	-2.146635	-1.531459
H	-3.411601	1.251773	-0.033497
Al	1.609572	1.978564	0.015553
H	0.991830	3.479891	-0.399482
H	2.254763	2.147192	1.531526

H	2.800109<br>87	1.608594	-1.057495

$(^{DIPP}BDI)Al(-)+C_6H_5AlH_3(-)$

Al	-0.153281	0.243957	0.071453
Al	3.060912	4.853726	-1.571195
N	0.981638	-0.897668	1.045913
N	-1.808810	-0.437212	0.685676
C	-0.793681	-1.811215	2.429872
C	0.516737	-1.683370	2.078951
C	2.258152	-1.184101	0.482746
C	-1.991390	-1.283892	1.764573
C	-2.967667	0.082847	0.051775
C	2.357593	-2.170024	-0.520886
C	3.387739	-0.440231	0.871105
C	1.553081	-2.440196	2.882728
C	0.140126	0.785664	-1.781865
C	1.579042	0.833042	-2.166109
C	2.379143	1.902469	-1.956303
C	-3.216832	-1.677483	2.231284
C	1.151789	-3.006533	-0.928845
C	-3.377977	-0.449252	-1.191605
C	-3.664787	1.166231	0.626535
C	4.701189	-1.616154	-0.795625
C	0.646491	3.210852	-0.945817
C	-0.189962	1.994904	-0.792656
C	3.588637	-2.374144	-1.144266
C	1.897648	3.214270	-1.470496
C	4.596014	-0.661232	0.207899
C	-2.605204	-1.599540	-1.820954
C	1.351159	-4.484873	-0.565272
C	-4.777413	1.684011	-0.038958
C	-2.680939	-1.627059	-3.349373
C	0.809044	-2.848693	-2.414539
C	-4.497083	0.098518	-1.821697
C	3.302444	0.591658	1.983807
C	-5.200261	1.154746	-1.251660
C	-3.214014	1.793626	1.936948
C	3.318435	2.022920	1.437738
C	-2.709611	3.228210	1.728966
C	-3.029893	-2.950117	-1.228860
C	-4.321068	1.744839	2.998600
C	4.404731	0.386252	3.033554
H	-1.003988	-2.424557	3.300423
H	2.119237	-3.130808	2.248678
H	1.077203	-3.011248	3.683396
H	2.282408	-1.751618	3.323974
H	-0.531646	0.669500	-2.648547
H	2.009943	-0.050051	-2.644617
H	3.433211	1.812672	-2.225400
H	0.290063	-2.647730	-0.360235
H	5.646033	-1.761824	-1.313092
H	0.206527	4.152700	-0.605373

H	-1.261207	2.236034	-0.844897
H	3.674349	-3.121167	-1.930878
H	5.463446	-0.059551	0.466226
H	-1.554423	-1.441925	-1.563172
H	2.195310	-4.919205	-1.116160
H	0.451195	-5.061530	-0.810206
H	1.548582	-4.603924	0.505033
H	-5.311905	2.527707	0.392540
H	-3.680909	-1.901564	-3.710946
H	-1.977767	-2.372082	-3.737410
H	-2.410173	-0.655808	-3.775157
H	0.586942	-1.803132	-2.644042
H	-0.075902	-3.448920	-2.659808
H	1.632322	-3.181970	-3.058571
H	-4.819486	-0.293791	-2.781813
H	2.338510	0.444213	2.480156
H	-6.063128	1.575463	-1.762393
H	-2.376836	1.200292	2.310018
H	4.219014	2.227811	0.848469
H	3.282403	2.745446	2.262081
H	2.465448	2.215310	0.783655
H	-1.861613	3.246810	1.039948
H	-2.379404	3.655351	2.683506
H	-3.499756	3.874277	1.325383
H	-2.932762	-2.949815	-0.139746
H	-2.404578	-3.755551	-1.635947
H	-4.076988	-3.169200	-1.479311
H	-5.185469	2.356504	2.708433
H	-3.944988	2.132337	3.953167
H	-4.655826	0.715107	3.155020
H	4.424380	-0.645854	3.404261
H	4.239349	1.057007	3.884766
H	5.396053	0.616070	2.625317
H	-3.277286	-2.356935	3.074790
H	-4.143118	-1.351198	1.773245
H	2.295297	6.127158	-0.838264
H	4.506139	4.558792	-0.803876
H	3.428327	5.276987	-3.134882

87

TS (^{DIPP}BDI)Al(-)+$C_6H_5AlH_3$(-)

Al	0.052549	-0.205891	0.193703
Al	-0.386729	6.377307	-1.146631
N	-1.355320	-1.315116	0.607436
N	1.488935	-1.290588	0.497863
C	0.098790	-3.117926	1.348162
C	-1.132219	-2.573470	1.151106
C	-2.686981	-0.938166	0.245548
C	1.426660	-2.582002	1.023915
C	2.783838	-0.781576	0.167551
C	-3.421370	-0.055763	1.056890
C	-3.222551	-1.412800	-0.970073

C	-2.351667	-3.374119	1.545445
C	-0.035537	1.649665	-0.196622
C	-1.253594	2.140174	-0.944419
C	-1.320763	3.509658	-1.135394
C	2.527432	-3.352010	1.245059
C	-2.841627	0.547218	2.324999
C	3.517356	-0.062652	1.128431
C	3.285682	-0.961641	-1.134267
C	-5.262594	-0.188089	-0.519135
C	1.039038	3.715117	-0.937369
C	1.182574	2.356183	-0.740629
C	-4.712721	0.298806	0.657765
C	-0.218724	4.397239	-0.961003
C	-4.514090	-1.033582	-1.331566
C	2.946203	0.222585	2.505704
C	-3.587324	0.051934	3.572687
C	4.534843	-0.426523	-1.451886
C	2.607552	1.714899	2.644248
C	-2.818799	2.080349	2.256889
C	4.762603	0.455337	0.771110
C	-2.364500	-2.240445	-1.914639
C	5.275374	0.272605	-0.507027
C	2.460135	-1.648949	-2.208410
C	-1.699569	-1.320549	-2.950740
C	1.954698	-0.615586	-3.226704
C	3.876007	-0.256742	3.627279
C	3.222776	-2.790650	-2.890951
C	-3.130121	-3.375452	-2.603579
H	0.120304	-4.101714	1.805609
H	-3.003372	-2.793805	2.206943
H	-2.056075	-4.294312	2.053602
H	-2.952571	-3.636791	0.667725
H	-0.153913	1.980957	0.912074
H	-2.076525	1.482194	-1.226443
H	-2.255377	3.924349	-1.528292
H	-1.806701	0.203055	2.409371
H	-6.261621	0.114539	-0.820864
H	1.936369	4.291455	-1.186028
H	2.139917	1.852884	-0.878319
H	-5.284278	0.992896	1.267901
H	-4.934066	-1.378277	-2.272451
H	2.017293	-0.346962	2.596911
H	-4.639222	0.362458	3.550559
H	-3.132553	0.472461	4.476849
H	-3.560138	-1.040816	3.652376
H	4.921623	-0.532992	-2.462238
H	3.521239	2.321937	2.637904
H	2.082928	1.902864	3.588050
H	1.982737	2.069557	1.816283
H	-2.334549	2.431045	1.338015
H	-2.273701	2.487607	3.114806
H	-3.836067	2.491368	2.280573

H	5.325689	1.038478	1.495244
H	-1.567087	-2.704273	-1.327335
H	6.237286	0.700963	-0.775595
H	1.591505	-2.096463	-1.716481
H	-2.454877	-0.868905	-3.604095
H	-0.993331	-1.883576	-3.571929
H	-1.162166	-0.486614	-2.484385
H	1.409623	0.204473	-2.742383
H	1.293474	-1.088656	-3.962388
H	2.794324	-0.161369	-3.766441
H	4.090927	-1.325374	3.522331
H	3.404854	-0.092172	4.603507
H	4.827127	0.289815	3.622949
H	4.092408	-2.420712	-3.447654
H	2.569336	-3.306519	-3.604524
H	3.571124	-3.519279	-2.151689
H	-3.641843	-4.012097	-1.873164
H	-2.435011	-4.000017	-3.175755
H	-3.880865	-2.995742	-3.306247
H	2.410448	-4.348749	1.656387
H	3.529164	-3.004275	1.024551
H	-0.549105	7.224092	0.288495
H	0.963506	7.025656	-1.893191
H	-1.739990	6.768416	-2.050412

87

(^{DIPP}BDI)Al(-)Ph-AlH_3(-)

Al	0.001989	-0.637613	-0.971592
Al	-0.837019	6.241989	-0.812640
N	1.560434	-1.345016	-0.136200
N	-1.263621	-1.450267	0.195907
C	0.305381	-3.233444	0.735925
C	1.470338	-2.625377	0.380406
C	2.773597	-0.640463	0.042635
C	-1.054345	-2.669690	0.804078
C	-2.565771	-0.887315	0.246545
C	3.657131	-0.449035	-1.039629
C	3.077191	-0.085727	1.307086
C	2.762369	-3.411285	0.482108
C	-0.145151	1.342226	-0.943378
C	0.837617	2.236671	-0.480549
C	0.635030	3.620997	-0.441777
C	-2.016680	-3.382317	1.468031
C	3.309195	-0.970827	-2.422753
C	-3.560177	-1.317732	-0.656107
C	-2.834422	0.166277	1.147851
C	5.166707	0.770393	0.417655
C	-1.537657	3.333481	-1.335894
C	-1.340265	1.950626	-1.377304
C	4.840517	0.263330	-0.834373
C	-0.559439	4.230739	-0.865895
C	4.283293	0.594067	1.477508

C	-3.277463	-2.403949	-1.682170
C	4.447114	-1.787163	-3.048236
C	-4.100220	0.752570	1.142037
C	-3.321006	-1.838100	-3.107898
C	2.880988	0.181151	-3.342454
C	-4.814942	-0.704791	-0.628132
C	2.084648	-0.177766	2.456873
C	-5.090660	0.321842	0.265846
C	-1.761057	0.645203	2.111472
C	1.769502	1.204742	3.040656
C	-1.804085	2.151671	2.375658
C	-4.228514	-3.597690	-1.528743
C	-1.822046	-0.164834	3.415276
C	2.560113	-1.141680	3.551867
H	0.377063	-4.265972	1.064660
H	3.231225	-3.493064	-0.506640
H	2.578003	-4.415827	0.871235
H	3.489761	-2.913474	1.132241
H	-0.129672	-1.289798	-2.459100
H	1.798604	1.852668	-0.138342
H	1.441410	4.249223	-0.059835
H	2.446870	-1.633938	-2.307517
H	6.092004	1.322314	0.561806
H	-2.499022	3.724427	-1.673346
H	-2.160298	1.327939	-1.741755
H	5.516638	0.428946	-1.670130
H	4.523170	1.021517	2.448352
H	-2.262065	-2.764673	-1.504254
H	5.327248	-1.166601	-3.258056
H	4.118361	-2.225128	-3.997977
H	4.760751	-2.600807	-2.384609
H	-4.309586	1.579454	1.813699
H	-4.319206	-1.453652	-3.354563
H	-3.065437	-2.617879	-3.836052
H	-2.598409	-1.025168	-3.220610
H	2.049049	0.743751	-2.908503
H	2.556676	-0.205061	-4.315652
H	3.711098	0.880823	-3.504372
H	-5.582100	-1.024624	-1.330820
H	1.155043	-0.581577	2.047855
H	-6.065723	0.802830	0.265499
H	-0.800974	0.431465	1.636647
H	2.650773	1.662060	3.507322
H	0.994486	1.118065	3.809753
H	1.401117	1.881730	2.264630
H	-1.774625	2.717636	1.439807
H	-0.933826	2.447101	2.970578
H	-2.697844	2.448424	2.939740
H	-4.159966	-4.008941	-0.517260
H	-3.962049	-4.389380	-2.240119
H	-5.270800	-3.311184	-1.721763
H	-2.780606	0.000934	3.925580

H	-1.018412	0.141634	4.096543
H	-1.719466	-1.235749	3.213380
H	2.704676	-2.150699	3.154200
H	1.813690	-1.201302	4.352315
H	3.508250	-0.803202	3.990631
H	-1.759137	-4.321649	1.946097
H	-3.032900	-3.018891	1.570295
H	-0.668904	6.897261	-2.322161
H	-2.357540	6.584649	-0.266480
H	0.299328	6.902691	0.188801

88
(DIPPBDI)Al(-)(K+)+C6H5AlH3(-)

Al	-0.347031	0.301699	0.344372
Al	3.677366	4.156352	-0.546318
N	0.783762	-1.040681	1.036261
N	-2.017527	-0.520862	0.582044
C	-1.091755	-2.327348	1.941658
C	0.232185	-2.087889	1.789707
C	2.054051	-1.321511	0.461310
C	-2.249930	-1.665791	1.329000
C	-3.172321	0.136714	0.068282
C	2.112003	-2.068466	-0.742055
C	3.249975	-0.895260	1.084044
C	1.204055	-3.003978	2.501487
C	-0.331875	1.451177	-1.267125
C	0.366787	2.131735	-2.366455
C	1.360518	3.019827	-2.137194
C	-3.478878	-2.219139	1.530389
C	0.849039	-2.576051	-1.422351
C	-3.560393	-0.074875	-1.272736
C	-3.911772	1.007886	0.894886
C	4.538811	-1.947848	-0.692302
C	1.032951	2.955313	0.258464
C	-0.270614	2.229554	0.126513
C	3.358377	-2.369391	-1.299138
C	1.850067	3.321065	-0.768549
C	4.475900	-1.213604	0.487197
C	-2.763786	-1.007151	-2.174419
C	0.786982	-4.108650	-1.399575
C	-5.035666	1.646007	0.366298
C	-2.657343	-0.500353	-3.616347
C	0.707597	-2.023377	-2.845234
C	-4.693947	0.579517	-1.757734
C	3.239480	-0.100265	2.378820
C	-5.432575	1.433998	-0.947338
C	-3.530634	1.249093	2.347663
C	3.694547	1.344103	2.139488
C	-3.223204	2.726566	2.623305
C	-3.319344	-2.437026	-2.131482
C	-4.618530	0.734167	3.301211
C	4.102029	-0.760084	3.464656

H	-1.355973	-3.149363	2.598645
H	1.892095	-3.493029	1.803357
H	0.664957	-3.780111	3.047648
H	1.815445	-2.441800	3.214454
H	-1.374590	1.288872	-1.566465
H	0.020247	1.956928	-3.388280
H	1.818443	3.547664	-2.974710
H	-0.007785	-2.217658	-0.848940
H	5.500816	-2.194017	-1.132743
H	1.310667	3.260094	1.270670
H	-1.123885	2.896197	0.321906
H	3.406796	-2.953138	-2.215243
H	5.397090	-0.880420	0.956922
H	-1.749894	-1.041892	-1.769780
H	1.602053	-4.555443	-1.982033
H	-0.162517	-4.451062	-1.823845
H	0.852097	-4.484025	-0.373706
H	-5.610249	2.321509	0.995526
H	-3.619726	-0.540209	-4.140003
H	-1.956736	-1.124353	-4.181692
H	-2.293062	0.531418	-3.647688
H	0.627718	-0.930525	-2.825865
H	-0.206210	-2.411458	-3.307147
H	1.551529	-2.313709	-3.483190
H	-5.003268	0.426960	-2.787623
H	2.202138	-0.074833	2.724591
H	-6.309551	1.939226	-1.342710
H	-2.620456	0.678023	2.545192
H	4.722945	1.388618	1.762574
H	3.656895	1.922074	3.068150
H	3.052712	1.855963	1.419281
H	-2.419246	3.087429	1.977732
H	-2.909080	2.858475	3.665027
H	-4.105251	3.357288	2.458982
H	-3.333754	-2.816031	-1.105739
H	-2.700378	-3.105788	-2.743447
H	-4.343169	-2.466986	-2.525412
H	-5.555269	1.290783	3.171917
H	-4.297937	0.855931	4.342424
H	-4.814605	-0.326665	3.124255
H	3.850032	-1.815722	3.606457
H	3.959588	-0.241380	4.418317
H	5.168391	-0.701224	3.217836
H	-3.572376	-3.116727	2.131037
H	-4.381392	-1.798893	1.103508
H	4.005356	4.470942	1.017841
H	4.712171	2.925254	-1.066577
H	3.899023	5.418019	-1.556021
K	3.170655	0.872261	-1.547595

88

TS (DIPPBDI)Al(-)(K+)+C6H5AlH3(-)

K	-3.936927	1.301686	-0.201924
Al	0.488793	-0.066294	-0.243213
N	2.259400	-0.407108	-0.555884
N	-0.245089	-1.748852	-0.414629
C	1.843834	-2.710383	-1.245498
C	2.663817	-1.643142	-1.068890
C	3.277765	0.434838	0.001292
C	0.424254	-2.871033	-0.914984
C	-1.652164	-1.861189	-0.211932
C	3.823093	1.487003	-0.752917
C	3.715387	0.189542	1.318863
C	4.119048	-1.773182	-1.453236
C	-0.589248	1.426965	0.154334
C	-1.360223	1.472902	1.410276
C	-2.151907	2.558295	1.737735
C	-0.171537	-4.074895	-1.120656
C	3.289375	1.814525	-2.135370
C	-2.526054	-1.785248	-1.316220
C	-2.163914	-2.003098	1.096704
C	5.265366	2.041726	1.116410
C	-1.873016	3.489293	-0.421039
C	-1.065631	2.426739	-0.812721
C	4.816305	2.280661	-0.175838
C	-2.477174	3.624990	0.852941
C	4.711258	1.003515	1.857315
C	-2.006803	-1.569134	-2.728495
C	4.396269	2.021402	-3.175559
C	-3.547540	-2.088025	1.270921
C	-2.392716	-0.173642	-3.238443
C	2.362685	3.037139	-2.056171
C	-3.903283	-1.885516	-1.098147
C	3.052533	-0.882142	2.172998
C	-4.416474	-2.041734	0.185139
C	-1.225198	-2.073135	2.292142
C	2.014362	-0.235515	3.104866
C	-1.862019	-1.619417	3.608243
C	-2.474400	-2.667744	-3.690071
C	-0.617758	-3.475313	2.444588
C	4.050199	-1.731270	2.967581
H	2.293758	-3.592195	-1.688958
H	4.407655	-0.984038	-2.156073
H	4.306848	-2.743681	-1.916150
H	4.771154	-1.670848	-0.579745
H	0.772138	1.684462	0.542039
H	-1.193492	0.706491	2.165308
H	-2.639005	2.530679	2.717046
H	2.689059	0.959273	-2.460267
H	6.035458	2.671912	1.551903
H	-2.139998	4.210643	-1.198715
H	-0.703197	2.396462	-1.840460
H	5.235924	3.108313	-0.741108
Al	-4.202613	4.609232	1.062446

H	5.049962	0.836714	2.876100
H	-0.916833	-1.619132	-2.689398
H	4.997795	2.910967	-2.957354
H	3.956852	2.160532	-4.169304
H	5.072981	1.161082	-3.217129
H	-3.957851	-2.189503	2.270064
H	-2.027367	0.612618	-2.567102
H	-3.482676	-0.073997	-3.328445
H	-1.966185	0.006547	-4.231219
H	1.584532	2.900463	-1.298751
H	1.879285	3.223643	-3.021784
H	2.932026	3.933012	-1.781734
H	-4.583462	-1.844708	-1.946006
H	2.520613	-1.565661	1.505354
H	-5.488602	-2.126406	0.340935
H	-0.393312	-1.391874	2.087030
H	2.506377	0.412286	3.839395
H	1.446876	-1.001246	3.646491
H	1.311014	0.398505	2.550923
H	-2.364727	-0.653073	3.505300
H	-1.085259	-1.515744	4.372833
H	-2.588237	-2.351119	3.981721
H	-2.179680	-3.652841	-3.317553
H	-2.018477	-2.523680	-4.675618
H	-3.562718	-2.655619	-3.823932
H	-1.404234	-4.209461	2.657832
H	0.094501	-3.487495	3.278048
H	-0.098035	-3.788433	1.536508
H	4.804398	-2.173456	2.308643
H	3.523977	-2.544715	3.478560
H	4.570201	-1.145040	3.733535
H	0.406964	-4.900631	-1.518256
H	-1.213688	-4.253835	-0.884942
H	-4.333406	5.913101	0.083391
H	-5.330578	3.454233	0.483827
H	-4.609180	4.899285	2.620382

88

(DIPPBDI)Al(-)(K)Ph-AlH3(-)

Al	-0.532528	-0.497327	-0.967397
Al	3.465335	4.962520	-0.506334
N	0.539024	-1.764851	-0.028566
N	-2.133675	-0.766743	-0.016826
C	-1.450869	-3.073333	0.442694
C	-0.102809	-2.946911	0.322624
C	1.908447	-1.626793	0.275080
C	-2.504406	-2.045867	0.380610
C	-3.144983	0.234768	0.007548
C	2.898312	-1.905869	-0.696437
C	2.298584	-1.186532	1.566298
C	0.762008	-4.177461	0.495604
C	0.258451	1.323220	-0.824609

C	0.497895	1.996757	0.388098
C	1.248176	3.174476	0.459480
C	-3.774282	-2.405950	0.721791
C	2.510146	-2.312846	-2.108119
C	-3.994953	0.415003	-1.102923
C	-3.284545	1.061500	1.144843
C	4.636005	-1.401178	0.929119
C	1.511587	3.148274	-1.900672
C	0.749040	1.975461	-1.972353
C	4.249978	-1.790786	-0.350058
C	1.840088	3.763682	-0.676544
C	3.658962	-1.091534	1.873378
C	-3.876455	-0.458438	-2.341587
C	3.243636	-3.572233	-2.584571
C	-4.271799	2.047584	1.148661
C	-3.393921	0.358254	-3.547520
C	2.732144	-1.148080	-3.082285
C	-4.962330	1.422054	-1.062587
C	1.244660	-0.841812	2.605293
C	-5.108888	2.233194	0.054214
C	-2.390491	0.871845	2.358944
C	1.610848	0.383424	3.449543
C	-1.965212	2.196232	3.003964
C	-5.188974	-1.188968	-2.652667
C	-3.043345	-0.050176	3.399125
C	0.931542	-2.037788	3.516444
H	-1.822635	-4.072509	0.645678
H	1.267931	-4.424781	-0.445118
H	0.158859	-5.035023	0.800414
H	1.546534	-4.024159	1.244887
H	-0.591621	-0.925636	-2.529733
H	0.115260	1.574534	1.313450
H	1.427191	3.618425	1.440257
H	1.437271	-2.522874	-2.097409
H	5.688915	-1.336729	1.189160
H	1.906269	3.570361	-2.825817
H	0.557623	1.533394	-2.951449
H	5.013117	-2.023988	-1.088647
H	3.962747	-0.772901	2.866580
H	-3.122057	-1.221062	-2.136475
H	4.316526	-3.394098	-2.724253
H	2.837900	-3.898814	-3.547642
H	3.130949	-4.393879	-1.870166
H	-4.384508	2.690137	2.017297
H	-4.120798	1.134152	-3.818225
H	-3.248606	-0.293217	-4.416754
H	-2.438866	0.845765	-3.330886
H	2.140858	-0.274819	-2.789938
H	2.417379	-1.424814	-4.093542
H	3.793222	-0.865695	-3.124367
H	-5.612093	1.572492	-1.921572
H	0.331312	-0.617914	2.047835

H	-5.866126	3.012332	0.070954
H	-1.495109	0.358325	1.999598
H	2.430869	0.172795	4.146337
H	0.746369	0.685662	4.049195
H	1.898810	1.237001	2.828085
H	-1.578229	2.900771	2.262015
H	-1.180420	2.017490	3.747686
H	-2.799363	2.677619	3.527900
H	-5.508166	-1.783871	-1.791911
H	-5.051775	-1.864960	-3.504563
H	-5.993434	-0.488575	-2.909055
H	-3.965673	0.400802	3.786967
H	-2.362530	-0.212965	4.244266
H	-3.290518	-1.018912	2.957022
H	0.532106	-2.879426	2.946888
H	0.176123	-1.754227	4.257026
H	1.831430	-2.367068	4.051002
H	-3.979006	-3.419917	1.046921
H	-4.598119	-1.702298	0.706678
H	4.644625	3.766597	-0.343432
H	3.753851	5.789637	-1.874754
H	3.431072	5.841463	0.859443
K	3.503006	1.327894	-0.383375

101

$[(^{DIPP}BDI)AlN(SiMe_3)_2]^{-}K^{+}$

Al	-0.013315	0.142998	-0.457619
N	1.128122	-1.120553	0.721288
N	-1.523787	-0.013850	0.927949
C	-0.227073	-0.956711	2.714607
H	-0.222400	-1.188610	3.773568
C	0.862205	-1.474847	1.963406
C	2.236044	-1.759584	0.057289
C	-1.369970	-0.343521	2.221703
C	-2.834862	-0.020365	0.377678
C	2.028639	-2.931468	-0.692865
C	3.524641	-1.190894	0.160210
C	1.662691	-2.545237	2.678710
C	-2.461931	-0.021815	3.219943
H	-2.280175	0.987457	3.607447
H	-2.448521	-0.710784	4.067031
H	-3.455537	-0.029627	2.768708
C	0.663522	-3.580105	-0.861556
H	-0.080087	-2.925771	-0.397876
C	-3.502793	-1.261533	0.211348
C	-3.432289	1.161829	-0.116629
C	4.406406	-3.025633	-1.167347
H	5.251792	-3.521402	-1.634523
C	3.129760	-3.547620	-1.293941
H	2.983371	-4.458861	-1.867033
C	4.592309	-1.852070	-0.448849
H	5.590721	-1.436180	-0.371095

C	-2.883536	-2.584419	0.644453
H	-1.834167	-2.400063	0.882787
C	0.611984	-4.948924	-0.168315
H	1.343876	-5.638994	-0.602040
H	-0.377196	-5.404882	-0.279582
H	0.831247	-4.864359	0.899785
C	-4.691792	1.087653	-0.721523
H	-5.162193	1.998023	-1.079936
C	-2.917629	-3.624150	-0.481705
H	-3.939167	-3.917190	-0.745793
H	-2.387574	-4.529394	-0.172908
H	-2.421666	-3.247068	-1.381608
C	0.296270	-3.705040	-2.345845
H	0.299880	-2.714818	-2.813062
H	-0.698064	-4.152061	-2.458959
H	1.002253	-4.343531	-2.887183
C	-4.765346	-1.283016	-0.388942
H	-5.290192	-2.228511	-0.492478
C	3.774826	0.109423	0.911512
H	2.870546	0.713405	0.797564
C	-5.366059	-0.119862	-0.855716
H	-6.351772	-0.152756	-1.309941
C	-2.770712	2.518838	0.040384
H	-1.710274	2.346901	0.234886
C	4.943281	0.911999	0.326006
H	5.912326	0.458311	0.563872
H	4.945889	1.917582	0.754869
H	4.867914	1.005803	-0.761183
C	-2.865819	3.372965	-1.227864
H	-2.448836	2.844859	-2.090866
H	-2.290408	4.294660	-1.108153
H	-3.896595	3.663403	-1.458125
C	-3.562605	-3.146524	1.901167
H	-3.508272	-2.444847	2.736040
H	-3.076848	-4.077750	2.210287
H	-4.620262	-3.362891	1.710656
C	-3.369820	3.260175	1.244063
H	-4.426173	3.494923	1.068141
H	-2.835214	4.198303	1.424773
H	-3.309037	2.655043	2.153293
C	4.021801	-0.120745	2.411475
H	3.116580	-0.435076	2.934300
H	4.367759	0.804532	2.884036
H	4.793602	-0.883808	2.567042
H	1.776912	-2.287787	3.734467
H	1.102355	-3.485263	2.633839
H	2.646702	-2.714993	2.244533
N	0.954604	1.828149	-0.262566
Si	1.644020	2.272551	-1.808085
Si	1.011440	3.008950	1.012448
C	0.286461	4.684338	0.486646
H	0.727760	5.075987	-0.435171

H	0.456298	5.425145	1.277236
H	-0.793702	4.613689	0.330013
C	2.728678	3.409354	1.709910
H	3.146874	2.554024	2.248830
H	2.618396	4.226227	2.434111
H	3.453495	3.729503	0.958516
C	0.367215	3.040800	-2.980007
H	-0.419235	2.313427	-3.209849
H	0.836292	3.348937	-3.922369
H	-0.108066	3.919183	-2.530040
C	3.036244	3.558090	-1.701565
H	2.752849	4.497676	-1.218498
H	3.340280	3.796094	-2.728490
H	3.915418	3.162500	-1.184035
C	0.051716	2.523517	2.559060
H	-1.018783	2.412662	2.377995
H	0.185722	3.320837	3.300064
H	0.424222	1.590962	2.993258
C	2.440793	0.827749	-2.733663
H	3.115898	0.249874	-2.094318
H	3.027116	1.222612	-3.574035
H	1.694526	0.130761	-3.123994
K	-2.355993	-0.453059	-2.798786

101

TS [(^{DIPP}BDI)AlN($SiMe_3$)$_2$]$^-$$K^+$

Al	1.610553	-0.591802	-0.846357
N	2.048058	0.870970	0.286221
N	0.069834	-1.096747	0.218586
C	0.121129	0.761024	1.735946
H	-0.415304	1.258736	2.533711
C	1.302929	1.339868	1.322249
C	3.321921	1.480773	0.016739
C	-0.501802	-0.413669	1.233225
C	-0.423539	-2.392603	-0.126433
C	4.473694	0.948627	0.626555
C	3.407477	2.552620	-0.891351
C	1.782733	2.568070	2.052320
C	-1.809207	-0.749809	1.751446
H	-2.553520	0.057613	1.148269
H	-1.944195	-0.474145	2.797893
H	-2.155302	-1.763832	1.552900
C	4.390142	-0.184258	1.640238
H	3.333164	-0.350277	1.868835
C	0.251166	-3.518530	0.405857
C	-1.469626	-2.551635	-1.058405
C	5.819573	2.524703	-0.632083
H	6.794063	2.927218	-0.891284
C	5.716381	1.483846	0.282212
H	6.617938	1.081223	0.735761
C	4.670874	3.055602	-1.206249
H	4.757918	3.876895	-1.911678

C	1.320799	-3.355856	1.476317
H	1.760232	-2.360404	1.358153
C	5.087726	0.169612	2.960185
H	6.169604	0.279601	2.830133
H	4.922168	-0.621117	3.699269
H	4.703793	1.107082	3.373681
C	-1.812805	-3.849211	-1.460788
H	-2.627399	-3.986140	-2.166019
C	2.462469	-4.369871	1.367595
H	2.133571	-5.392856	1.578910
H	3.240556	-4.126705	2.096806
H	2.923830	-4.352227	0.373712
C	4.947289	-1.490718	1.058482
H	4.403289	-1.780974	0.152742
H	4.862199	-2.303785	1.787612
H	6.005022	-1.382708	0.793759
C	-0.125349	-4.793231	-0.028833
H	0.371479	-5.668260	0.377933
C	2.162677	3.146647	-1.529600
H	1.297525	2.775160	-0.977222
C	-1.147499	-4.963327	-0.960047
H	-1.441612	-5.961647	-1.270759
C	-2.203655	-1.364117	-1.655501
H	-1.995048	-0.484669	-1.045444
C	2.007964	2.674289	-2.981352
H	2.845036	3.024536	-3.596489
H	1.077054	3.059218	-3.410285
H	1.982197	1.580745	-3.040331
C	-1.700052	-1.089340	-3.079869
H	-0.641677	-0.796428	-3.081398
H	-2.261513	-0.267081	-3.532373
H	-1.836149	-1.970923	-3.723296
C	0.674274	-3.400401	2.869792
H	-0.099471	-2.634957	2.970666
H	1.428821	-3.230701	3.644595
H	0.211679	-4.377687	3.048803
C	-3.723285	-1.540425	-1.638108
H	-4.065362	-2.310253	-2.340752
H	-4.192693	-0.590753	-1.905088
H	-4.073030	-1.794995	-0.634643
C	2.126536	4.677236	-1.447096
H	2.275376	5.027332	-0.420764
H	1.154973	5.044396	-1.792783
H	2.896106	5.139962	-2.074581
H	1.101052	2.826776	2.862648
H	2.784209	2.412515	2.464668
H	1.847877	3.419313	1.367527
N	-3.463868	1.082573	0.492719
Si	-2.823638	2.488811	-0.202940
Si	-5.029390	0.913355	1.130448
C	-6.401466	0.776862	-0.178192
H	-6.266913	-0.114137	-0.802372

H	-6.402164	1.650624	-0.839660
H	-7.390606	0.710198	0.292121
C	-5.559513	2.334196	2.277999
H	-4.844590	2.449312	3.101383
H	-6.552635	2.159903	2.710335
H	-5.594892	3.288022	1.737524
C	-4.068156	3.463332	-1.260586
H	-4.913667	3.820758	-0.661230
H	-4.474174	2.834061	-2.061570
H	-3.594752	4.338658	-1.722692
C	-2.124112	3.738560	1.047750
H	-2.895965	4.033938	1.767953
H	-1.754481	4.646249	0.553573
H	-1.293342	3.298555	1.611178
C	-5.148170	-0.671660	2.170784
H	-4.861177	-1.557615	1.591882
H	-6.174601	-0.822648	2.525689
H	-4.493993	-0.624144	3.048815
C	-1.379703	2.119598	-1.387038
H	-1.720113	1.604295	-2.291463
H	-0.613680	1.498959	-0.907626
H	-0.903442	3.056339	-1.702053
K	1.098679	-3.306390	-2.798195

28

$KN(SiMe_3)_2$

Si	1.540659	-0.483001	0.001022
Si	-1.540530	-0.483123	-0.001210
N	0.000033	0.195399	-0.000685
C	-1.975589	-1.485173	1.550423
C	-1.928792	-1.600910	-1.484365
C	1.928644	-1.598680	1.485850
C	1.975840	-1.487087	-1.549238
C	-2.831532	0.933014	-0.070312
C	2.831684	0.933169	0.068602
H	-1.322009	-2.361007	1.637680
H	-3.012804	-1.841805	1.533986
H	-1.834883	-0.883362	2.455956
H	-1.738127	-1.077934	-2.428685
H	-2.972187	-1.939313	-1.483749
H	-1.289684	-2.491746	-1.470692
H	1.738617	-1.074059	2.429389
H	2.971799	-1.937821	1.485480
H	1.288904	-2.489084	1.473817
H	1.322295	-2.363074	-1.635273
H	3.013087	-1.843586	-1.532551
H	1.834856	-0.886419	-2.455487
H	-2.761515	1.590612	0.809011
H	-3.854554	0.540234	-0.082233
H	-2.718918	1.541093	-0.979922
H	2.760881	1.590622	-0.810758
H	3.854654	0.540205	0.079547

H	2.720129	1.541426	0.978221
K	-0.000217	2.728261	-0.000109

28

$HN(SiMe_3)_2$

Si	1.582370	-0.009402	0.090487
Si	-1.582366	0.009402	0.090489
N	0.000003	0.000009	0.827675
C	-2.068125	-1.700190	-0.532203
C	-1.588523	1.194948	-1.371855
C	1.588431	-1.194846	-1.371938
C	2.068211	1.700209	-0.532093
C	-2.816259	0.569942	1.394508
C	2.816258	-0.570089	1.394446
H	-1.359684	-2.057916	-1.287124
H	-3.065835	-1.690676	-0.985992
H	-2.074956	-2.426604	0.287593
H	-1.339301	2.212574	-1.053701
H	-2.575128	1.217528	-1.848001
H	-0.863694	0.895739	-2.137315
H	1.339148	-2.212478	-1.053856
H	2.575036	-1.217452	-1.848083
H	0.863624	-0.895535	-2.137378
H	1.359733	2.058043	-1.286930
H	3.065887	1.690670	-0.985956
H	2.075151	2.426556	0.287761
H	-2.811280	-0.102020	2.260305
H	-3.834220	0.576488	0.989939
H	-2.587281	1.580828	1.747888
H	2.811297	0.101791	2.260307
H	3.834218	-0.576618	0.989874
H	2.587254	-1.581001	1.747734
H	0.000001	0.000171	1.840361

4. Single crystal X-ray diffraction

4.1 Structure determination of 1·(*m*-xylene)$_2$

A colorless crystal of compound **1**·(*m*-xylene)$_2$ was embedded in inert perfluoropolyalkylether (viscosity 1800 cSt; ABCR GmbH) and mounted using a Hampton Research CryoLoop. The crystal was then flash cooled to 100.0(2) K in a nitrogen gas stream and kept at this temperature during the experiment. The crystal structure was measured on a SuperNova diffractometer with Atlas detector using a CuKα microfocus source. The measured data was processed with the CrysAlisPro (v40.53) software package.[S15] Using Olex2,[S16] the structure was solved with the ShelXT[S17] structure solution program using Intrinsic Phasing and refined with the ShelXL[S18] refinement package using Least Squares minimization. All non-hydrogen atoms were refined anisotropically. All hydrogen atoms were placed in ideal positions and refined as riding atoms with relative isotropic displacement parameters.

Disorder of the ligand backbone and the co-crystallized *m*-xylene over two positions was observed. It was modeled with the help of similarity restraints (SIMU, SADI) and rigid bond restraints (RIGU).[S19] The relative occupancies of the two alternative orientations were refined to 0.566(13)/0.434(13) (ligand backbone) and 0.646(15)/0.354(15) (*m*-xylene), respectively.

The crystal structure data has been deposited with the Cambridge Crystallographic Data Centre. CCDC 1988236 contains the supplementary crystallographic data for complex **1**·(*m*-Xylene)$_2$. This data can be obtained free of charge from The Cambridge Crystallographic Data Centre via www.ccdc.cam.ac.uk/data_request/cif.

Crystallographic and refinement data are summarized in Table S10.

4.2 Structure determination of 2·(C_6H_6)$_3$

A very faintly greenish crystal of compound **2**·(C_6H_6)$_3$ was embedded in inert perfluoropolyalkylether (viscosity 1800 cSt; ABCR GmbH) and mounted using a Hampton Research CryoLoop. The crystal was then flash cooled to 100.0(2) K in a nitrogen gas stream and kept at this temperature during the experiment. The crystal structure was measured on a

SuperNova diffractometer with Atlas detector using a CuKα microfocus source. The measured data was processed with the CrysAlisPro (v40.53) software package.(S15) The measured crystal was twinned by pseudomerohedry (twin law 0 1 0 / -1 0 0 / 0 0 1). The fractional contributions of the two twin domains were refined to 0.5661(11) and 0.4339(11) later on. Using Olex2,(S16) the structure was solved with the ShelXT(S17) structure solution program using Intrinsic Phasing and refined with the ShelXL(S18) refinement package using Least Squares minimization. All non-hydrogen atoms were refined anisotropically. All hydrogen atoms, except of the hydride ligands H1AL und H2AL, were placed in ideal positions and refined as riding atoms with relative isotropic displacement parameters. The positions of the hydrides were observed from difference Fourier maps and refined isotropically.

The central phenylene moiety and the coordinated $K(C_6H_6)$ fragment show disorder over two positions. The relative occupancies of the two alternative orientations were refined to 0.859(3) and 0.141(3). The distances between Al and the phenylene moiety were restrained to be similar in both orientations (SADI). In case of the minor orientation, it was also necessary to refine the phenylene moiety and the coordinated benzene ligand as rigid hexagons (AFIX 66).

This was also done for the co-crystallized benzene molecules, which are disorder over two positions each. The relative occupancies in these cases are 0.836(8)/0.164(8) and 0.856(6)/0.144(6), respectively.

Additionally, the potassium atom K2 occupies three alternative positions, which show relative occupancies of 0.354(4), 0.344(4) and 0.302(4). The disorder of potassium is the result of a disorder in the ligand back bone (partial exchange of the positions of the CH_3 and CH_2 group). However, this disorder is left untreated. Therefore the bond lengths of the ligand backbone are somewhat unreliable.

For the above described disorder model, similarity restraints (SIMU) were used on all disordered moieties.

The crystal structure data has been deposited with the Cambridge Crystallographic Data Centre. CCDC 1988237 contains the supplementary crystallographic data for complex **2**·$(C_6H_6)_3$. This data can be obtained free of charge from The Cambridge Crystallographic Data Centre via www.ccdc.cam.ac.uk/data_request/cif.

Crystallographic and refinement data are summarized in Table S3.

Table S10: Crystal data and structure refinement for compound **1** and **2**

Compound	1	2
Identification code	hasj191126b	hasj191128b
Empirical formula	$C_{74}H_{100}Al_2K_2N_4$	$C_{88}H_{110}Al_2K_2N_4$
Formula weight	1177.73	1355.95
Temperature/K	100.0(2)	100.0(2)
Crystal system	orthorhombic	orthorhombic
Space group	Pccn	$P2_12_12_1$
a/Å	16.57903(15)	17.2468(3)
b/Å	22.58660(18)	17.2349(2)
c/Å	18.83396(15)	26.5206(3)
α/°	90	90
β/°	90	90
γ/°	90	90
Volume/Å^3	7052.64(10)	7883.2(2)
Z	4	4
ρ_{calc}g/cm^3	1.109	1.142
μ/mm^{-1}	1.738	1.619
F(000)	2544.0	2920.0
Crystal size/mm^3	0.311 × 0.248 × 0.215	0.134 × 0.098 × 0.055
Radiation	Cu Kα (λ = 1.54184)	Cu Kα (λ = 1.54184)
2Θ range for data collection/°	7.828 to 145.406	7.252 to 144.556
Index ranges	-20 ≤ h ≤ 17, -27 ≤ k ≤ 21, -22 ≤ l ≤ 23	-21 ≤ h ≤ 20, -14 ≤ k ≤ 20, -32 ≤ l ≤ 32
Reflections collected	46209	29307
Independent reflections	6973 [R_{int} = 0.0230, R_{sigma} = 0.0124]	14374 [R_{int} = 0.0275, R_{sigma} = 0.0390]
Data/restraints/parameters	6973/852/485	14374/1587/1111
Goodness-of-fit on F^2	1.035	1.056
Final R indexes [I>=2σ (I)]	R_1 = 0.0311, wR_2 = 0.0832	R_1 = 0.0382, wR_2 = 0.0970
Final R indexes [all data]	R_1 = 0.0327, wR_2 = 0.0845	R_1 = 0.0403, wR_2 = 0.0987
Largest diff. peak/hole / e Å^{-3}	0.30/-0.22	0.20/-0.18
Flack parameter	-	-0.008(5)
CCDC number	1988236	1988237

5.References

(S1) M. Stender, R. J. Wright, B. E. Eichler, J. Prust, M. M. Olmstead, H. W. Roesky and P. P. Power, J. Chem. Soc., *Dalton Trans.*, **2001**, 3465–3469.

(S2) C. Cui, H. W. Roesky. H.-G. Schmidt, M. Noltemeyer, H. Hao and F. Cimpoesu, *Angew. Chem. Int. Ed.*, **2000**, 39, 4274–4276.

(S3) P. B. Hitchcock, A. V. Khvostov, M. F. Lappert, *J. Organomet. Chem.*, **2002**, 663, 263–268.

(S4) Kulbitski, K., Nisnevich, G. and Gandelman, M., **2011**, *Adv. Synth. Catal.*, 353, 1438-1442.

(S5) Roman Neufeld and Dietmar Stalke, *Chem. Sci.* **2015**, *6*, 3354-3364.

(S6) M. J. Frisch, G. W. Trucks, H. B. Schlegel, G. E. Scuseria, M. A. Robb, J. R. Cheeseman, G. Scalmani, V. Barone, B. Mennucci, G. A. Petersson, H. Nakatsuji, M. Caricato, X. Li, P. H. Hratchian, A. F. Izmaylof, J. Bloino, G. Zheng, J. L. Sonnenberg, M. Hada, M. Ehara, K. Toyota, R. Fukuda, J. Hasegawa, M. Ishida, T. Makajima, Y. Honda, O. Kitao, H. Nakai, T. Vreven, J. A. Montgomery, J. E. Peralta, F. Ogilaro, M. Bearpark, J. J. Heyd, E. Brothers, K. N. Kudin, V. N. Staroverov, T. Keith, R. Kobayashi, J. Normand, K. Raghavachari, A. Rendell, J. C. Burant, S. S. Iyengar, J. Tomasi, M. Cossi, N. Rega, J. M. Millam, M. Klene, J. E. Knox, J. E. Cross, V. Bakken, C. Adamo, J. Jaramillo, R. Gomperts, R. E. Stratmann, O. Yazyev, A. J. Austin, R. Cammi, C. Pomelli, J. W. Ochterski, R. L. Martin, K. Morokuma, V. G. Zakrezewski, G. A. Voth, P. Salvador, J. J. Dannenberg, S. Dapprich, A. D. Daniels, O. Farkas, J. B. Foresman, J. V. Ortiz, J. Cioslowski, D. J. Fox, Gaussian, Gaussian, Inc., Wallingford CT, **2013**.

(S7) J. Chai, M. Head-gordon, *Phys. Chem. Chem. Phys*. **2008**, 6615–6620.

(S8) W. J. Hehre, L. Radom, P. v. R. Schleyer, J. A. Pople, Ab Initio Molecular Orbital Theory by W. J. Hehre, L. Radom, P. v. R. Schleyer, and J. A. Pople, John Wiley, New York, 548pp. Price: $79.95 (1986), John Wiley, New York, **1986**.

(S9) T. Clark, J. Chandrasekhar, G. W. Spitznagel, P. v. R. Schleyer, *J. Comp. Chem*. **1983**, 4, 294–301.

(S10) E. F. Silva, H. F. Svendsen, K. M. Merz*, J. Phys. Chem. A* **2009**, 6404–6409.

(S11) A. E. Reed, R. B. Weinstock, F. Weinhold, *J. Chem. Phys*. 1985, 83, 735–746.

(S12) N. van Eikema Hommes, Molecule, Erlangen, **2016**.

(S13) T. A. Keith, AIMAll (Version 17.11.14), TK Gristmill Software, Overland Park KS USA, **2017.**

(S14) R. F. W. Bader, *Chem. Rev*. **1991**, 91, 893–928.

(S15) Rigaku Oxford Diffraction, **2019**, CrysAlisPro Software system, version 1.171.40.53, Rigaku Corporation, Oxford, UK.

(S16) O. V. Dolomanov, L. J. Bourhis, R.J. Gildea, J. A. K. Howard and H. Puschmann, *J. Appl. Cryst.*, **2009**, 42, 339–341.

(S17) G. M. Sheldrick, *Acta Cryst. A*, **2015**, 71, 3–8.

(S18) G. M. Sheldrick, *Acta Cryst. C*, **2015**, 71, 3–8.

(S19) A. Thorn, B. Dittrich and G. M. Sheldrick, *Acta Cryst. A*, **2012**, 68, 448–451.
